# No time to wait: resilience as a cornerstone for primary health care across Latin America and the Caribbean, a World Bank-PAHO Lancet Regional Health Americas Commission

**DOI:** 10.1016/j.lana.2025.101240

**Published:** 2025-09-29

**Authors:** Cristian A. Herrera, Ernesto Bascolo, Manuela Villar-Uribe, Natalia Houghton, Sara Bennett, Marcia C. Castro, Adriano Massuda, Sebastian Bauhoff, Myrna Kay Cunningham Kain, J. Peter Figueroa, Walter Flores, Pablo Gaitán-Rossi, E Garcia Elorrio, Ligia Giovanella, Frederico Guanais, Jeannie Haggerty, Stella Hartinger Peña, Daniel Luna, James Macinko, Helia Molina, Diana Pinto, Magdalena Rathe, Maria del Rocio Saenz Madrigal, Renato Tasca, Carina Isabel Vance Mafla, Cristián Mansilla, Victoria Haldane, Anya Abanto, Y. Natalia Alfonso, L. Esther Aranda, Marina Gonzalez-Samano, Claudia Zavaleta Jimenez

**Affiliations:** a Health Nutrition and Population Global Practice, World Bank, Santiago, Chile; b Health Systems and Service Department, Pan American Health Organization, Washington, D.C., USA; c Health Nutrition and Population Global Practice, World Bank, Washington, D.C., USA; d Department of International Health, Johns Hopkins Bloomberg School of Public Health, Baltimore, MD, USA; e Department of Global Health and Population, Harvard T H Chan School of Public Health, Boston, MA, USA; f Fundação Getúlio Vargas, Rio de Janeiro, Brazil; g Health, Nutrition and Population Division, Inter-American Development Bank, Washington, D.C., USA; h Fund for the Development of Indigenous Peoples of Latin America and the Caribbean (FILAC), La Paz, Bolivia; i Department of Community Health and Psychiatry, The University of the West Indies, Kingston, Jamaica; j Accountability Research Center, School of International Service, American University, Washington, D.C., USA; k Research Institute for Equitable Development (EQUIDE), Universidad Iberoamericana, Mexico City, Mexico; l Health Care Quality and Patient Safety, Institute for Clinical Effectiveness and Health Policy, Buenos Aires, Argentina; m Centro de Estudos Estratégicos, Fundação Oswaldo Cruz, Rio de Janeiro, RJ, Brazil; n Employment, Labour and Social Affairs Directorate, Organisation for Economic Co-Operation and Development (OECD), Paris, France; o Department of Family Medicine, McGill University, Montréal, Québec, Canada; p Centro Latinoamericano de Excelencia en Cambio Climático y Salud, Universidad Peruana Cayetano Heredia, San Martin de Porres, Peru; q Health Informatics Department, Hospital Italiano de Buenos Aires, Buenos Aires, Argentina; r Department of Health Policy and Management, Fielding School of Public Health, University of California Los Angeles, Los Angeles, CA, USA; s Chamber of Deputies, Parliament of Chile, Santiago, Chile; t Independent Researcher, Medellin, Colombia; u Fundacion Plenitud, Santo Domingo, Dominican Republic; v Red de la Américas de Equidad en Salud (RAES), Universidad de Costa Rica, San Pedro de Montes de Oca, Costa Rica; w Instituto de Estudos para Politicas de Saude (IEPS), Brasilia, Brazil; x Pan American Health Organization, Santiago, Chile; y Institute of Health Policy Management and Evaluation, University of Toronto, Toronto, Canada; z Health Systems Program, Johns Hopkins Bloomberg School of Public Health, Baltimore, MD, USA; aa Health Research Methods, Evidence, and Impact (HEI), Health Sciences Centre, McMaster University, Hamilton, ON, Canada

## Executive Summary

The Latin America and the Caribbean (LAC) region is particularly vulnerable to multiple shocks, including natural, anthropogenic, and climate change-related, which threaten the health and well-being of millions. One significant way these shocks may adversely impact populations is through the interruption of essential services. A modelled shock causing a 25–50% reduction in primary care coverage with a one-to-five-year recovery period, would result in societal economic costs ranging from US$ 7 billion to more than US$ 37 billion. Conservative estimates show that such a disruption could cause between 32,100 and 164,800 additional deaths, corresponding to 600–3100 stillbirths, 300–1400 neonatal deaths, 2000–10,000 child deaths, 2200–11,300 maternal deaths, and 29,000–149,000 non-communicable disease (NCD)-related deaths, plus 2.7–14.1 million unintended pregnancies. Importantly, the region can expect to experience more than one such shock in the coming years, which could have a multiplicative effect.

Given this high cost of inaction for people and economies, the history of underwhelming responses to past public health emergencies, and the region’s high-risk profile for future shocks (e.g., epidemiological and climate-related), the Commission asserts that it is urgent to put resilience as a cornerstone of primary health care (PHC)-based systems.

The Commission’s work produces three novel contributions to literature. Firstly, it explicitly links the concepts of PHC and resilience, which until recently have mostly been analysed separately. Secondly, it produces a policy framework and actionable policy options linking PHC and resilience for LAC, which could also be considered elsewhere. Thirdly, it models the cost to population health and the economics of inaction on PHC and resilience in LAC. The findings of the Commission show that the region will continue to face accelerating threats and more complex shocks that will put further strain on PHC systems. It underscores that the potential cost of inaction is high and without commitment to resilient PHC millions of lives and livelihoods remain at risk and economies already are and will continue to be hard hit but shocks. We present urgent areas that countries can take action on today that work in synergy to build both PHC-based systems and resilience. These include models of care that ensure services before, during, and after emergencies, ways forward to embed essential public health functions in PHC, opportunities to institutionalize community empowerment and trust, key multisectoral actions, and policies through innovative governance mechanisms, and paths to achieve resilient PHC financing. Importantly, our work draws attention to the political economy scenario in the region and the enablers that underpin PHC-based systems, offering an exploration of contextualised approaches to building PHC resilience based on a LAC worldview and the unique contexts and realities of the region.

The Commission sets out five high-level recommendations that mutually reinforce essential health services coverage and resilience by ensuring that everyone can access and receive the health care they need, in their community, when they need it, without facing financial hardship, along the resilience cycle: before, during, and after shocks. There is no trade-off between the development of PHC-based systems and health system resilience; in fact, they are synergistic and mutually reinforcing.

In the context of mounting geopolitical tension, increasing science scepticism, escalating threats to public health gains, and growing likelihoods of serious public health emergencies, the stakes have never been higher, and the potential cost of inaction threatens millions across the region. However, there is ample reason to be optimistic, the extensive improvements in health and well-being in LAC over the past century show that the region is capable of profound change and innovation. Countries must invest in resilient PHC grounded in integrated health services, essential public health functions, community empowerment, multi-sectoral approaches, all supported by effective and sustainable PHC financing (Panel 1).

Panel 1 **High-level recommendations of the Commission and key enablers identified for resilient PHC-based systems.**

**High-level recommendations of the Commission**

To achieve resilient universal PHC-based systems in LAC urgent action is needed to:

1. Strengthen comprehensive and equitable **models of care that provide services for all across the resilience cycle,** ensuring effective connections between levels of care. Base these models on geographic empanelment and community outreach with multi-professional, culturally sensitive primary care teams that build trusted relationships between health systems and communities and that can be maintained during shocks.
2. Integrate **essential public health functions (EPHF)** into PHC, including effective surveillance, disease prevention, health promotion, and addressing social determinants of health, so that these functions take place close to communities, reinforce PHC-based systems, and build trust before, during, and after shocks.
3. Foster **community empowerment and trust** by institutionalizing meaningful engagement, respecting cultural diversity, ensuring transparent communication and accountability, and integrating communities into decision-making to strengthen PHC capacity in preparing for and responding to shocks. Leverage community assets and resilience to integrate community-driven responses in partnership with local and national governments.
4. Establish **multisectoral action and policies**, introducing innovative intersectoral governance mechanisms and appropriate regulation frameworks, to support a whole-of-society and social determinants of health approach to support effective delivery of essential primary care services and non-health interventions along the resilience cycle, including public-private collaboration with a public good perspective.
5. Ensure sustainable and **resilient PHC financing**, through predominantly public funds, by strengthening governance, efficiently pooling resources, and building adaptable mechanisms for swift resource (re)allocation and payment so that essential services can be maintained across the resilience cycle and PHC activities can be reconfigured and leveraged to respond to emergencies.

**Key enablers identified by the Commission for resilient PHC-based systems**

The Commission also identified five key enablers connected to broader contextual dimensions that must be considered to realize the full potential of resilient PHC. Importantly, historical health system constraints and political economy factors in the region have hindered PHC reform and policy continuity and scale up.

1. Governance that is based on scientific evidence, coordination between subsystems of the health system and national and sub-national governments, regulation of private actors, and the institutionalization of resilience.
  • Strong collaborations among national, sub-national, and local governments are necessary to establish and institutionalize stewardship and decision-making channels. In addition, the fragmentation of health systems must be overcome to ensure efficient and effective governance before, during, and after shocks.
  • Scientific evidence must be at the centre of governance and decision-making. Transparency and accountability are essential to building trust and communicating decisions to the public, especially about public health measures.
  • Collaboration with and amongst private sector actors is necessary and should be guided by regulations and incentives to help achieve national health goals throughout different stages of the resilience cycle.
  • An institutionalized organizational structure is required, either as a standalone entity, or within a Ministry of Health or an independent emergency agency, with the mandate, autonomy and science-based approach to lead the management of public health emergencies, including support to PHC at the territorial level.
  • Emergency/Disaster Health Committees and Emergency Health Plans need to be developed with PHC representation and participation that considers its role in emergency preparedness and response.
  • Advisory councils/committees with broad participation of government sectors and levels, service providers, civil society and community-based organizations, who can be called upon at times of crisis to guide decision-making.
2. Primary care teams trained, sufficiently staffed, and adequately supported to face public health emergencies.
  • Primary care workforce and decision-makers at all levels are given opportunities to test and refine primary care plans and processes to respond to a potential shock, for example by holding simulation exercises or drills.
  • Teams trained and sufficiently staffed to prepare, respond and learn from shocks. Equipment and other materials needed during emergencies are easily available for PHC teams.
  • Health workforce education, training curricula and continuing education tailored to advance PHC resilience, including leadership and management skills, multi-professional teamwork, community health action, and culturally appropriate care and engagement in the context of emergencies.
  • Protective measures with appropriate personal protective equipment and social support during shocks (e.g., childcare, mental health) formally assured and effectively implemented.
3. Digital technologies, supply of essential medicines and vaccines, and infrastructure adequately developed to provide the necessary support in the event of shocks.
  • Digital health tools are critical to supporting many goals along the resilience cycle. Telemedicine and remote monitoring are critical to maintaining essential health services, broad-band internet connections and mobile devices are critical for crisis communication.
  • Supply chains that include primary care for essential medicines, vaccines, personal protective equipment and other supplies need to be assessed before emergencies so that basic health provisions can be guaranteed during shocks.
  • Physical infrastructure at the primary care and hospital level that allow services to remain functional during and after extreme events, as well as designed and operated to reduce resource consumption and minimize negative environmental impacts, providing equitable and accessible health care to all users.
4. Diagnosing, monitoring, and evaluation to ensure knowledge is generated for decision making across the resilience cycle.
  • Risk assessments of a wide range of potential risks for public health emergencies, which consider how each shock would impact PHC-based systems. These assessments are a starting point to explore strategies to mitigate and manage these risks as part of PHC-based system preparedness.
  • Data ecosystems and a skilled workforce that are able to provide real-time analysis, interpretation and knowledge translation for decision-making and informing policy. This includes epidemiological surveillance, administrative data systems, and other data sources.
  • Monitoring and evaluation strategies that assess processes and outcomes of policies and interventions for PHC resilience so lessons learned that can be used to strengthen PHC-based systems into the future. Robust monitoring and evaluation fill important research and implementation gaps, while also ensuring that investments are effective over time.
  • Post-crisis/recovery reviews and reporting processes to demonstrate accountability and transparency, ensure that response strengths and weaknesses are identified, analysed and to improve response strategies by fostering a learning systems and continuous quality improvement culture.
5. Regional and global cooperation and multilateralism are critical for PHC resilience and must be protected and strengthened.
  • Social solidarity and cooperation between countries is key to resilience and must be promoted in regional and global dialogues and diplomacy. No single country can control global crises and risks such as climate change and pandemics–even from a national security perspective, multilateralism is in the best interest of all countries.
  • Regional collaboration in LAC is required to ensure access to essential primary care supplies and vaccines during a crisis, such as production and/or purchasing of vaccines, personal protective equipment (PPE), and other relevant equipment and supplies, to address the profound inequalities between the global North and South.

## Introduction

### Universal health and PHC-based systems: ensuring the highest possible level of health and well-being

Over the past 50 years, the Latin America and the Caribbean (LAC) region has made considerable advancements in improving health systems and outcomes, with many countries achieving notable progress in expanding access to essential health services, reducing health disparities, and increasing life expectancy. For example, in 2019, 77% of the population in LAC had health service coverage compared to just 65% in 2000.^1^ Despite the far-reaching impact of the COVID-19 pandemic, life expectancy in LAC reached 75.1 years in 2021 and the average infant mortality rate has fallen 38% in the past 20 years (from 24.2 to 15 per 1000 live births).^2^ Starting in the 1990s, countries across LAC undertook health and social sector reforms grounded in principles of solidarity and collective action to overcome inequities.^3^^,^^4^ The region’s commitment to universal health and primary health care (PHC) since the 2000s has been instrumental in upholding these principles by fostering more inclusive, community-based health care models that are responsive to the diverse needs of the population.^5^^,^^6^

Indeed, universal health and PHC are essential for tackling the region’s unique health challenges and ensuring equitable health for all.^7^ Many LAC countries have championed universal health to provide better access to essential health services without financial hardship, including services for prevention, health promotion, treatment, rehabilitation, and palliative care.^8^ PHC-based systems play a key role in advancing universal health by offering a people-centred, community-based approach to health care (See Panel 2 for definitions). With a focus on prevention, early intervention, and continuity of care, PHC-based systems can provide responsive services and, through multi-sectoral action, address the social determinants of health, thus reducing health disparities that disproportionately affect Indigenous populations, LGBTIQ+ communities, migrants, and other marginalized groups in the region.^14^, ^15^, ^16^, ^17^

Panel 2 **Terminology used in the Commission.**

1. **Essential health services**: Health promotion, preventive services, diagnosis, treatment and rehabilitative and palliative services.^9^
2. **Essential public health functions**: The capacities of health authorities, at all institutional levels and together with civil society, to strengthen health systems and guarantee the full exercise of the right to health, acting on the risk factors and social determinants that have an effect on the health of the population.^10^
3. **Primary care:** Used when referring to service provision at the first level of care.
4. **PHC or PHC-based systems:** A whole-of-society approach that strengthens health systems and maximizes the level and distribution of health and well-being.^11^
5. **Resilience:** Integrated systems that are aware of threats; agile in response to evolving needs; absorptive of shocks; adaptive to minimize disruptions; and able to transform after a crisis based on lessons learned.^12^
6. **Universal health:** Encompasses universal access to health and universal health coverage.^13^

### The call for resilient health systems: an opportunity for strengthened PHC-based systems

Despite progress made, countries across LAC continue to fall short of achieving universal health and ensuring PHC-based systems that provide high quality care for all.^18^ The region is characterized by deep inequities and fragmented, underfunded health and social protection systems that present persistent barriers to equitable health access and quality, particularly for marginalized groups.^2^^,^^19^^,^^20^ At the same time, countries in LAC are increasingly exposed to natural disasters and waves of crises driven by pathogens, conflict and violence, political turmoil, and the effects of climate change.^21^ As well as more slow-burning crises driven by an increasing burden of non-communicable diseases and population ageing that strains health and social care systems.^22^ Together, these shocks and slow-burning crises place far-reaching demands on health systems and communities to be resilient.^23^ These demands loom large for PHC-based systems that too often are ill-equipped to effectively and equitably respond, while providing ongoing essential health services and maintaining essential public health functions (EPHF). Ensuring the health and well-being of people and populations in the face of shocks is not easy. Recent shocks emphasize that it is a complex and urgent challenge spanning policy, practice, and research.

One such recent shock was the COVID-19 pandemic, which had a devastating effect on lives and livelihoods across LAC. Health systems across the region were overwhelmed and had to adapt amidst uncertainty, political challenges and high demands.^24^, ^25^, ^26^ The COVID-19 pandemic further exposed vulnerabilities in health systems, particularly in their ability to provide continuous and equitable care during crisis. Across LAC, primary care was often underutilized or neglected during the response. Yet, where primary care was included in the response, the proximity and engagement with communities, innovations in care delivery, adaptations and outcomes reported emphasize the importance of PHC-based systems in supporting health and well-being in communities.^25^

Definitions of health system resilience remain debated, but key ideas include the capacity of health systems to prepare for, respond to, and recover from shocks while maintaining essential functions.^27^^,^^28^ Efforts to characterize, operationalize, and measure health system resilience in relation to health systems performance and broader health outcomes are ongoing, including work by WHO, PAHO, the Organisation for Economic Co-operation and Development (OECD), and the World Bank, amongst others.^12^, ^29^, ^30^ In LAC, collaborations are emerging to define and explore health system resilience from a regional perspective, and to apply the concept to health system performance and quality of care.^31^, ^32^ Despite these efforts, a gap persists in leveraging PHC for health system resilience. Urgent action is needed to describe the conceptual links between PHC and resilience and develop novel approaches to integrate resilience into PHC-based systems.

### About the Commission, report aims, objectives and structure

The World Bank-PAHO Lancet Regional Health Americas Commission on PHC and Resilience seeks to advance knowledge and inform decision-making for the future development of PHC-based systems across LAC.^33^ The Commission’s work is especially timely, given the region’s vulnerability to a range of public health emergencies such as those arising from climate change, pandemics, natural disasters, conflict and violence, and other shocks. These risks, compounded by existing health system weaknesses, fragmentation, and inequities, demand urgent action. Despite efforts to strengthen PHC in LAC, there is a pressing need for a comprehensive framework to guide the transformation of PHC-based systems to be more resilient to shocks while ensuring the continuity of essential health services and EPHF.

The Commission takes an innovative approach that systematically links PHC-based health systems to resilience, emphasizing that PHC is a strategy that builds broader health system resilience, while offering a crucial link between community and multi-sectoral capacities. By linking PHC, resilience, EPHF, and universal health, the Commission offers a novel and holistic perspective on the role of PHC in fostering health system resilience in the face of ongoing and emerging challenges. Importantly, there is no trade-off between the development of PHC-based systems and health systems resilience; in fact, they are synergistic. When PHC-based systems are strengthened, health systems resilience is bolstered, and vice versa.

The Commission aims to deliver robust, region-specific recommendations for strengthening PHC resilience across LAC. Countries from other regions could also use and adapt its recommendations to their own context. The work seeks to help countries invest in and transform their PHC-based systems, enhancing resilience to shocks through evidence-based, actionable policy and operational recommendations.

Specific objectives of the Commission include:

1. **Develop a comprehensive policy framework** for PHC and resilience that outlines key policy domains and the functions PHC must develop to strengthen health system resilience.
2. **Assess the potential consequences** of not building resilience in PHC-based systems, including the impact on population health, social outcomes, and economic stability. This will highlight the economic rationale for investing in resilient PHC systems.
3. **Identify pivotal strategies and policies** that LAC countries should pursue, including key PHC investments that can improve resilience to future shocks and mitigate their effects on health, equity, and the economy.

By fulfilling these three objectives, the Commission makes three key original contributions to scientific literature and policy dialogue. Firstly, it explicitly links the concepts of PHC and resilience, which until recently have mostly been analysed separately. Secondly, the Commission produces a policy framework and actionable policy options linking PHC and resilience for LAC, which could also be considered elsewhere. Thirdly, it models the cost to population health and the economies of inaction on PHC and resilience in LAC.

This is the first Lancet Commission under the Regional Health Americas umbrella and the second Lancet Commission to focus on PHC (following the Lancet Global Health Commission on financing PHC).^34^ Through this work, the Commission fills an important gap in global health literature and policymaking, offering timely and actionable insights for LAC countries facing a convergence of complex health and social challenges. Refer to Panel 3 for an overview of the methods and Supplementary Material 2 for complete methods.

Panel 3 **Methods used in the Commission.**

To identify areas for action, this Commission used a mixed-methods approach with iterative expert feedback, umbrella reviews, stakeholder consultations across LAC, surveys, and qualitative studies, including a deep dive into five LAC countries. By triangulating these methods, a suite of recommendations and corresponding policy options were identified to build resilient PHC-based systems in LAC drawing on integrated service delivery and EPHF, community engagement and participation, financing, and multi-sectoral action. Please refer to Supplementary Material 2 to see more details about these methods.

## Part I: shocks and the cost of inaction in PHC resilience on population health and the economy: why LAC, why now, and why is it urgent?

Countries in LAC are at high risk of disasters, public health emergencies, and humanitarian crises that consistently and increasingly challenge PHC resilience.^35^^,^^36^ As countries chart a course towards strengthening resilient PHC, it is important to consider the context shaping PHC-based systems in recent years. We offer a snapshot of the region by exploring three key areas that illustrate the case for addressing PHC resilience now and as an urgent matter in LAC. These include the high cost of inaction in strengthening PHC resilience on population health and the economy; the weak responses and high impact of past public health emergencies; and the high-risk profile of the shocks faced by the region in the future.

### The cost of inaction in PHC resilience in LAC

Overlapping shocks and resulting crises pose an accelerating and intersecting threat to the health and well-being of millions across the region, with knock-on effects for livelihoods and economies. In addition, the existing capacities and challenges outlined above point to systemic weaknesses that both hinder PHC resilience and demonstrate the dire need for action towards PHC resilience. As Wickramaarachchi and colleagues^37^ describe in an analysis prepared for this Commission, there is an immense cost to PHC-based systems that are unable to weather shocks.

**Fig. 1:**
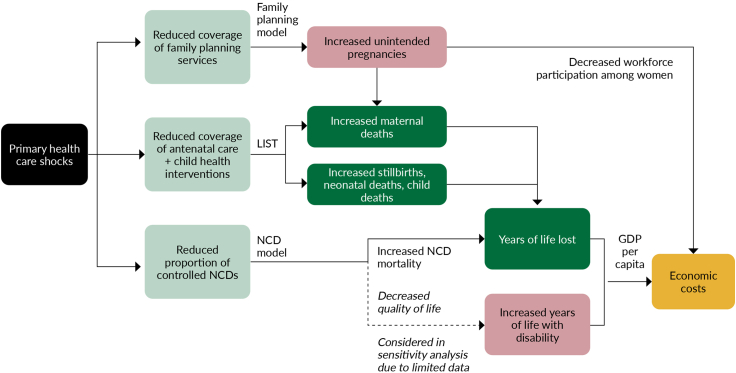
**Framework used to estimate the health and economic costs of shocks to primary care services.** Note: LiST: Lives Saved Tool; NCD: non-communicable diseases; GDP: Gross Domestic Product.

The effects of shocks to PHC-based systems lead to economic costs in a number of ways depending on magnitude of reduction of health service provision and their duration (Fig. 1). By causing reduced coverage of family planning services, antenatal and child health interventions, and reductions in NCD control, these shocks contribute to unintended pregnancies, increased maternal deaths, stillbirths, neonatal and child deaths, leading to decreased workforce participation, years of life lost, increased years of life with disability, and ultimately economic costs. While these are examples of some key PHC services that are considered in the Commission’s framework and analysis, there are numerous other services, for example immunization, oral health and cancer care, amongst many others, which can be impacted by shocks, as was seen during the COVID-19 pandemic.^38^ The examples chosen had adequate data availability and were able to be modelled effectively, however, the above is likely an underestimation of the cost of inaction.

**Fig. 2:**
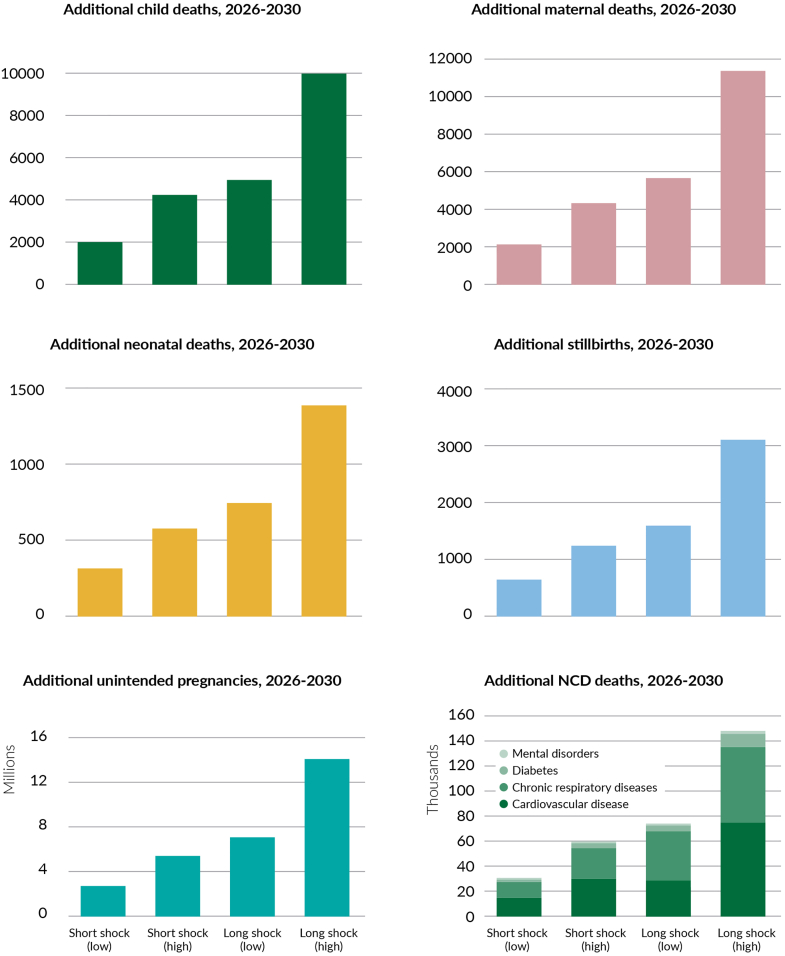
**Cumulative additional adverse health outcomes for each scenario-based shock to PHC-based systems.** Note: Scenarios used in the model: **Strengthened PHC:** Assuming investment occurs to strengthen PHC, such that there are no changes to PHC intervention coverages when the health system shock occurs; **Short PHC shock (low/high)**: PHC intervention coverage in 2026 assumed to reduce by (a) 25% or (b) 50%, before returning to baseline levels in 2027 and maintained through to 2030; **Long PHC shock (low/high)**: PHC intervention coverage in 2026 assumed to reduce by (a) 25% or (b) 50%, before linearly increasing to reach baseline levels again by 2030. For each country, baseline coverages were estimated based on what is currently being achieved.

Without investment in resilient PHC the cost of inaction is very large, with far reaching impacts on health and well-being. Experience from the COVID-19 pandemic showed estimates of service reduction that could range between 20 and 50% depending on the service and country, with reductions lasting at least two years.^38^ Our model illustrates that, depending on scenarios, shocks to PHC-based systems in LAC that lead to a 25–50% relative reduction in primary care coverage against what’s currently covered in each country with a one to five year recovery period could result in societal economic costs ranging from US$ 7 billion to US$37 billion per shock. Brazil, Mexico, Venezuela, and Argentina would bear the brunt of these costs, with the highest economic impact as a percentage of GDP in Haiti and Honduras. These shocks have profound impacts on health and well-being, with conservative modelling estimating that each shock could result in an estimated 600–3100 stillbirths; 300–1400 neonatal deaths, 2000–10,000 child deaths, 2200–11,300 maternal deaths, 29,000–149,000 NCD deaths, and 2.7 million—14.1 million unintended pregnancies (Fig. 2).

**Fig. 3:**
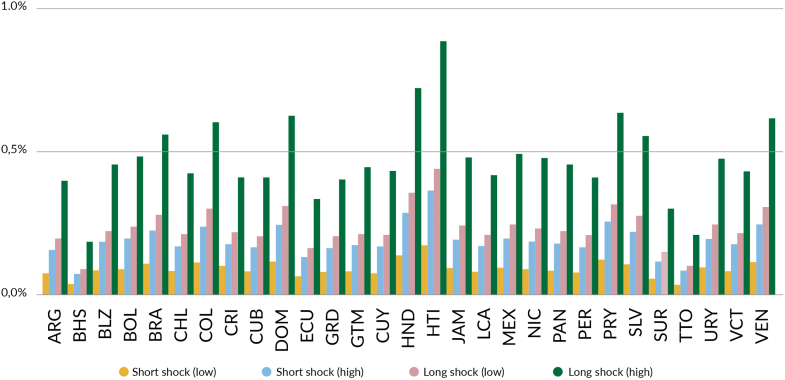
**Estimated economic cost of shocks to PHC-based systems as a percentage of the national GDP in 2023**.

The duration of these shocks influences their impact (Fig. 3). Each short-term shocks (one year duration) with a 25–50% reduction in primary care coverage could result in additional societal economic costs of US$ 7.2–14.8 billion. Each long-term shocks (five years duration) with the same reduction could escalate costs to US$ 18.4–37.0 billion. As a proportion of GDP, the societal cost of each shock scenario is estimated to range from 0.03 to 0.18% in the short and low magnitude scenario to 0.12–0.92% in the long and high magnitude scenario.^37^ Investing in PHC resilience is essential to mitigate these potential impacts, ensuring better preparedness and response to future shocks, and protecting lives and economic stability in the region.

While the above figures clearly demonstrate the immense cost of not having resilient PHC, they are in fact conservative estimates, with limitations related to data availability (e.g., not all countries had all service domains considered in the analysis, and cancer data were not included in the models), and that only represent a fraction of the total cost in the event of shocks hitting a non-resilient PHC-based system. Further, our analysis focuses on a single shock, whereas in reality multiple shocks are likely to occur over time. Indeed, a World Bank report on the health impacts of climate change alone, estimated a cumulative impact in LAC of 0.19% and 0.45% of GDP between 2026 and 2050.^39^ Finally, our analysis uses national estimates, suggesting that certain vulnerable areas or populations, where baseline coverage is lower or recovery time longer, could be hit much more severely.

### Past public health emergencies have impacted LAC countries more than other regions

Countries across LAC have faced, and will continue to face, complex crises that multiply risks to health and well-being in communities, and strain PHC-based systems.^21^^,^^35^ The COVID-19 pandemic and responses to climate change in LAC are key examples of PHC responses and challenges for resilience.

**Fig. 4:**
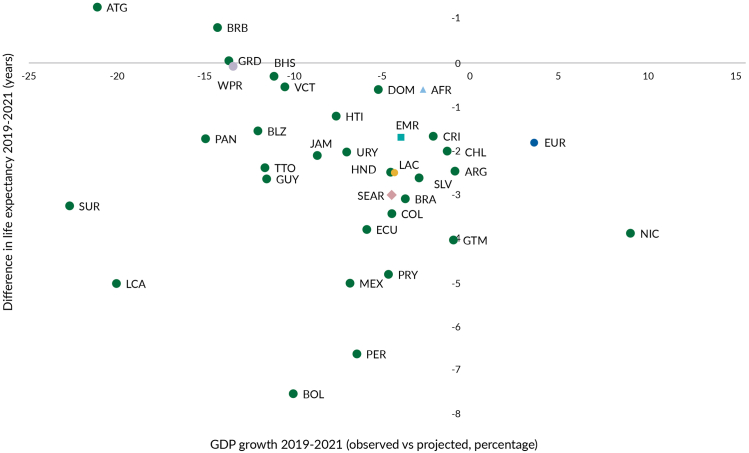
**COVID-19 life expectancy reduction (2019–2021) and GDP growth 2019–2021 (observed vs. projected).** Source: World Health Organization Global Health Observatory, 2025^41^; International Monetary Fund, World Economic Outlook Database, 2025.^42^ Note: The Y axis shows the difference in life expectancy at birth (measured as years) comparing 2021 with 2019. Negative values entail a reduction in life expectancy during this time period. The X axis shows the difference between the observed and the projected gross domestic product growth of the period 2019–2021. Hence, negative values entail that countries GDP’s actual growth was less than the expected growth in the same time period.

Despite being underutilized, some countries in the region leveraged PHC during the COVID-19 response to bolster health system resilience. Activities grounded in a PHC approach included communicating risks in communities, supporting testing, surveillance, and vaccination efforts, while adapting and innovating to provide routine essential health services.^40^ Yet, longstanding vulnerability and underinvestment meant health systems could not meet the demands of the pandemic, with notable declines in health care seeking and use in the region. For example, LAC accounts for 8.5% of the world’s population but reported approximately 13% of global COVID-19 cases and 30% of total deaths from COVID-19 as of 2022.^21^^,^^25^ Dramatic increases in mortality rates and reduced life expectancy were seen, with the highest drop between 2019 and 2021 compared to other regions (Fig. 4). Notably, Bolivia, Peru, Saint Lucia, and Mexico experienced a reduction of five or more years in life expectancy, double the LAC average of 2.5 years reduction in life expectancy.^40^

The COVID-19 pandemic also caused underlying vulnerabilities and inequities to be laid bare, bringing more groups into precarity and poverty that profoundly impact their health and well-being. For example, the economic impact of the pandemic was severe and protracted as GDP across the region contracted by 7% in 2020, the worst globally, pushing millions into poverty and reversing years of progress in poverty reduction across the region.^25^^,^^43^ In terms of the GDP growth projected in 2019 and the observed in 2021, the contraction in LAC was only behind Western Pacific and close to South-East Asia, with Panama, Bolivia and many Caribbean countries seeing the largest reductions (Fig. 4). These economic impacts in turn created new, and augmented existing barriers to primary care access, particularly for marginalized communities in LAC.^25^

**Fig. 5:**
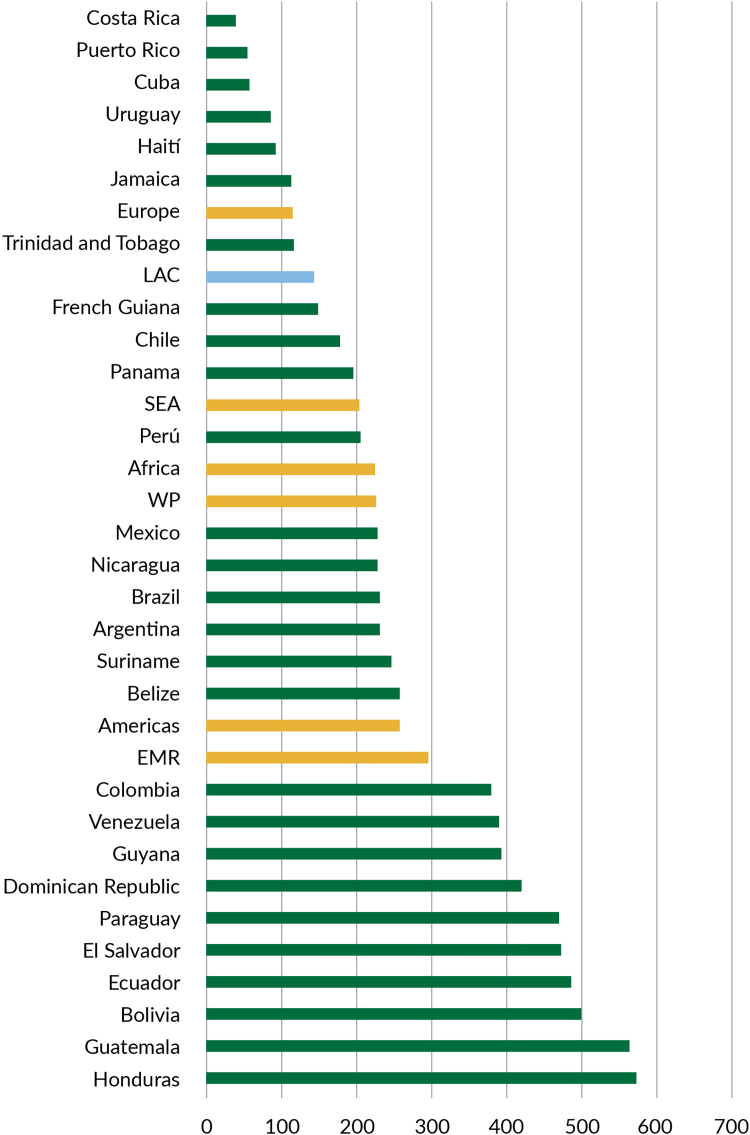
**Percentage change in the number of average annual heat-related deaths in 2020**–**2023 compared to 1990–1999 period due to climate and population changes.** Source: The 2024 report of the Lancet Countdown on health and climate change: facing record-breaking threats from delayed action. Lancet Countdown 2024. LAC: Latin America and the Caribbean; SEA: South East Asia; WP: Western Pacific; EMR: Eastern Mediterranean Region.

Shocks such as COVID-19 unfold against a backdrop of rapidly increasing climate change, with far-reaching impacts—both direct and indirect—across the region.^44^ Primary care is the first point of care for the millions of people at risk or affected when faced with extreme weather events, such as heat waves, hurricanes, floods, and wildfires, to name a few. Take heat waves, which have resulted in a 144% increase in the number of average annual heat-related deaths of adults older than 65 years between 1990–99 and 2020–23, 13 percentage points higher than the 131% that would have been expected without rising temperatures (Fig. 5).^45^ In addition to threatening health and well-being, climate-related events have been identified as amplifying health service delivery challenges in the region.^35^ For example, many across the region depend on navigating waterways to access care, which becomes limited during droughts. In response to the increasingly complex and overlapping climate events in LAC, there are growing calls for primary care to be a platform for adaptation to climate change in the region.^46^

Beyond the direct effects of extreme climate events, PHC-based systems in LAC must at the same time be resilient to respond to shifting disease exposures and risks at the human-animal-environment interface shaped by shifting environmental factors. These contribute to public health emergencies that recur or persist over longer time frames and place a strain on health systems. For example, the range expansion of *Aedes* mosquitos has contributed to notable and growing outbreaks of arboviruses across LAC in recent years.^47^ Dengue continues to impact health and well-being, while also placing an economic burden on the region. The average annual cost of dengue exceeds US$ 3 billion, including direct medical costs for people requiring hospitalization and indirect costs through lost productivity.^48^ Effectively identifying, preventing, and responding to arbovirus disease outbreaks requires resilient PHC that is engaged with public health efforts for sustained surveillance, community-engaged prevention and control approaches, and timely medical care.^49^, ^50^, ^51^ As new and expanding vector-borne outbreaks and extreme events are predicted to increase in LAC, the development of PHC-based systems and health system resilience go hand in hand. This means that there is no trade-off between them but are mutually reinforcing.

### Characterizing the high-risk profile of shocks in LAC

Countries in LAC are particularly exposed to shocks. The United Nations Office for the Coordination of Humanitarian Affairs (OCHA) classifies LAC as second in disaster-prone regions worldwide, with more than 190 million individuals affected by more than 1500 disasters from 2000 to 2022.^52^ The consequences of these disasters include injuries, fatalities, increase in infectious diseases, and long-term adverse mental and physical health outcomes.^53^ Additionally, these disasters directly threaten and impede the capacity of PHC-based systems to deliver essential services and to respond to the increased demand caused by the disasters. To ensure resilient PHC, there is urgent need to consider and prepare for the variety of shocks, crises, and their impacts in the region (Panel 4).

Panel 4 **A consideration of shocks, crises and their impacts.**

Two typologies of shocks have been used to examine types of crises that unfold after a shock.^54^, ^55^, ^56^ The first considered health care resilience and trust in the government. In this case, it is hypothesized that shocks can be mitigated with minimum losses when both resilience and trust are strong, and an uncontrolled crisis could emerge when both are weak. When only one of these factors is strong, it is unclear if and how a crisis will unfold. The second considered health care resilience and predictability of the shock based on historical records. In this case, if the shock is not anticipated, a catastrophe may ensue if healthcare systems lack resilience. If the shock is expected, however, resilience will enable mitigating the potential effects, while lack of resilience will result in excess damage.^57^

The impact of shocks in health care may include a range of consequences, for example the destruction of infrastructure (e.g., buildings, equipment, connectivity), workforce shortages, workforce stress and burnout (particularly among primary healthcare workers in the aftermath of shocks^58^), disruption of health services, reduced access to healthcare services, increased demand for services (often beyond capacity and exacerbated by population displacement), decreased quality, and financial burden.^59^, ^60^, ^61^ The nature and magnitude of these consequences depend on the type and severity of the shock, the speed at which it occurs and concludes, the level of population exposure to the shock, the vulnerability of those exposed, and the institutional and infrastructure mechanisms in place that contribute to coping with the shock.

**Fig. 6:**
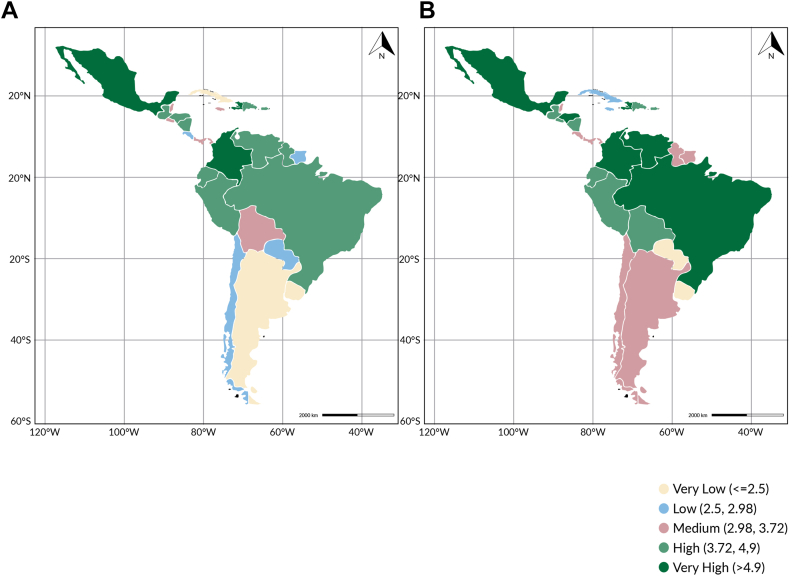
**LAC-INFORM risk index by country (A) 2015; (B) 2024.** Source: LAC-INFORM.^62^

Castro and colleagues, in an analysis prepared for this Commission, have classified shocks into three categories: natural, anthropogenic, and climate change-related (Fig. 6).^54^ All shocks classified as climate change-related may result from extreme events and changes in weather patterns due to human-induced greenhouse gas emissions. It is noteworthy that countries may be subject to multiple and sequential shocks. For example, hurricanes have the potential to result in flooding, which can in turn lead to population displacement.

Critical in the effort to prepare for a shock is the ability to assess the risk that shocks may occur and result in major disasters and humanitarian crises. In this context, risk is defined as the interaction between hazard, exposure, and vulnerability.^63^ The LAC-INFORM Risk Index,^64^ produced by the Inter-Agency Standing Committee Reference Group on Risk, Early Warning and Preparedness and the European Commission, provides a comprehensive risk assessment for LAC countries. It measures the risk of humanitarian crises and disasters considering several dimensions, namely natural hazards (earthquake, tsunami, river flood, coastal flood, tropical cyclone, drought, and epidemics), human hazards (conflict intensity and projected conflict probability), socioeconomic vulnerability (poverty, inequality, aid dependency), vulnerable groups (uprooted people, and other vulnerable groups), lack of institutional coping capacity (disaster risk reduction, and governance), and lack of infrastructure coping capacity (communication, physical infrastructure, and access to health care and education).

**Fig. 7:**
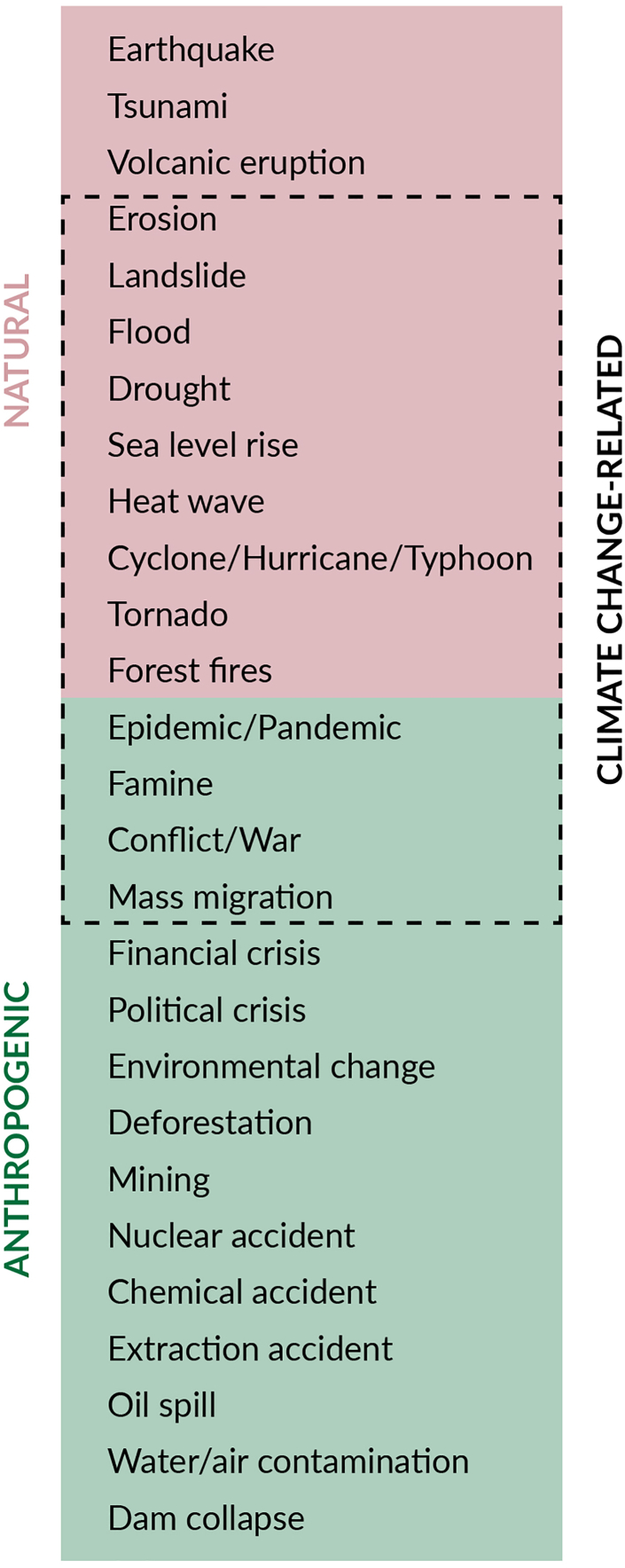
**Categories of types of shocks**.

According to the LAC-INFORM Risk Index calculated for the period from 2015 to 2024, Haiti has been the country with the highest risk.^54^ But the risk has increased over time for several countries (Fig. 7), mainly due to more frequent and severe natural hazards, likely a result of human-induced greenhouse gas emissions.^55^ By providing scores in each of the risk dimensions, LAC-INFORM highlights the main weaknesses in each country. For example, although Chile and Suriname have the same risk index, the most important dimension in Chile is hazard and exposure, while in Suriname it is the lack of coping capacity. Of note is the fact that hazard, exposure, vulnerability and lack of coping capacity vary between countries but also within countries. Therefore, although LAC-INFORM is a major step toward risk assessment in LAC, it is not the ideal tool for countries to develop preparedness and response plans at the local level. Subnational risk assessments are needed to support those plans. Otherwise, avoidable injuries, deaths, and disruptions in primary care will continue to happen following shocks, with significant social and economic costs.

Many examples highlight the negative consequences of shocks for primary care in LAC.^54^ For example, earthquakes resulted in limited and delayed treatment in Chile,^56^ and catastrophic losses in Haiti.^58^, ^59^, ^60^ Droughts, often associated with population displacement,^61^^,^^65^ have intensified over the last decade. In 2024, under the effect of a severe El Niño, the Brazilian Amazon experienced an unprecedented drought, which left numerous Indigenous and riverine communities without access to health and other essential services.^66^ The COVID-19 pandemic exposed the unpreparedness of countries to manage a major public health emergency. Considering the excess deaths in 2020 and 2021, relative to the number of expected deaths, the top four countries are from LAC (Peru, Ecuador, Bolivia, and Mexico), and three other LAC countries are among the top 20 (Colombia, Paraguay, and Brazil).^67^

In contrast, a few examples demonstrate the importance of preparing and planning for shocks.^54^ In 2021, the La Soufrière volcano erupted in St. Vincent and the Grenadines. In anticipation, the government evacuated those living near the volcano, moved health services to safer areas, provided medical resources and personnel, and deployed public health messages, no deaths were reported.^68^ Also, during the COVID-19 pandemic, Costa Rica took action to guarantee that essential health services were maintained,^69^ and implemented actions that facilitated pandemic response and contributed to low mortality.^70^

Primary care plays a pivotal role during emergencies following shocks, deploying services beyond its routine activities. Therefore, preparing long before the shock, being ready to respond during and immediately after the shock, and having a recovery plan post shock is critical to minimize injuries and loss of lives through resilient PHC that contribute to better coping capacity with shocks.

## Part II: systemic challenges in the quest for resilient PHC in LAC

### PHC organization features in LAC countries

LAC is a region marked by diversity, where PHC has evolved and produced distinct models of care across countries. Despite wide variation, most countries have some level of territorial catchment area for each health facility, but formal empanelment exists in few countries. Only in 4 of 33 countries patients are obliged to register in a PHC facility or practice to receive services (Brazil, Chile, Cuba and Suriname), while in 16 there is no incentive and no obligation to register with a primary care physician or practice and in 6 countries patients are encouraged to register but it is not obligatory. Regarding the gatekeeper function, in 19 of 33 countries a primary care physician referral is compulsory to access most types of specialist care in the public or social insurance sector, while in 8 countries there is no need and no incentive to obtain primary care physician referral. In the rest of countries there is no clear referral policy in place or no formal gatekeeping structure.

In terms of human resources, the workforce density is asymmetrical across countries with an average of 61.9 health workers per 10,000 population, and 14.9 per 10,000 population for community health workers.^71^ Thirty-four percent of the countries commonly have their PHC teams composed by physicians, nurses and technical assistants, while 56% of them have, in addition to the latter, community health workers.^71^ A similar variation is seen on PHC expenditure with a wide variation across countries (average of US$ 320 per capita (median US$ 272)), but only 10 LAC countries are able to estimate and report this information.^72^

### Underlying challenges faced by PHC-based systems in LAC that hinder resilience

The persistent structural challenges facing PHC-based systems in LAC can be understood through the lens of the Commission’s framework for resilient PHC-based systems, as outlined in Part III of this report (refer to Fig. 12). The Commission’s framework highlights that building resilience is not limited to the capacity to respond to shocks but requires sustained action to address the underlying weaknesses that constrain the performance of PHC across all phases of the resilience cycle: prevention and preparedness, shock and alert, responsiveness and impact, and recovery and learning. Moreover, the Commission’s framework emphasizes that resilient PHC depends on coordinated progress across four interdependent pillars: Integrated health services and essential public health functions (EPHF), Community empowerment and participation, Multisectoral action, and Financing.

The structural barriers described in this section, including gaps in coverage, persistent inequities, system fragmentation, health workforce shortages, and underdeveloped EPHF capacities, directly weaken these four pillars and, by extension, undermine the resilience of PHC-based systems. These challenges not only limit the ability of countries to anticipate, absorb, adapt to, and recover from shocks but also prevent the realization of the Commission’s vision for resilient, equitable, and people-centred PHC in the region.

#### Limitations to achieving integrated health services

Despite progress towards strengthened PHC-based systems across the region, advancing resilient PHC continues to be challenged by the pervasive and structural health system challenges in the region. These include gaps in service coverage, high unmet health care needs and barriers to access, high out-of-pocket payments (OOP), inequities shaping access to primary care, health workforce deficits, and system fragmentation.^25^

**Fig. 8:**
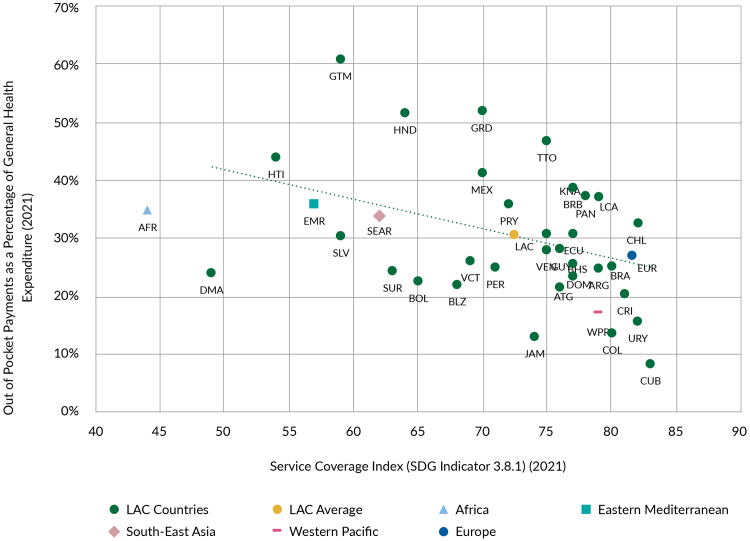
**Out of pocket payments in relation to service coverage index 2021.** Source: WHO Global Health Expenditure Database and Global Health Observatory, 2025.

Countries in the region have made strides towards more complete universal health with gains in service coverage and financial protection. Yet, ensuring service coverage for all remains a challenge and many individuals, particularly the millions of people informally employed, still face substantial OOP expenses.^73^ In 2021, the service coverage index in LAC lagged behind Europe and the Western Pacific, but countries such as Dominica and Haiti were closer to African countries, while Cuba, Uruguay, Chile, and Costa Rica were above the European average, highlighting the large disparities among LAC countries. OOP spending accounted for 30% of current health spending in LAC, ranging from 61% in Honduras to 8% in Cuba (Fig. 8). High OOP spending limits people’s ability to afford necessary care, including primary care.^6^ Although most LAC countries reduced OOP health expenditures between 2010 and 2019, approximately 1.7% of the population fell into poverty due to health care costs, with 12.7% of those already in poverty pushed further below the poverty line.^2^ Notably, Fig. 8 also shows how a higher service coverage index is associated with lower OOP, highlighting how the two goals might be closely linked.

In addition, the quality of PHC services is central. Ambulatory care-sensitive conditions (ACSCs), which are widely used as a measure of performance, show that gaps remain. A study conducted in eight Latin American countries found that ACSCs accounted for an average of 17.4% of discharges and days of stay in public sector hospitals, with limited variation between countries but significant subnational disparities that, if reduced, could significantly reduce ACSC discharges and days of stay.^74^ In the context of public health emergencies, the challenge is not only to maintain services, but also to ensure a minimum level of quality.

#### The missed opportunity of further introducing EPHF in PHC

Essential public health functions provide a comprehensive approach to public health, ensuring LAC countries are better prepared to respond to and recover from emergencies while maintaining essential health services (Panel 5). Strengthening EPHFs is therefore a fundamental component of building PHC resilience across LAC, as emphasized in the Commission’s resilience framework presented in Part III of this report (Fig. 12).

Panel 5 **About the Essential Public Health Functions (EPHFs).**

1. EPHFs are critical capacities to both preparing and responding to risks to health and wellbeing.
2. EPHFs include an array of activities and capacities that are based in ethical principles and seek to address health inequities and their causes and the social economic, cultural, and political conditions that determine the health of populations among other things.^10^^,^^75^^,^^76^
3. EPHFs offer an integrated approach spanning assessment, policy development, resource allocation, and access to strengthen health systems and ensure a full scope of public health.^10^^,^^77^
4. The importance of PHC-based systems to the effective delivery of EPHFs has become increasingly evident, as has their role in public health emergency response. EPHFs serve as a tool to enhance PHC-based systems by supporting the planning and incorporation of public health services into primary care.
5. By strengthening health systems, addressing inequities, and implementing financial protections, EPHFs provide a comprehensive approach to public health, ensuring LAC countries are better prepared to respond to and recover from emergencies while maintaining essential health services.

**Fig. 9:**
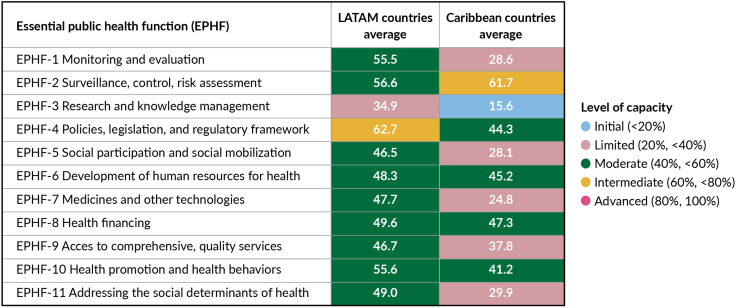
**Average level of EPHF capacity in LATAM and Caribbean countries.** Source: Adapted from Houghton et al. (forthcoming). Note: LATAM countries—Bolivia (Plurinational State of), Colombia, Costa Rica, El Salvador, Panama, Peru, and Honduras. Caribbean countries—Antigua and Barbuda, Bahamas, Belize, the Dominican Republic, Jamaica, Saint Kitts and Nevis, Saint Lucia, Saint Vincent and the Grenadines, Suriname, and Trinidad and Tobago.

Capacities to execute EPHFs are underdeveloped across LAC (Fig. 9). For example, based on recent data from the Pan American Health Organization, no EPHF scored an ‘advanced’ level of existing capacity.^78^ Latin American countries (note in Fig. 9), on average scored moderate level of capacity, except for those EPHF related to development of policies, legislation, and regulatory frameworks (intermediate capacity); and lesser development of research and knowledge management (limited capacity). Average development of EPHF in Caribbean countries (note in Fig. 9) highlights intra-regional differences. Of the 11 EPHFs, five scored as limited and one (research and knowledge management) was characterized as initial level of capacity.

When viewed through the lens of the policy cycle (refer to Table 1), there are gaps in the implementation of EPHFs across all stages. These gaps ultimately hinder progress towards PHC resilience given that many of the EPHF can take place in primary care and function to ensure that primary care is able to prepare for, respond to, and recover from shocks. At the same time, if primary care has deficits, a country will be unable to deliver on the EPHF. Complementarily, it is also important to reduce population vulnerability to shocks by increasing coverage and quality of health care and population health services.

**Table 1:** Summarized gaps in Essential Public Health Functions (EPHF) by policy cycle stage.

Policy Cycle Stage	EPHF	Gap
Assessment	1. Monitoring and evaluation 2. Surveillance, control, and risk management 3. Research and knowledge management	1. Issues with legal frameworks for monitoring and evaluation of systems, data collection, and information quality. 2. Weaknesses in risk communication, emergency response coordination, and resource availability. 3. Gaps in national health research agendas, training, and dissemination of research findings.
Policy Development	4. Policies, legislation, and regulatory frameworks 5. Social participation and social mobilization	4. Challenges in strategic health planning, resource allocation, and compliance assessment. 5. Lack of structures for social participation and empowerment of civil society.
Resource Allocation	6. Development of human resources for health 7. Medicines and other health technologies 8. Health financing	6. Issues with health human resource training, management, and legal frameworks. 7. Gaps in policies for the rational use of medicines and other technologies. 8. Need for clear policies on pooled financing systems and equitable resource allocation.
Access	9. Access to comprehensive, quality services 10. Health promotion and health behaviours 11. Addressing the social determinants of health	9. Challenges in service quality monitoring and health services network management. 10. Wide range of gaps in health promotion and risk reduction. 11. Need for policies addressing social determinants of health and health equity.

#### Barriers to community empowerment and participation

As of 2024, LAC is the world’s most unequal region—the 10% highest earners make on average 12 times more than the poorest 10%.^79^ Differences in access to and quality of primary care across the region are shaped by geography, employment, gender, race, ethnicity, age, education, amongst others.^1^^,^^80^^,^^81^ About two-thirds of the population face unmet health care needs, with disparities across and within countries in the region. Organizational barriers, such as long waiting times, financial barriers and acceptability issues, such as lack of trust in health care, are often reported as challenges. Inequities ultimately place limits on how resilient PHC-based systems can be by creating and amplifying barriers to accessing care in communities before, during, and after shocks. For example, Afro-descendants in Latin America face immense inequities that profoundly shape their health and well-being.^82^^,^^83^ The region is also home to 50 million Indigenous people belonging to more than 500 different ethnic groups.^25^ Indigenous people in the region face distinct cultural and linguistic barriers that limit their access to primary care, which despite efforts to address their needs, were similarly exacerbated during the COVID-19 pandemic.^84^, ^85^, ^86^ Another example are the systemic barriers LGBTIQ+ people across the region face when accessing health care including discrimination, stigma, and a lack of provider training on their experiences, which limit access to essential primary care services and contribute to inequities in care.^87^, ^88^, ^89^ These barriers to primary care were amplified during recent shocks such as COVID-19, resulting in further marginalization of LGBTIQ+ during crisis.^90^ Although there is ongoing work to actively create more inclusive primary care services in LAC, care in the region is often not designed to accommodate the diverse genders, languages, traditions, and cultural perspectives on health and healing, that are key elements of resilient PHC.^91^^,^^92^ (more on Panel 6).

Panel 6 **Breaking barriers: enhancing primary care for LGBTIQ+ people in Latin America.**

Across Latin America, many sexually and gender diverse (LGBTIQ+) people continue to lack access to basic health services and universal health. These communities face higher disease burdens, discrimination in health care settings, and heightened vulnerability during public health emergencies. Against this backdrop, Feune de Colombi et al., explored the barriers and enablers experienced by LGBTIQ+ people when accessing primary care.^87^ The work draws on qualitative focus groups that gathered insights from civil society organizations (CSO) and government officials across five countries with diverse socio-economic contexts.

Barriers to care for LGBTIQ+ people across Latin America are diverse and deeply rooted. The entrenched gender binary paradigm that pervades the region remains a significant obstacle across its fragmented health care systems. Discrimination, stigma and the absence of strong support networks for LGBTIQ+ people lead to increased psycho-emotional distress, which contributes to care-seeking avoidance and exacerbates existing inequities.

To ensure LGBTIQ+ people have equitable access to the care they need, when they need, political commitment, institutional validation, and inclusive legal frameworks are necessary to making sure LGBTIQ+ individuals can safely seek and access primary care services, and also create foundational support for creation and sustainability of LGBTIQ+ -led initiatives. In the study, telehealth and digital innovations, such as geo-referencing apps for LGBTIQ+ friendly health centres, were identified as essential strategies for ensuring access and extending the reach of community-level initiatives across LAC.

Feune de Colombi et al. also argue that fundamental reforms are needed. Policymakers and health care providers must develop inclusive, people-centred PHC-based systems that address the unique vulnerabilities and discrimination faced by LGBTIQ+ people, especially during public health emergencies. CSOs play an important role as intermediaries, bridging service delivery gaps, making their involvement in health policy dialogue essential. Importantly, policies and interventions must be grounded in multi-sectoral approaches that emphasize the social determinants of health and intersectionality that shape LGTBI + people’s health and well-being, as well as their ability to seek, access, and maintain care. In addition, without robust monitoring and evaluation, health systems will remain unable to provide high-quality care for LGTBI + people across the region. Real-time accurate and inclusive data collection ensures that the unique needs of LGBTIQ+ people are captured and addressed, leading to more tailored health care interventions. Finally, effective communication during public health emergencies and empowering LGBTIQ+ people to adopt healthy behaviours is very relevant. Including CSOs in the stakeholders pool ensures that communication is inclusive, credible, and reflective of their lived experiences. This partnership enhances preparedness and community mobilization during crises.

In parallel, the *2024 Regional Assessment Report on Disaster Risk in Latin America and the Caribbean (RAR24)* underscores that community participation, an essential pillar of equitable and resilient systems, remains unevenly implemented across the region.^93^ While many national policies reference the importance of engaging local populations and civil society, institutional frameworks often lack clear, sustained mechanisms for inclusive participation. Fragmented governance and compartmentalized policies across disaster risk reduction, climate, and development agendas further limit the coherence and effectiveness of community engagement. Marginalized groups, including Indigenous peoples, Afro-descendants, and residents of informal settlements, face structural barriers that restrict their ability to shape the design and governance of services that directly affect their well-being. Although community-led initiatives exist and reflect strong capacities for local mobilization, they are rarely integrated into formal systems or adequately resourced. Strengthening community empowerment thus requires moving beyond symbolic consultation toward institutionalized, transparent, and well-financed participation mechanisms that recognize the vital role of diverse community perspectives in building resilient and inclusive primary care systems.

#### Obstacles to multisectoral action

Health systems across LAC are characterized by their fragmentation, with health care services divided among multiple systems, including public and private providers, as well as different insurance schemes corresponding to socioeconomically stratified groups of the population, with varying levels of access and quality that influence access to primary care.^25^ These segmented systems perpetuate differences among population groups receiving health care, exacerbating inefficiencies, exclusion, and inequalities.^57^^,^^94^^,^^95^ Fragmentation also hinders care coordination, as patients navigate multiple providers without oversight, leading to long wait times and gaps in treatment, while receiving fewer services, and those that are received may be of poor quality.^96^ Additionally, health systems in most LAC countries lack effective data-sharing mechanisms between different sub-systems and service providers, leading to limited continuity as patients move between primary care and other levels of care.^25^^,^^12^ Cross-border health care coordination also remains a challenge, especially for migrant populations who may need care in multiple countries, while health records are often not shared between systems.^97^ Efforts to improve coordination are ongoing, but significant challenges remain to ensure that people across LAC can rely on integrated and coordinated systems as they face increasingly frequent and challenging shocks.

In addition to these internal challenges within the health sector, persistent obstacles to multisectoral coordination further undermine efforts to build resilient and integrated systems across LAC. The *RAR24* underscores that institutional silos remain deeply rooted, limiting collaboration between critical sectors such as health, education, environment, infrastructure, and social protection. This lack of coordination results in fragmented planning processes, duplication of efforts, inefficient resource allocation, and missed opportunities to address the social and environmental determinants of health. Although some countries have created formal platforms for multisectoral governance, these often lack sufficient funding, clear mandates, or political backing to operate effectively. The disconnect between disaster risk management and health system planning is particularly acute, weakening the region’s ability to anticipate and respond to complex and overlapping crises. Strengthening multisectoral governance is therefore essential to ensure that diverse stakeholders can work together to reduce vulnerabilities, address health inequities, and protect populations before, during, and after shocks.

#### Gaps in financing and health workforce development

Persistent gaps in sustainable financing and health workforce development continue to undermine the resilience of PHC-based systems across LAC. As previously highlighted, countries in the region have made strides towards more complete universal health with gains in service coverage and financial protection. Yet, ensuring service coverage for all remains a challenge, particularly for the millions of individuals working in informal employment who still face substantial OOP expenses.^63^

**Fig. 10:**
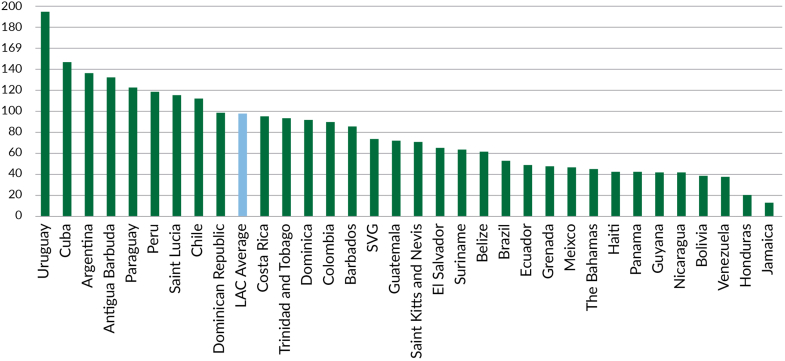
**Medical doctors, nurses, midwifes and community health workers–Total per 10,000 population, 2022.** Source: Authors based on Global Health Observatory data repository.^41^

However, financial protection alone is not enough to guarantee access to quality PHC services, particularly in underserved areas. Persistent shortages and the unequal distribution of trained health care professionals further compound these financing challenges and place significant limitations on PHC resilience (Fig. 10).

On average, LAC has two physicians per 1000 people, with only Cuba, Uruguay, Trinidad and Tobago, and Argentina exceeding the OECD average of 3.5 physicians per 1000 people. The region also lags in nursing staff, with just 3.6 nurses per 1000 people, significantly lower than the OECD average of 10.3 nurses per 1000 people.^2^ The health care workforce is also unevenly distributed, with most professionals concentrated in urban centres, leaving rural and remote communities underserved by primary care providers. Programs such as Brazil’s *Mais Médicos* and the Rural Practitioner Program in Chile have sought to address this issue by incentivizing doctors to work in underserved areas, with good results in terms of expanding access, but long-term retention of health care workers remains a challenge in the region.^98^^,^^99^ Training and educating an adequate number of skilled health professionals to meet the growing primary care demands in LAC is also an ambitious undertaking. While many countries in the region have invested in expanding medical and nursing schools, the number of health care professionals being trained, particularly for primary care and in rural and remote communities, is still insufficient to meet the growing and increasingly complex care needs in the region and to ensure PHC resilience.^100^ In addition, the migration of healthcare workers is another relevant issue. For example, the migration of nurses to high-income countries has significantly contributed to a greater deficit in the Caribbean.^101^

### Political economy as a central barrier for PHC resilience in LAC

PHC has long been recognized as the foundation of equitable and effective health systems.^33^^,^^102^, ^103^, ^104^ Despite broad consensus and repeated international commitments, from the 1978 Alma-Ata Declaration to the 2018 Astana Declaration, implementation in LAC has been inconsistent and often disappointing.^105^ While countries like Brazil, Chile, Costa Rica, and Uruguay have developed relatively robust PHC systems, many others have experienced fragmentation, reversals, and limited progress. This uneven performance is partly due to the factors presented and discussed in the previous section. However, it is also due to deeper political, institutional, and economic constraints that hinder sustained reform.

Applying a political economy lens, this section examines how interests, institutions, and ideas shape reform outcomes. The analysis draws on established frameworks, the theoretical insights of Acemoglu and Robinson and recent contributions by Bascolo and colleagues.^105^, ^106^, ^107^, ^108^, ^109^ It is organized into three dimensions: 1) Actors and interests, analysing the role of political coalitions, bureaucratic incentives, and professional resistance; 2) Institutions, focusing on governance arrangements, fiscal constraints, and legal frameworks; and 3) Ideas and discursive frames, exploring how narratives about PHC influence its prioritization and perceived legitimacy.

#### Interests: power dynamics and social mobilization

Interest groups across the political spectrum, including political parties, private sector actors, and professional associations, play a decisive role in shaping PHC policy. Political leaders often use health reform to gain electoral advantage, but subsequent changes in government may create policy instability.^110^^,^^111^ Electoral dynamics motivate governments to adopt PHC reforms to gain legitimacy or respond to public demands, but this also might result in volatility and discontinuity when administrations change. In Chile and Colombia, for example, electoral competition led to expanded access and equity initiatives, but their durability was compromised by regime turnover and lack of institutional anchoring.^110^

These structural weaknesses were further exposed during the COVID-19 pandemic, when some political leaders across Latin America often prioritized popularity over public health, downplaying the crisis to preserve popular support.^112^ Public health officials frequently encountered political resistance to evidence-based interventions, while conflicting pressures from civil society, the media, and private actors further complicated governance.^113^ In many countries, the entrenched institutional weaknesses and interests hindered coordinated, equitable, and science-based pandemic responses. The politicization of science, through attacks on experts, the silence of health authorities, or the co-optation of scientific narratives, emerged as a critical barrier to effective governance.^113^

On the private sector, some economic actors sought to reduce restrictions during the COVID-19 pandemic to protect legitimate financial and commercial interests.^112^^,^^114^^,^^115^ The pressures to ease restrictions reflect a broader political and fiscal dynamic in the region, where decision-making can be shaped by short-term incentives. During the pandemic, this was clearly illustrated by the trade-offs governments made between economic output and mortality rates. Rubinstein et al., 2023 demonstrate that in Argentina and Mexico, even marginal reductions in GDP loss would have resulted in substantial increases in daily COVID-19 deaths.^116^ In addition, the coordination between private and public health sector actors was hindered by the lack of transparency in procurement processes and the limited accountability, which further undermined trust and reinforced fragmentation in some health systems.^112^^,^^117^

Citizen interests have played a crucial role in shaping PHC adoption across Latin America, though their influence remains uneven. In Brazil, long-standing alliances between social movements and health professionals were instrumental in institutionalizing equity-oriented reforms under the Unified Health System (SUS). In Chile, popular mobilization contributed to the enactment of the Ricarte Soto Law, which expanded public coverage for high-cost diseases.^118^ However, community participation in PHC policy remains inconsistent and often fragmented. Patient organizations, where present, tend to focus narrowly on specific conditions like rare diseases, potentially limiting broader systemic engagement.^119^^,^^120^ During the COVID-19 pandemic, civil society involvement was further challenged by low trust in government, weak accountability mechanisms, and the lack of institutional spaces for community voice, especially in vaccine procurement and distribution, where opacity and elite capture were prevalent.^112^^,^^117^^,^^121^ Particularly regarding COVID-19 vaccines, Duryea and Pereira documented how community-based organizations in countries like Peru and Argentina actively sought to influence allocation processes, advocating for the inclusion of vulnerable groups such as women, Indigenous peoples, persons with disabilities, and LGBTIQ+ communities.^121^ However, their role was mostly consultative, with limited access to decision-making, reflecting entrenched power asymmetries. Moreover, in some countries, civil society actors were divided, some advocating for human rights and public health, others opposing measures like mask mandates or vaccination, often reflecting polarization and fragmented trust in scientific authorities.^114^

#### Institutions: fragmentation and governance gaps

Formal and informal institutional arrangements critically affect the feasibility, implementation, and sustainability of PHC reform.^107^^,^^109^ Legal frameworks, decentralization models, and governance structures shape not only how services are delivered but also how resilient systems are in the face of emergencies. While many countries in LAC enshrine health as a constitutional right, major implementation gaps persist. In Brazil and Chile, constitutional and legislative advances have not always translated into equitable access to services, while in Mexico, legal mandates have historically excluded key populations from social security schemes.^122^

These structural gaps are closely tied to the institutional architecture of health financing in the region, which presents significant barriers to sustained investment in PHC. Most countries operate within rigid fiscal constraints and fragmented financing arrangements that hinder their capacity to protect or expand PHC budgets.^25^^,^^28^ Public health spending remains chronically low, averaging just 3.6% of GDP compared to 6.6% in OECD countries, and is further undermined by persistent under-execution, limited absorptive capacity, and weak planning systems.^25^^,^^28^ Binding fiscal rules, earmarking practices, and short budget cycles exacerbate these constraints, preventing the adoption of long-term investment strategies needed to transform PHC.^25^ In addition, countries often lack institutional mechanisms to mobilize emergency funds or coordinate fiscal responses across sectors, while procurement, auditing, and accountability capacities remain weak. Even when spending temporarily increased during COVID-19, these flows were enabled through ad hoc emergency measures, without structural reforms to fiscal governance or intersectoral coordination. As these exceptional funds expire, the region’s shrinking fiscal space forces governments to make difficult trade-offs, frequently at the expense of PHC, which remains politically marginal and institutionally fragile.

These fiscal and administrative shortcomings are not isolated, but symptomatic of a deeper and persistent pattern of weak state capabilities and noncooperative policymaking equilibria across the region.^123^ Political systems in LAC are frequently marked by fragmented, low-trust relationships between stakeholders, where distributive conflicts are seldom resolved through stable, long-term agreements, but instead through discretionary, ad hoc, and inefficient responses. This broader institutional fragility is clearly visible in the health sector, where segmented systems, overlapping functions, and unequal financing structures continue to undermine both efficiency and equity.^124^ The lack of coordination, transparency, enforceable entitlements, and integrated planning across contributory and non-contributory schemes generates systemic inefficiencies and reproduces structural inequalities. As Guizzo-Altube et al. argue, these dynamics not only obstruct the design and implementation of coherent PHC reforms but also erode public trust and state legitimacy in the long run.^123^

Data from Transparency International indicate that most countries (18 of 30) in LAC scored below the regional average (40.3 points) in the 2024 Corruption Perceptions Index (CPI), pointing to persistent governance challenges.^125^ Corruption in the region is both a consequence and a driver of institutional weakness, influencing the allocation of resources, enabling rent-seeking practices, and reducing public confidence in government institutions, factors that can adversely affect health system performance.^43^ In contexts characterized by distributive tensions and political fragmentation, governments may increasingly rely on discretionary tools and non-transparent practices that undermine accountability and technical efficiency.^123^ These patterns were especially evident during the COVID-19 pandemic, where emergency procurement processes in several countries lacked sufficient oversight.^126^ In this context, corruption remains a significant structural challenge to improving the quality, efficiency and equity of health service delivery in LAC.^126^^,^^127^

**Fig. 11:**
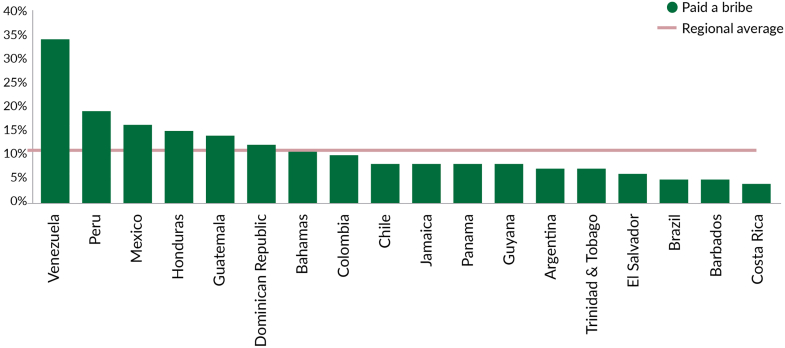
**Percentage of people who reported paying a bribe in public clinics or hospitals in the past 12 months across 18 LAC Countries (2019).** Note: Share of respondents who reported paying a bribe, giving a gift, or doing a favour to health workers in public clinics or hospitals to access care in the past 12 months (BRIBE2FIN). Source: Transparency International. (2019). Global Corruption Barometer: Latin America & the Caribbean 2019—Citizens’ Views and Experiences of Corruption. https://www.transparency.org/en/publications/global-corruption-barometer-latin-america-and-the-caribbean-2019.

Fig. 11 presents data from 18 countries in LAC on the proportion of individuals who reported making informal payments, such as bribes, gifts, or favours, to access care in public health facilities in 2019. Reported rates range from over 30% in countries such as Venezuela to below 5% in Costa Rica and Barbados. In contexts where informal transactions are common, vulnerable populations may face additional barriers to care, further entrenching health inequities and weakening institutional trust.

Similarly, the same report notes that 77% of respondents in the region express limited trust in government institutions, including politicians and public officials.^125^ These findings align with broader institutional dynamics in the region. Research suggests that when political and economic institutions are marked by high levels of inequality and centralized decision-making, the capacity of the state to deliver public goods may be reduced.^106^

Within this context of institutional fragility, entrenched structural and governance arrangements have constrained the scalability of intercultural and community-based PHC models in LAC. In countries such as Guatemala, El Salvador, and Paraguay, territorially based PHC reforms sought to expand access and promote community engagement, particularly among Indigenous and rural populations.^105^ However, these initiatives largely remained confined to the public sector, with limited response capacity, insufficient institutional integration, and high vulnerability to political and fiscal fluctuations. The broader configuration of health systems in LAC, characterized by segmented subsystems for formal sector workers, low-income populations, and high-income groups, has created institutional and financial silos that hinder the integration and expansion of inclusive models.^3^^,^^19^^,^^25^ In addition to undermining the reach and continuity of care, these systemic limitations have also impacted public trust.

As documented by Pinto et al., populations facing disadvantage, particularly Indigenous, Afro-descendant, and low-income groups, report experiences of discrimination, limited coordination across services, and insufficient cultural responsiveness, which may contribute to perceptions of exclusion and inefficiency.^98^ Even in settings where formal coverage has improved, perceptions of inaccessibility and low responsiveness have led to what some refer to as a “discouragement effect”, whereby individuals delay or forego care.^98^ The lack incorporation of patient perspectives into the design, implementation and evaluation of health services further compounds this dynamic.^98^ As a result, the persistence of fragmented, short-term, or pilot-based interventions, without sustained institutional commitment, undermines not only the reach and effectiveness of PHC models but also may diminish public trust in the health system itself, and may reinforce long-standing perceptions of exclusion, inequity, and unreliability.

These patterns may reflect broader institutional conditions. Evidence suggests that in contexts marked by limited State capacity and high levels of political polarization, health sector reforms are more likely to encounter implementation challenges, including fragmentation, limited coordination, and inconsistent follow-through.^123^ The quality of political and administrative institutions has also been closely associated with broader development trajectories. For instance, Vianna and Mollick find that even modest improvements in institutional quality, such as a 0.1-point increase, are linked to a 3.9% rise in per capita GDP in Latin America, compared to 2.6% globally, highlighting the region’s heightened sensitivity to institutional constraints.^128^ Complementing this, de Almeida et al. emphasize the central role of political institutions in shaping economic outcomes in LAC, and identify a feedback mechanism through which economic shocks may place additional strain on institutional capacity and performance.^129^

#### Ideas and ideology: competing models of PHC and their policy framing

The ideas regarding the conceptual foundation of PHC reform reflects competing policy paradigms. While the Alma-Ata Declaration (1978) emphasized comprehensive, community-based PHC, a more selective PHC, focused on narrow, cost-effective interventions, has gained traction among some groups of policymakers.^130^ Political ideologies have significantly shaped government priorities, reform agendas, and health system design in LAC. For instance, Brazil’s Unified Health System (SUS), which emerged from a broad-based social movement for health as a right, exemplifies how a coalition can institutionalize PHC-oriented reforms. Also, Colombia’s Law 100 of 1993 created the social insurance system with a relevant role for private actors but took until the Law 1438 of 2011 to formally recognize PHC as the backbone of the system. These historical trajectories highlight the role of political will and ideas in shaping both the design and sustainability of PHC system.

During COVID-19, Litewka and Heitman emphasized that ideological biases and political denial across the Latin American political spectrum critically undermined early responses to the pandemic, delaying containment measures and eroding public trust.^112^ Some leaders of different ideologies either dismissed scientific warnings or demoted the implications of the pandemic, fostering misinformation and public confusion.^113^^,^^117^ These ideological stances were not merely rhetorical but had tangible consequences on policy, governance, and public health outcomes, as they discouraged timely mitigation strategies and deepened societal divisions.

The limited progress in implementing PHC in LAC cannot be solely attributed to a lack of technical knowledge or consensus on its importance. Rather, it reflects enduring structural and institutional barriers, such as political and economic instability, fragmented governance, and persistent power asymmetries, which have constrained meaningful reform. As Acemoglu and Robinson argue, extractive institutions that concentrate authority and limit accountability undermine the state’s capacity to provide inclusive, equitable services.^106^ The COVID-19 pandemic further exposed the fragility of health systems and the risks of negatively politicizing public health.

## Part III: PHC resilience framework, and policy recommendations

### Conceptual framework elaborating on resilient PHC-based systems

**Fig. 12:**
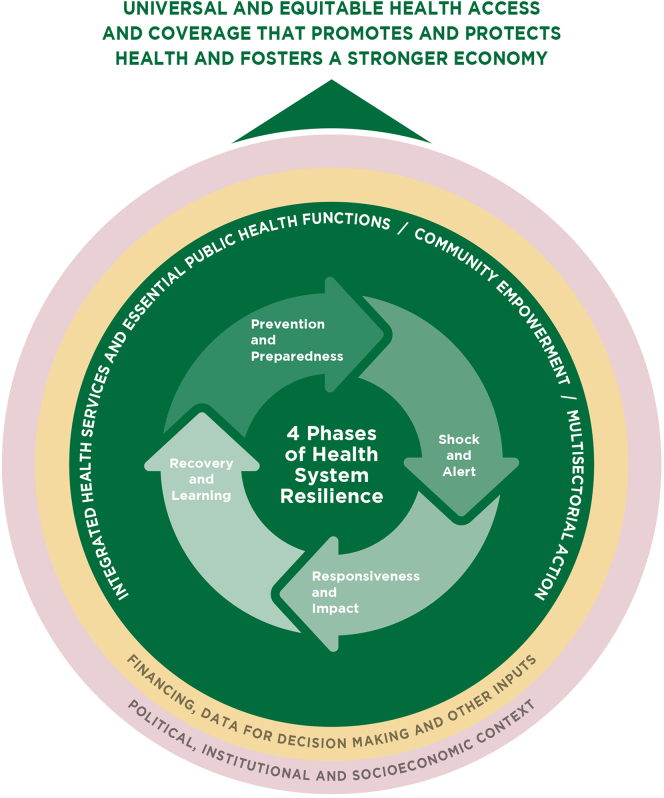
**Conceptual framework for resilient PHC-based systems**.

The Commission is guided by a conceptual framework that elaborates on the intersections and interlinkages that draw together PHC-based systems and resilience thinking. The policy framework (Fig. 12) offers theoretical considerations and a common understanding for our inquiry. Ultimately, it illustrates how ideas spanning primary care, resilience, health systems, and broader socio-economic and political factors work in synergy towards the goal of universal and equitable health access and coverage for all—before, during, and after shocks.

Foregrounded in the conceptual framework are the four phases elaborated by Paschoalotto et al. that define PHC-based systems resilient to shocks.^131^ This resilience cycle offers concrete and intuitive phases, including 1) Preparedness, which entails anticipating and making plans to address different types of shocks. This phase has sometimes been supplemented by an additional element—“prevention”—that involves seeking to avert public health shocks; 2) Shock and alert, that refers to the emergency itself, understanding the nature of the event, and alerting relevant stakeholders to the shock; 3) Responsiveness and impact, where the effect of the shock is addressed, plans are put into action, and necessary adaptations are made in response to the shock; and 4) Recovery and learning, which involves taking stock of lessons from the event and building back, if possible, better. The cyclical nature of these phases emphasizes that while resilience is often conceptualized as both a process and an outcome, it is perhaps best considered, as Thu et al. propose, a learning process in response to an evolution of shocks and stress.^132^

The framework is grounded in three core components of PHC-based systems– integrated health services, community participation and empowerment, and multisectoral action—that work together towards a goal of achieving health for all and fostering a stronger economy.^75^ The component on integrated health services encompasses both health care delivery (e.g., essential services) as well as EPHF (e.g., surveillance systems, health promotion), given the critical role PHC plays in generating intelligence, such as identifying emergent outbreaks or disease trends in communities, as well as conveying information about preventing health risks in communities and working with communities to take an active role in their health and well-being. Indeed, community empowerment and participation have been central to PHC since before the Alma-Ata declaration, with ample evidence underscoring the importance of meaningfully partnering with communities to develop, implement, and evaluate PHC to improve health outcomes and empower people.^133^^,^^134^ By focusing on the needs of communities, PHC is a locus for multi-sectoral action to address the wider determinants of health and intersectionality that shape people’s health and well-being. Through multi-sectoral action, PHC can enhance EPHF, contribute to comprehensive health and other-sector’s programs, and improve overall health system resilience by working across sectors before, during, and after shocks.^135^ These core components in turn are shaped by the broader health system in which primary care operates. Critical health system elements, including governance, financing, and the organization of services such as referral networks, can either enable or hinder health system resilience. The framework also acknowledges the importance of context, shaped by both the people who compose health systems and the socio-economic and political circumstances which systems operate in, when understanding and planning for resilience. Political economy plays a particular role here as discussed in Part II and later in Part IV of this report.^131^

From here, we highlight the four pillars that guided the work and policy recommendations of the Commission. Three of these pillars, 1) Integrated health services and EPHF; 2) Community empowerment and participation; and 3) Multi-sectoral action, responds to the core components of PHC-based health systems described above. The fourth, Financing, reflects the critical importance of PHC financing arrangements to all of the other components.

### Recommendations for resilient PHC

Given the diversity, certainty, and cost of shocks facing PHC-based systems across LAC, defining practical areas for action is needed so countries in the region can invest effectively to strengthen PHC resilience. The policy recommendations presented here establish a direct link between PHC strategies and resilience. To our knowledge, this has not previously been addressed in such a structured and in-depth manner, nor have these strategies been advocated together before in order to promote stronger PHC-based systems and resilience simultaneously.

Drawing on policy analysis and real-world examples from across the region, we compiled key recommendations for resilient PHC-based systems in LAC (Refer to Supplementary Material 2, Commission methodology). As a package, these present a vision of the ideal capacities, resources, and systems necessary for a holistic approach across the resilience cycle, while individually each is an actionable and synergistic step that countries can take to advance their resilience now with the resources and capacities at hand.

The analysis draws on four key areas of 1) integrated service delivery and EPHF, 2) community engagement and participation, 3) financing, and 4) multi-sectoral action. The recommendations for each key area follow. Within each area an ideal scenario was synthesized with complementary policy options identified to support countries in achieving resilient PHC (Tables below, for each key area). A full description of these is presented in Supplementary Material 1: Domains, ideal scenarios, objectives, and policy options.

#### Integrated service delivery and EPHF

Effectively integrated with specialized care networks and team-based primary care service delivery enhances capacity to address a broader range of holistic health needs across the life course, while deepening relationships, linking families and community organizations.^136^

Integration of EPHF can ensure greater coherence between primary care and public health to advance population health programs as part of routine care and crisis response.^137^ Integrated service delivery and ensuring delivery of EPHF require a commitment to models of primary care that provide services for all, including during crisis, integrating EPHF into operations, systems, and processes, while maintaining essential health care services in primary care and ensuring robust and appropriate engagement with different levels of care in the specialized health care ecosystem (Table 2).

**Table 2:** Ideal scenario and summary of policy options for integrated health services and EPHF for resilient PHC.

Domain	Ideal scenario	High-level summary of policy options^a^
• Models of primary care that provide services for all	A clearly established comprehensive, equitable and evidence-informed model of primary care in which the population living in each primary care facility’s catchment area is registered, and the service provision can be universal, comprehensive and oriented to meet people’s needs in a culturally competent way addressing inequalities.	1. Mechanisms to identify and screen specific populations (e.g., vulnerable or priority groups).^138^^,^^139^ 2. Interprofessional teams for collaborative care.^140^ 3. Community outreach for preparedness and response.^138^^,^^141^^,^^142^ 4. Quality management and improvement systems for primary care services. 5. User feedback mechanisms.^143^
• Integrating EPHF into primary care	Agencies responsible for providing EPHF such as surveillance, disease prevention and health promotion work seamlessly with the PHC-based system, and many of these EPHF (including effective surveillance and health promotion) are embedded within primary care to effectively address the social determinants of health.	1. Training programs for primary care workers in data analysis and information system adaptation. 2. Specialist epidemiological teams to support primary care facilities in health surveillance.^137^ 3. Regular health situation assessments and collaboration with communities to address health priorities.^139^^,^^144^ 4. Health education programs to address social determinants of health 5. Primary care units as sentinel sites for early outbreak and risk detection.^144^^,^^145^ 6. Diverse communication strategies for primary care staff to inform communities and combat misinformation.
• Maintaining essential health care services in primary care	Primary care facilities are seen as an integral part of crisis response and are equipped with necessary resources and skills. PHC-based systems prioritize the provision of equitable care and the protection of vulnerable populations, ensuring the continuity of essential health services, as well as an effective response to the crisis.	1. Emergency response plans to ensure primary care access during crises.^146^ 2. Equitable primary care access through digital services, adapted schedules, and community outreach.^137^^,^^147^, ^148^, ^149^ 3. Primary care workforce training and modified patient flows for effective crisis response.^150^, ^151^, ^152^, ^153^, ^154^ 4. Necessary infrastructure and equipment for essential primary care services.^155^^,^^156^ 5. Plan and support adequate health care workforce for crisis situations.^154^^,^^157^, ^158^, ^159^, ^160^ 6. Effective distribution and supply chain for medicines and vaccines.^157^, ^161^
• Ensuring that the specialized health care network engages appropriately with primary care	Primary care services are the cornerstone of the health system and are effectively integrated with specialized care networks. Referral networks, strong information systems and communication channels exist between primary care facilities and specialized health services, in order to ensure continuity of care and the most effective and efficient use of resources.	1. Systems for post-discharge support with communication channels and dedicated staff.^162^, ^163^, ^164^, ^165^, ^166^ 2. Robust referral systems to higher-level facilities, including patient transport and digital technology.^149^^,^^167^ 3. Primary care facilities supplied with necessary inputs, equipment and medicines to avoid unnecessary referrals. 4. Health professionals trained to provide effective and quality clinical services in primary care.

a Full policy options can be found in Supplementary Material 1.

##### Models of primary care that provide services for all

Resilient PHC relies on strengthening accessible, integrated, comprehensive, evidence-based, and equitable models of care, delivered by interprofessional and culturally sensitive primary care teams.^140^ Given the diverse regions, cultures, languages and communities in LAC, composition of care teams should reflect local needs and conditions and include diverse skillsets across medical doctors, nurses, community health workers (CHWs), social workers and others, all working with a commitment to collaborative care. Models of care delivered by these teams must be configured to ensure primary care service provision across the resilience cycle so that people can access the care they need, when they need it, in their communities, even during crisis. This requires ways to identify and support vulnerable populations, interprofessional teams and community outreach, with mechanisms for quality assurance and user feedback, and training for empathic and culturally sensitive delivery.

One essential strategy to ensure continuous access to care is geographic empanelment, which assigns people to a particular healthcare team or facility.^141^^,^^142^ Empanelment requires mechanisms by which to map or otherwise identify people who may be vulnerable to shocks and emergencies, in particular vulnerable or priority populations, including, but not limited to, older adults, migrants or displaced people, people living in long-term care or people with disabilities.^168^ For example, in Brazil, Chile and Costa Rica, empanelment is a foundational part of the health system, where primary care teams are assigned to specific geographic areas and are responsible for the health needs of their communities.^169^, ^170^, ^171^ Ideally, these mechanisms would include ways to rapidly screen populations for potential and existing vulnerabilities that shape their access to and need for primary care.

Another strategy for integrated service delivery is community outreach. Building trusted relationships between communities and primary care is key to bridging gaps in care, expanding access to services, and creating important opportunities to enhance preparedness. Partnering with organizations for home-based care, schools, and other community associations creates opportunity to develop the necessary trusted relationships and adaptive capacities for maintaining accessible and person-centred care during crisis.^138^^,^^141^^,^^142^

Resilient care is responsive to user’s needs, and ensuring these strategies are effective requires robust quality management systems that incorporate monitoring tools and improvement mechanisms to understand processes, outcomes, and user experiences as a way to improve primary care delivery.^172^ In Belize, for example, the quality improvement systems and tools established before the pandemic allowed improvement efforts to continue throughout the crisis.^173^

##### Integrating EPHF into primary care

Realising fully integrated EPHF means equipping primary care workers with the training, skills, and systems necessary to detect and respond to vulnerabilities within their catchment communities. Integrating EPHF into primary care has shown potential benefits, including improved service delivery, increased access to care in remote and rural communities, and reduced costs through shared resources.^77^ This requires training and support, ensuring that primary care workers can conduct regular health situation assessments and support early threat detection, while addressing misinformation and contributing to broader population health and health promotion activities.

Providing training, tools and technical support to primary care workers in data analysis and adapting information systems is essential for enhancing their capabilities. Specialist epidemiological teams from Health Departments or National Public Health Institutes can support primary care facilities in health surveillance, ensuring that they are well-equipped to monitor and respond to health threats with adequate surveillance tools.^137^^,^^174^ Well-integrated EPHF requires seamless coordination between primary care and responsible agencies, from local to national, with information flows for regional and global pathogen, vector, or other risk monitoring. More broadly, primary care teams must also be a part of collaborative mechanisms or governance structures that ensure effective local-level multi-sectoral collaboration for preparedness and response.

Regular health situation assessments conducted by primary care facilities help detect risk factors and vulnerabilities. These assessments enable primary care workers to collaborate with communities to address priority health problems and strengthen participation, such as through community resource maps.^139^^,^^144^ Teams can then draw on this information to co-develop, test, and evaluate preparedness and response plans tailored to local conditions, that effectively leverage PHC capacities for risk management and crisis response. PHC can also be leveraged for sentinel surveillance, providing essential information on usual disease or deaths in communities across LAC.^144^^,^^145^ Across LAC, examples are emerging of the benefits of integrating EPHF and primary care–for example, the Practical Approach to Care Kit (PACK) program in Florianopolis, Brazil equipped primary care teams with contextualized, comprehensive training and up-to-date resources (clinical guidelines, protocols, and decision support tools) on how to manage emerging infectious disease outbreaks.^175^ From 2015 to 2023, primary care teams drew on their PACK training to respond to five infectious disease outbreaks—leishmaniasis, Zika, COVID-19, mpox, and dengue. Through enhanced data collection and management practices, primary care teams were instrumental in tracking disease outbreaks and health outcomes, strengthening overall surveillance in their communities and region.

As recent pandemics such as COVID-19 and mpox have underscored, shocks of the 21st century are accompanied by an equally dangerous information crisis—misinformation, rumours, and fake news can surge at an unprecedented scale, complicating response efforts and damaging trust.^143^^,^^176^ Given that primary care should be a trusted and familiar information source in communities across LAC, primary care workers must be able to provide effective and clear communication about risks, prevention measures, and responses. Primary care workers should be familiar with, and able to quickly and consistently access multiple means of disseminating information in physical and online spaces. The primary care workers responsible for these activities must be equipped with consistent messaging, ideally co-developed with community leaders or groups, to mitigate disinformation and deliver accurate information in ways that reflect local cultures, values, and beliefs (further explored in later sections).^177^ Even within populations with low levels of trust, most people tend to trust the information provided by health workers.^178^

While shocks and crises may bring to mind recent pandemic responses that bridged primary care and public health activities, efforts to fully embed EPHF into primary care must take a broader view of shared goals to improve population health and well-being. Programs and interventions for health education, health promotion and disease prevention can be embedded in primary care, along with activities that address the social (and other) determinants of health. In this way, integrating EPHF into primary care also reinforces PHC-based systems, while building resilience through healthier societies. See Panel 7 for the evolution of PHC in the city of Rio de Janeiro, Brazil integrating EPHF to respond to several shocks in recent years.

Panel 7 **Family health teams and integrating PHC and emergency preparedness and response: the case of Rio de Janeiro, Brazil.**

In recent years, the city of Rio de Janeiro, Brazil has faced extreme events that challenged its health system: arbovirus epidemics, armed violence, heatwaves, and, most critically, COVID-19, which exposed overlapping vulnerabilities and demanded articulated responses. Until 2008, the municipal health system was hospital-centred, with a weak and fragmented PHC component. The 2008 dengue epidemic, with over 235,000 cases, revealed systemic fragilities and led to federal intervention in local health management.

In response, starting in 2009, PHC was restructured as the backbone of the system through territorial expansion, team training, and integration with health surveillance. In less than a decade, coverage by Family Health Teams (FHTs) went from 3.5% in 2008 to surpass 70% in 2016 and reaching over 80% in 2024, with improvements in infant mortality, avoidable hospitalizations, and chronic disease control.^179^

The city’s COVID-19 response unfolded in two phases. At the beginning of the pandemic, PHC faced the effects of disinvestment, staff shortages, supply deficits, and weak emergency preparedness since the late 2010s. From 2022, PHC enhanced its role in testing, contact tracing, protecting vulnerable groups, maintaining routine care, and supporting vaccination. This experience consolidated real-time data use, readiness routines, and integrated surveillance and care.^180^

In 2024, a new dengue epidemic hit with over 100,000 cases. PHC functioned as gatekeeper, managing demand, identifying severe cases, and maintaining essential care—such as antenatal visits and chronic disease follow-up—reducing pressure on emergency services.^180^ In addition, heatwaves have become another growing challenge for health systems. The city developed contingency plan, including hydration stations in health facilities and public areas, vulnerability mapping, and intersectoral coordination with civil defence, education, and social services—enhancing anticipatory and mitigative capacities.

The post-crisis period became an opportunity for organizational transformation. Institutionalizing lessons learned and embedding resilience practices, PHC in Rio de Janeiro evolved from a point of entry to a centre of response, continuity, and innovation.

##### Maintaining essential health care services in primary care

Across the resilience cycle, primary care must be able to maintain essential health care services in communities. While being a core partner for an effective multi-sectoral crisis response, primary care must be reinforced to ensure continuous provision of equitable and high-quality essential care, particularly for priority or vulnerable populations. Maintaining essential primary care services requires emergency response plans to assure access to services, crisis response and infrastructure, as well as workforce and supply chain management.

Well-established and tested emergency response plans are needed to keep primary care facilities open and maintaining access to essential services during emergencies and ensuring that decisions to close or curtail primary care are purposeful and prioritize equity, clarity, and sustainability. Effective, safe, and equitable access to PHC services can be achieved through digital and remote services (including remote monitoring), telemedicine, teleassistance, and telehealth. COVID-19 highlighted the many ways that adapting primary care service provision can build resilience to shocks and maintain essential health care services, namely through remote and digital means.^181^ For instance, access to telemedicine partially offset the reduction of health services for patient with chronic conditions in Colombia^182^ (more on Panel 8: Telemedicine for continuous access to care in communities in Panama).

Panel 8 **Telemedicine for continuous access to care in communities in Panama.**

Panama’s TeleSalud initiative highlights a journey of resilience and innovation during the COVID-19 pandemic.^183^ Telehealth became an important tool, ensuring uninterrupted primary care services, especially for vulnerable populations in remote areas. Despite Panama’s high-income status, significant socioeconomic inequalities persist, particularly affecting Indigenous communities. The Ministry of Health (MINSA) led the telehealth initiative with a comprehensive Telemedicine Master Plan and regulatory framework. Initially, services were centralized through a Contact Centre, training health professionals for teleconsultations. The program later expanded to regional areas, addressing barriers like limited digital literacy and access to technology through continuous evaluation and international collaborations. The results were impressive: over 137,000 teleconsultations and 387,000 prescriptions were issued between 2021 and 2024. Telehealth enhanced resilience by improving preparedness, prevention, response, and recovery during health emergencies, integrating primary care with specialized care, and highlighting the importance of hybrid care models. Moving forward, sustaining these gains requires robust governance, financing, and a people-centred approach.

While digital technology is increasingly relied on for care provision, its use must consider the vast digital inequities and service fragmentation across LAC.^184^ For example, in 2022, 67.3% of LAC households had internet access, compared to 91.1% in OECD countries, and rural women were found to be 37% less likely than men to have internet access, underscoring the need for inclusive and contextualized approaches and a commitment to comprehensive digital transformation in primary care (more on Panel 9: Digital Transformation of PHC in São Paulo, Brazil).^184^, ^185^, ^187^, ^188^ The artificial intelligence (AI) revolution is also important to consider–for example, a study in Costa Rica found that integrating AI into PHC-based systems can considerably improve NCD care prediction, and when complemented with modelling and digital monitoring systems, can facilitate early risk detection and personalized care.^186^ Adapted schedules and strategies for community communication and participation, out-of-hours services, and providing services in open air or other solutions to prevent infections are also important. Increased options for providing care at home and in the community, including CHWs, mobile clinics, and outreach, as well as ensuring that primary care facilities have scheduling systems to reduce waiting times and prevent inequalities, are essential.^137^^,^^147^, ^148^, ^149^

Panel 9 **Digital transformation of primary care in São Paulo, Brazil.**

The Avança Saúde SP program (2019–2024), supported by a $200 million investment, showcases São Paulo’s innovative strategy to enhance the resilience of its PHC-based system in 53 health units.^185^^,^^186^ By tackling systemic vulnerabilities and harnessing digital transformation, the initiative has developed a more adaptive, integrated, and data-driven health network, capable of addressing various public health challenges. The E-Saúde SP platform unified clinical and administrative data for over 26 million individuals by September 2023, enhancing patient engagement, care coordination, real-time decision-making, and public health management. This centralized approach improved the health system’s capacity for surveillance and long-term planning. A standardized risk classification system and expanded telecare services, including hybrid models and home visits, improved patient flow and access to care, with 1.7 million consultations recorded. Advanced administrative systems enhanced operational efficiency, transparency, and accountability. The E-Saúde SP app empowered individuals with tools for chronic disease monitoring, vaccination tracking, and self-care, fostering community resilience. By connecting health services with broader socio-economic structures, the program illustrates a scalable approach to fostering resilience, equity, and sustainability in health care delivery.

When responding to a crisis caused by a pathogen, it is essential to ensure that PHC-based systems are capable of responding to mild and moderate cases. This includes training programs for the health workforce, modifying patient flows through facilities to separate infectious and non-infectious populations, and communicating to the population when they should seek primary care and when it is appropriate to go to higher-level facilities. Referral policies to other levels of care for high-priority cases should also be in place. Having the right infrastructure and equipment available at primary care facilities will better maintain essential health services during a shock. This includes internet connections at all primary care facilities, safety materials for health professionals during a crisis (e.g., personal protective equipment), vaccines and medicines (ensuring an effective cold chain), information backup services, and guidelines and protocols to efficiently provide health care services. For instance, Costa Rica has significantly improved its primary care infrastructure by increasing the coverage of its single digital health record system (EDUS) from 50% to 90% of the country’s primary care facilities between 2016 and 2023.^189^

To ensure resilience, it is essential that primary care facilities have an adequately staffed and interprofessional health workforce to deliver team-based essential health services before, during, and after shocks. This includes adequate health professional planning, with plans in place in advance for hiring to ensure proper staffing during crises and having established systems for all primary care workers to access up-to-date treatment protocols and necessary training. Training health workers to respond to the consequences of crises, such as mental health and gender-based violence, providing extra allowances or incentives (e.g., compensatory time off), and offering mental health care services for health professionals to cope with occupational stress and burnout are also important. This was underscored across LAC during COVID-19, as countries introduced hazard pay, compensated time off, and additional allowances for primary care workers.^40^

A well-equipped primary care workforce requires well-equipped facilities to safely and reliably provide care. For example, providing essential health services relies on readily available medicine and vaccines.^157^, ^161^, ^190^ This includes effective distribution of medicines to patients, especially those with chronic conditions, and maintaining an effective supply chain and logistics for medicines and vaccines. These same systems may also be called upon to support crises response, so they must be flexible enough to accommodate public health and social measures that may limit access to care during crisis. For example, during COVID-19 home delivery services or more convenient community pick-up sites were used to ensure continuous access to medications.^191^ This requires policy and regulatory flexibility to allow for responsive and innovative solutions during crises. For example, in Mexico, prescribing restrictions were eased during the pandemic and patients were given refillable electronic prescriptions for their chronic disease medication.^192^ In addition, primary care procurement, storage, and dispensing systems must be flexible to provide both routine medicines, as well as support the distribution of medical countermeasures that may be used to respond to a pandemic or outbreak.^189^

##### Ensuring that the specialized health care network engages appropriately with primary care

Primary care should be the cornerstone of the health system, effectively integrated with, and supported by, specialized care networks to ensure that those seeking care at PHC can be referred to other levels of care as needed, while also returning from other levels of care to primary care. Yet, a recent study of eight LAC countries revealed that 17.4% of public hospital discharges are for conditions that could be managed in primary care—also known as ambulatory care sensitive conditions (ACSC).^74^ Improving ACSC management in primary care could reduce ACSC hospital discharges by 1–2% on average. To realize these gains, networks, strong information systems and communication channels must exist between primary care facilities and specialized health services, in order to ensure continuity of care and the most effective and efficient use of resources. This requires robust regulation tools and logistics, post-discharge support systems, referral systems with support from higher-level facilities, and ensuring primary care workers are well-equipped and trained.

Establishing systems to provide appropriate support after hospital discharge is essential for ensuring continuity of care.^162^, ^163^, ^164^, ^165^, ^166^ This includes creating multiple communication channels (e.g., reference and counter-reference) between different-level facilities, such as hospitals and specialty services. Dedicated staff can act as case managers, for instance, under navigation programs that accompany patients across the care continuum from primary care to hospital care. Case managers in primary care can support in managing referrals and counter referrals, discharge planning, and clinical protocols for post-discharge care.^193^^,^^194^

Developing robust referral systems ensures coordinated movement between levels of care—from primary to specialized care and back again. These systems should include patient transport between primary care and higher-level facilities, information flows, and direct communication between levels (counter-referral). Digital technology can support referral systems, and effective waiting-list management is necessary. It is important to align medicine supplies and diagnostic capacities with referral rules and conditions to be treated at the primary care level. Additionally, effective communication channels between higher-level facilities and primary care should promote the dissemination of up-to-date treatment guidelines.^162^, ^163^, ^164^, ^166^

Supplying primary care facilities with the necessary inputs, equipment, technologies, and medicines is essential for providing effective and quality clinical services, thereby avoiding unnecessary referrals. Ensuring that health professionals are well-trained to deliver these services is equally important. This combination of well-equipped facilities and skilled professionals helps maintain high standards of care at the primary care level.

#### Community participation and empowerment

Given the close ties between primary care and the communities it serves, fostering PHC resilience demands that trust and community empowerment are amplified both during the day-to-day provision of services, as well as during emergencies.^195^^,^^196^ A recent expert consultation and scoping review of 70 studies on community-driven interventions during public health emergencies in LAC found that factors associated with successful community-driven interventions included strong community ties, local knowledge, established networks, and trust.^197^ The same review found pressing challenges to be removed through policy action, including technological barriers, digital literacy issues, limited cultural adaptations, gaps in intersectoral collaboration, funding sustainability, restricted participation spaces, and safety concerns.

Institutionalizing opportunities for meaningful engagement is essential, as is respecting cultural diversity, and ensuring transparent communication and accountability. Efforts are needed to ensure that PHC-based systems, communities, and governments are working together for interlinked and mutually reinforcing preparedness and response. In this way, community-driven responses and assets can be well-integrated into decision-making to strengthen primary care capacity in responding to external shocks. Table 3 offers a summary of ideal scenarios for community engagement in resilient PHC, with complementary high-level policy actions.

**Table 3:** Ideal scenario and summary of policy options for community participation/empowerment for resilient PHC.

Domain	Ideal scenario	High-level summary of policy options^a^
1. Community engagement	Institutionalized opportunities for meaningful community engagement in PHC-based systems that include dedicated spaces for decision making, knowledge exchange and innovation between communities and health care providers, including community health workers, which facilitates the co-ownership and co-responsibility in facing external shocks, as well as transparency and accountability in decision-making processes.	1. Institutionalize community participation in public health planning. 2. Create regular dialogue, knowledge exchange, (Diálogo de Saberes) and decision-making spaces between communities and PHC-based systems.^188^^,^^198^^,^^199^ 3. Ensure legal frameworks through participatory oversight mechanisms. 4. Hold quarterly meetings for community and PHC-based system planning. 5. Maintain continuous emergency preparedness and actor mapping.^139^ 6. Develop community-based innovations for resilience building.^200^, ^201^, ^202^ 7. Disseminate engagement resources for community resilience. 8. Train primary care managers in community engagement priorities.^203^ 9. Involve CHWs in transparent, participatory decision-making.^142^^,^^204^ 10. Support CHWs in primary care delivery and emergencies.^60^^,^^137^^,^^158^ 11. Empower communities for health promotion during emergencies.^205^ 12. Leverage community networks for broader intervention reach.^60^^,^^137^^,^^158^ 13. Partner with CSOs for public health emergency response.^158^^,^^205^
2. Interculturality	Culturally representative primary care services, integrating diverse cultural and ancestral health practices in building resilience.	1. Develop intercultural health policies promoting traditional medicine. 2. Finance intercultural primary care services with community participation. 3. Integrate interculturality in continuing education and training. 4. Recruit diverse health personnel reflecting local communities.^188^ 5. Create intercultural spaces integrated with health centres.
3. Communication and trust	Consistent, transparent and respectful communication between primary care providers and community members, fostering deep trust and engagement, in order to engage community assets and capacities to respond to external shocks and tackling miscommunication and fake news.	1. Co-create health messages with trusted community representatives. 2. Develop culturally appropriate, multilingual health communication materials.^198^ 3. Use multiple channels to reach diverse populations.^206^

a Full policy options can be found in Supplementary Material 1.

##### Community engagement

Community participation and mobilization are needed for resilient PHC-based systems.^207^ Institutionalized opportunities for meaningful community engagement in PHC are important. The spirit of partnership with the community for resilient PHC must be predicated on co-ownership and co-responsibility in facing external shocks, as well as transparency and accountability in decision-making processes. This requires legal and regulatory frameworks for community participation, forums for engagement and capacity building, and a commitment to training and support.

Establishing legal and regulatory frameworks that institutionalize opportunities for community participation is essential. Across LAC, community participation in national deliberative spaces on health is becoming increasingly common. For example, in Chile, citizens and CSOs actively participated in the constitutional reform process by submitting health-related proposals for constitutional norms through the Popular Initiative for Norms platform. However, it was not mandatory for the Constitutional Convention to consider them, leaving the final call up to the political conformation of the Convention.^208^ During COVID-19, mobilization deepened with CSOs and scientific entities created collective movements advocating for government accountability during the pandemic in Brazil.^209^ More can be done in the region to guarantee social participation through legal frameworks. Consideration must be made to ensure sustainable funding, transparency and accountability measures so that community engagement is not an ad hoc or token event, but rather an institutionalized process that informs service design, delivery, and evaluation, as well as preparedness and response planning. Part of these efforts should include inclusive and participatory oversight so that communities are active partners in accountability mechanisms that ensure legal and regulatory frameworks are applied at all levels of decision-making.

Forums for regular engagement between communities and primary care providers and other partners are necessary to plan, implement, and evaluate programs. Institutionalized opportunities for community participation should establish permanent spaces for regular dialogue and knowledge exchange between communities, primary care teams, and government stakeholders including decision-making. Panel 10 explores patient organizations as one example of community participation. Partnerships must include a breadth of local voices and community groups, while also ensuring open lines of communication to decision-makers at sub-national and national levels so that planning at the local level links to and can be supported by broader planning efforts. For example, primary care preparedness can include recurring meetings for community input into local plans with primary care (or otherwise) to prepare and respond to public health emergencies. For example, during the COVID-19 pandemic in Argentina, neighbourhood emergency committees were established as solidarity networks that developed plans, coordinated with the health sector and municipal organizations, and provided social support.^215^ These committees played a major role in distributing medicines and offering assistance during job losses, food crises, and the suspension of public health services.^215^

Panel 10 **Patient and user organizations in LAC for resilient PHC-based systems.**

One of many ways to involve communities is through patient and user organizations. These emerged in the 1950s in response to chronic diseases and lack of support.^210^^,^^211^ Across LAC, these organizations have since evolved to play an increasing role in patient empowerment, health technology assessment, and health decision-making.^212^^,^^213^

Bolivar et al. analyses the role of patient and user organizations in building resilient health systems in LAC.^214^ A mixed-methods approach is used in a sequential explanatory design that was developed through a survey and focus groups. The findings indicate that while many organizations do not explicitly identify the concept of resilience in health, in practice they already develop key actions. The words most associated with the concept of resilience include capacity, access, equity, support and empathy, although there are regional differences in their perception.

Three dimensions of PHC in which patient and user organizations contribute to resilience were identified:

1. Social Determinants of Health: organizations make inequities visible and promote policies to guarantee the right and equitable access to essential services, through intersectoral articulation and collaboration and strengthening of community capacities to demand integrated responses.
2. Empowerment and Education: organizations facilitate support networks, train patients and caregivers, and promote self-care and community participation, strengthening social resilience.
3. Health Systems: organizations act as mediators between patients and the health system, assisting in the management of appointments, complaints and evaluation and monitoring of the quality of care, contributing to improve care models.

The study concluded that to consolidate the role of patient and users organizations in health system resilience the following can be pursued: strengthen their recognition in the health structure, ensuring their participation in decision-making; provide funding and training to expand their impact and improve their capacity to respond to crises; integrate them in PHC strategies, recognizing their role in social determinants, empowerment and access to health; and establish monitoring and evaluation mechanisms to measure their impact and make their contribution visible.

Community participation also includes ongoing efforts to identify and include all groups. Continuous mapping of community actors, knowledge systems, and of capacities can ensure that no one is left behind when preparing for or responding to crisis, as well as in planning routine services. Partnerships remain dynamic and reflective of the communities they represent and serve, and they leverage collective capacities.^139^^,^^216^ This approach also supports the co-design, co-development, and effective dissemination of community-based innovations that link and reinforce community resilience with PHC resilience, and broader health system resilience.^200^, ^201^, ^202^

Meaningful community engagement is a process that requires investment in, and ongoing commitment to, training and supporting the diverse teams undertaking this important work.^203^ This includes drawing a wider circle around where primary care is delivered and by whom. For example, institutionalizing fair and just partnerships with CHWs and ancestral providers can engage them in decision-making and establish transparent and participatory ways of working with these providers under both routine and emergency conditions. Numerous examples across LAC and globally demonstrate how CHWs link communities to primary care.^217^, ^218^, ^219^ These highlight how trained CHWs have been leveraged to support diverse primary care programs, while also supporting EPHF, and reaching even the most vulnerable communities.^220^^,^^221^ In Guerrero, Mexico, and Managua, Nicaragua, Camino Verde, a pragmatic parallel group trial of pesticide-free dengue vector control, collaborated with local community members known as *brigadistas*.^222^^,^^223^ The *brigadistas* visited homes and schools, organized educational activities, and raised awareness about dengue transmission to help mobilize the community against the disease, and the program led to a significant reduction in dengue infections (29.5% in Guerrero, and 24.7% in Managua).^222^

By equal measure, community members, leaders, and CSOs must be supported for meaningful and mutually reinforcing participation. Through shared decision-making they enhance PHC-based systems and their efforts to improve services and influence decisions are enhanced in return. This is important during routine conditions and particularly important during emergency responses where these groups are often first responders, demonstrating resilience by extending their capacities and resources to strengthen a circle of care around their communities. Through community engagement, such as that built by fostering resilient PHC, these community-led efforts can be met with comprehensive government support that amplifies resilience.

##### Interculturality

Latin America and the Caribbean are home to many diverse peoples, each with unique languages, cultures, and histories that shape their health and well-being, as well as their care needs. Culturally representative primary health services integrating diverse cultural and ancestral health practices are fundamental towards building PHC resilience. These must promote an intercultural approach in the organization of PHC-based systems recognizing the knowledge, practices and providers of diverse peoples and communities. It is critical to establish intercultural health policies and programs, ensure intercultural delivery of primary care services, while requiring education and training in interculturality.

Developing health policies and programs with an intercultural approach is essential for promoting traditional and complementary medicine and educating the public on intercultural health issues. These policies should aim to integrate diverse cultural practices into the health care system, ensuring that all community members feel respected and understood. Panel 11 describes the case of Guatemala’s Maya Q’eqchi’ people and their relationship with state-led disaster response. Often, CHWs are the cornerstone of intercultural links between PHC-based systems and communities. For example, in São Paulo, community health agents (ACS) in the Bom Retiro Basic Health Unit are essential to ensuring that immigrant communities can access care by bridging cultural and linguistic gaps.^224^

Panel 11 **Indigenous-led health justice and climate resilience in Guatemala.**

In Alta Verapaz, the Maya Q’eqchi’ people face a unique confluence of climate vulnerability, historical marginalization, and weak state response.^84^ Despite repeated weather crises, indigenous communities have been abandoned, and have not received minimal emergency assistance, forcing them to rely on local solidarity and self-organized response strategies. This case underscores the broader need for systemic change in state-led disaster response frameworks to ensure equitable access to health and emergency services.

PHC-based systems can be instrumental in strengthening climate resilience, yet the experience of Indigenous communities in Guatemala highlights significant failures. While government policies exist on paper, their implementation remains inadequate, particularly for rural Indigenous populations. The absence of timely and effective emergency response further exacerbates historical injustices, leaving these communities to bear the brunt of recurring climate disasters with limited institutional support. This pattern of state relinquishment not only threatens public health but also affects human rights, reinforcing systemic inequalities that have long marginalized Indigenous populations.

This case provides powerful lessons learned. First, recognizing traditional health practices, ensuring culturally competent care, and increasing investments in community-driven health initiatives are critical steps to strengthen PHC resilience across LAC countries. Secondly, national emergency response strategies should have an equity lens that could guarantee universal access to services, particularly for marginalized populations facing recurring climate shocks. Finally, an approach to health and emergency response that addresses the historical and structural barriers which continue to prevent Indigenous communities from receiving adequate state support is needed.

Establishing and financing are needed to ensure intercultural delivery of primary care services with full and effective participation of communities. This approach ensures that health care services are tailored to the cultural needs of the population, fostering greater acceptance and utilization of health services. Integrating intercultural approaches with health centre activities allow for the incorporation of traditional practices and community involvement, ensuring that health care delivery is holistic and culturally appropriate. Panel 12 provides examples from Bolivia of creating intercultural spaces for primary care.

Panel 12 **Interculturality in action in Bolivia.**

Bolivia’s traditional medicine, particularly the practices of traditional birth attendants (TBAs), is deeply intertwined with the cultural and spiritual identity of its communities.^225^ These ancestral practices, formally recognized by the 2013 Law on Traditional Ancestral Medicine (Law 459), are not merely health care methods but vital expressions of cultural heritage. During the COVID-19 pandemic, the community’s reliance on TBAs for home deliveries highlighted the enduring trust and respect for these cultural traditions. Post-pandemic policies now support collaboration and cross-referral between TBAs and doctors, although challenges remain in institutionalizing these practices across diverse Indigenous traditions and in mixed-ethnicity urban areas. This cultural integration not only provides essential health care but also reinforces community resilience and creates trust in the health system during crises.

Continuous education and training programs for health care workers are essential to formally integrate interculturality in primary care service delivery. These programs help health care professionals understand and respect cultural differences, improving their ability to provide culturally sensitive care. Training should ideally be led by community groups, should formally integrate interculturality to equip all primary care workers with the skills, knowledge, and practices to safely provide care.^226^ Importantly, primary care teams should strive to more closely reflect those they serve by recruiting and retaining health personnel who represent the ethnic and cultural diversity in their communities.

##### Communication and trust

Institutionalized opportunities for effective communication and trust-building in PHC-based systems ensure consistent, transparent, and respectful interactions between providers and community members. Fostering deep trust and engagement is necessary to engage community assets and capacities to respond to external shocks and tackling miscommunication and fake news. This requires a commitment to using respectful and dignified interactions and communications between primary care providers and communities, ensuring that health messages (including health risks and crisis communications) are culturally relevant and linguistically accessible. As well as facilitating communications from health authorities to the community by empowering communities to improve their health literacy and using trusted community leaders to sensitize populations to a variety of health risks. Institutionalized opportunities require co-creation and community engagement, culturally appropriate communication materials, and multi-channel communication with opportunity for feedback.

Creating communication materials that are culturally appropriate and available in local languages is key to delivering health and risk messages effectively. Co-creating the design and delivery of health and risk messages with trusted community representatives is essential for ensuring that these messages effectively reach diverse populations. By involving community representatives who are trusted to deliver health messages, the communication process becomes more credible and relatable, fostering better engagement and understanding. These materials should be tailored to the cultural context of the target audience, ensuring that the information is accessible and resonates with the community. Given the dynamic nature of information flows, particularly during emergencies, feedback channels should be established so that messages and dissemination plans can be tailored in real time to address rumours, misinformation, or to leverage emerging capacities.

Using multiple channels to communicate health and risk messages is important for reaching diverse populations. By leveraging various platforms, such as social media, radio, television, and community events, the reach of these messages can be maximized, ensuring that different segments of the population receive the necessary information. For example, during the COVID-19 pandemic in Guatemala, a social media-based mental health campaign was created with underserved communities through a participatory approach involving researchers, community-based mental health providers, and community members.^227^ The campaign had considerable reach (84,000 individuals), and the participatory approach ensured focused messaging relevant to the current experiences of community members.

#### Multi-sectoral action

Multi-sectoral policy and action involves a strategic and coordinated vision to address health determinants and threats through evidence-informed actions by multiple sectors to achieve optimal population health.^228^ Primary care is typically where multisectoral action gets put into practice. Indeed, primary care by definition is delivered close to the community, and it is at this point, that health will engage with education, agriculture, trade amongst other sectors to promote health. For example, in Costa Rica, a multisectoral program involving the Ministry of Health, the Costa Rican Social Security Fund, the Ministry of Education, and the National Children’s Trust led to an 11.3% reduction in adolescent pregnancies.^229^ Thus, there is a role for PHC-based systems in terms of steering multisectoral action at the local level, particularly when this action has a strong health focus. Establishing multisectoral actions and policies, including public-private collaboration, should include a whole-of-society and social determinants of health approach to support effective delivery of essential primary care services and non-health interventions along the resilience cycle.

Table 4 offers a summary of ideal scenarios of multisectoral action for resilient PHC, with complementary high-level policy actions.

**Table 4:** Ideal scenario and summary of policy options for multi-sectoral action towards resilient PHC.

Domain	Ideal scenario	High-level summary of policy options^a^
1. Intersectoral engagement	The intersectoral collaboration is embedded at both national and local levels, including a whole-of-society and social determinants of health approach, to support the effective delivery of essential primary care services and non-health interventions along the resilience cycle.	1. Create intersectoral entities to integrate health and non-health sectors in preparing and responding to external shocks (including early-warning systems).^206^^,^^230^^,^^231^ 2. Develop emergency plans with PHC and social determinants perspectives.^198^^,^^206^ 3. Identify primary care actions and non-health services in emergency plans.^203^^,^^231^^,^^232^ 4. Map and utilize non-health actors for primary care service provision during shocks (e.g., public security to reduce the impact of urban violence, using schools as shelters, etc.).^206^^,^^230^ 5. Support health workers with non-health sector services (e.g., childcare). 6. Leverage non-health workers and community members in emergencies.^146^^,^^233^ 7. Ensure basic services (water, energy, security) with backup mechanisms.^234^ 8. Harmonize local PHC-based systems response with the International Health Regulations.
2. Private for and not-for-profit health sector engagement	Institutionalized public-private collaborations across the resilience cycle to ensure primary care service provision following public health goals.	1. Establish regulatory instruments for public-private collaboration in service provision.^203^^,^^204^, ^206^, ^231^, ^232^, ^234^ 2. Convene private and public actors to sustain primary care services during shocks.^231^^,^^232^ 3. Assess private sector capacity to complement public sector surge capacity.^146^^,^^204^^,^^235^ 4. Implement accountability mechanisms with periodic public reports for transparency.^203^^,^^230^ 5. Count on an independent entity to oversee public-private primary care service provision.
3. Governance, production, and use of data	Efficient and permanent intersectoral communication systems that include data collection and analysis at all levels, as well as knowledge management for local decision making in the context of shocks.	1. Standardize data for sector sharing and empanelment, as well as multilateral collaborations in facing common shocks.^203^^,^^234^ 2. Regulate data sharing transparency and privacy. 3. Collect data from non-administrative sources (e.g., surveys).^231^ 4. Build intersectoral dashboards with key resilience indicators and ensure their use in decision-making.^230^^,^^231^ 5. Create risk and vulnerability maps using multisectoral data.^230^^,^^234^ 6. Build capacity to collect, analyse, and communicate data insights.
4. Environmental considerations	PHC-based systems comprehensively plan for health adaptation and mitigation to climate change, taking advantage of enabling conditions, considering key system vulnerabilities and promoting climate-resilient actions within the community.	1. Create climate health plans integrating territorial risk assessments.^230^^,^^231^ 2. Provide climate health information through primary care facilities.^236^ 3. Promote local climate-friendly actions via primary care activities.^236^ 4. Train health care providers in environmentally conscious PHC services.^236^ 5. Build and refurbish primary care facilities with a low-carbon emission development approach.

a Full policy options can be found in Supplementary Material 1.

##### Intersectoral engagement

Intersectoral collaboration must be embedded at both national and local levels to realize resilient PHC across LAC, incorporating a whole-of-society and social determinants of health approach to support the effective delivery of primary care services and relevant non-health interventions throughout the resilience cycle.^146^^,^^233^ This requires intersectoral collaboration to address the social determinants of health, planning, preparedness and response activities, and mapping non-health actors to support resilient PHC.

Coordinating non-health sectors, such as education and public security, ensure a comprehensive approach to PHC resilience.^206^, ^230^, ^231^, ^234^ For example, schools can play a role in health education and awareness, while public security forces, like the police and army, can assist in emergency response and logistics. Leveraging the roles of non-health workers and community members further strengthens this approach. Non-health workers can provide support services, such as transportation and communication, while community members can offer valuable insights and local knowledge. This collaborative effort ensures that all aspects of community health and well-being are addressed, leading to more resilient and responsive PHC-based systems.

Planning and preparedness are key to ensure that multi-sectoral responses, including primary care, are activated during emergencies. National and local plans should explicitly incorporate a PHC and social determinants of health perspective, addressing various risks such as hurricanes and mass casualty events. These plans should identify specific actions at the PHC level and establish networks to provide social protection support, such as financial security, food aid, and subsidies, ideally created both multi-sectoral and in partnership with communities.^198^^,^^203^^,^^206^^,^^231^^,^^232^ These efforts should interlink with social protection systems to ensure that basic human needs are met with health and social services at the local level during and after shocks. This includes mechanisms to ensure basic services like potable water, energy, and security.^234^ Panel 13 portraits how the city of Quito developed the Community Health Teams strategy during the COVID-19 pandemic.

Panel 13 **Quito’s Community Health Teams (CHT) during the COVID-19 pandemic.**

In response to the COVID-19 pandemic, the municipality of Quito, Ecuador, developed a strategy of CHTs.^237^ These teams shifted during the pandemic from reactive to proactive care and then post-pandemic to a preventive model that integrated social determinants of health with multisectoral interventions that mobilized other sectors of the municipal government.

Primary care services addressed social and environmental factors, supported by a sustained post-pandemic budget increase. A dynamic health workforce quickly learned and adapted to the new model of care, while digital platforms and telemedicine greatly improved access to services. However, challenges include the need for performance indicators to measure the impact of the intervention and model, limited resources, resistance from some communities that prefer curative care, cultural differences, and ensuring the safety of health workers in insecure areas. Sustained funding, impact indicators, community engagement, and multisectoral collaboration are essential to ensure long-term success of the CHT strategy.

Working collaboratively to identify, map, and leverage relevant public and private non-health actors and their capacities is essential to bolstering PHC resilience.^206^^,^^231^ This includes identifying and planning for the use of facilities like hotels, sports venues, shelters, schools, and warehouses to support PHC-based system PHC responses to crisis.^206^^,^^231^ Beyond infrastructure, non-health sector support, including childcare, financial relief, and occupational health support, is necessary for health workers to continue providing primary care services. By ensuring coordinated responses from the local to the global level, primary care response plans can be developed in consideration of the International Health Regulations and any other future related international agreements.

##### Private for and not-for-profit health sector engagement

Institutionalized public-private collaboration is a relevant governance policy across the resilience cycle to ensure the provision of primary care services in alignment with public health goals across LAC. In many contexts effectively engaging and strengthening collaborations with both private (for-profit and not-for-profit) health sectors is important to sustaining essential PHC services during emergencies. This requires regulatory instruments and public-private collaboration, capacity assessment and surge capacity, with accountability and oversight.

Regulatory instruments such as legislation, plans, and bylaws are necessary to ensure effective and sustainable collaboration.^203^^,^^204^^,^^206^^,^^231^, ^232^, ^234^ These instruments should define the roles, contracting-out rules, funding mechanisms, and oversight required for public-private collaboration in service provision. Convening both private and public actors is a key component of planning, responding, and learning together to maintain essential primary care services during shocks.^231^^,^^232^ This includes contracting-out and other types of public-private collaborations.

Assessing the capacity of the private sector, including available infrastructure and equipment, and understanding its complementarity with the public sector is important to enhance surge capacity.^146^^,^^204^^,^^235^ Accountability mechanisms, such as periodic public reports, should be established to provide a clear understanding of surge capacities and ensure financial transparency in public-private collaborations.^203^^,^^230^

Finally, an independent public entity should oversee and enforce the rules governing the provision of primary care services by both the public and private sectors. This entity would ensure that all parties adhere to the established regulations and maintain the quality and continuity of primary care services.

##### Governing data production and use

Data governance is essential for coordinating the various actors and data sources that contribute to a comprehensive understanding of community health and well-being. To strengthen data governance in LAC, efficient and permanent intersectoral communication systems must be created across the region, which include data collection and analysis at all levels, as well as knowledge management for local decision-making in the context of shocks. Strengthening the governance, production and uses of data to facilitate whole-of-government system resilience data sharing across sectors requires data standardization and sharing, intersectoral dashboards and risk mapping, underpinned by capacity building for effective data use.

Standardizing data, such as nominal IDs and electronic health records, is essential to facilitate sharing between sectors and improve coordination.^203^^,^^234^ Regulating data sharing, transparency and privacy between sectors ensures that sensitive information is protected while enabling effective collaboration.^203^^,^^234^ Collecting data from non-administrative sources, including surveys, civil society, and social media, provides a comprehensive view of situations and supports decision-making.^231^

Building intersectoral dashboards with key indicators across the resilience cycle at both national and local levels helps monitor and evaluate progress.^231^^,^^234^ Creating risk and vulnerability maps using multisectoral data informs action plans at the local level, addressing geographical and disadvantaged groups’ needs. These tools can help identify areas of concern and prioritizing interventions.

It is important to build capacity by creating and training teams at the national and local levels to collect, analyse, and communicate data insights with an intersectoral approach (Panel 14 describes Mexico City’s innovative health monitoring system during COVID-19). By adopting an intersectoral approach, these teams can integrate data from various sectors, providing a comprehensive view for decision-making in primary care, particularly during emergencies or shocks.^231^^,^^234^ This ensures that data is used effectively to inform policies and actions, fostering a collaborative environment where different sectors work together to enhance resilience and response capabilities. However, across the region, challenges such as data quality, system interoperability, and the need for health care workforce training impede effective implementation.^184^^,^^186^

Panel 14 **Navigating crisis: Mexico City’s innovative health monitoring system during COVID-19.**

During the early months of the COVID-19 pandemic, the Mexico City government faced the daunting task of managing hospital capacity and making timely decisions.^238^ To tackle this, they established a Health Council and developed a robust health monitoring system. This system centralized data from various sources, estimated key indicators, and provided a clear picture of the pandemic’s status. It became an essential tool for communication and policy guidance.

The system’s dashboard fostered collaboration among different sectors, including government ministries, non-health sectors like chambers of commerce, and the general population. This intersectoral cooperation helped reduce the burden on hospitals and improved decision-making processes.

Key lessons from this experience highlighted the importance of strong leadership, consensus on emergency assessment tools, actionable information, and adaptability to partner capacities. To enhance the system’s resilience, recommendations included operational flexibility, awareness of emerging needs, adaptability to new data and users, continuous communication, data transparency, and maintaining a skilled and motivated team.

The successful implementation of this monitoring system demonstrated how a shared framework could incentivize collaboration and provided valuable insights for strengthening preparedness and resilience in future emergencies.

##### Environmental considerations

Countries across LAC are vulnerable to the impact of climate change and climate-related disasters.^239^ PHC-based systems must comprehensively plan for climate change adaptation and mitigation addressing key system vulnerabilities and promoting climate-resilient and sustainability-driven action in primary care. This requires strong intersectoral collaboration, engaging sectors such as environment, agriculture, water, and disaster management, to develop coordinated and effective responses.

National and sub-national plans should be integrated into a multi-hazard framework for climate change adaptation.^137^^,^^231^ This involves conducting territorial risk assessments and evaluating the impact on the PHC-based system and service provision. By doing so, these plans can address various climate-related risks and ensure PHC resilience.^236^ For instance, for over a decade, climate-risk planning in Nicaragua has highlighted health risks due to rainfall and extreme events and has emphasized the importance of expanded health coverage, as well as climate and health early warning systems and planning.^240^ Primary care workers play a crucial role in this framework as trusted sources of climate and environmental information for their communities and can help communities understand the connections between climate change, environmental factors, and health and well-being.^236^ This dual role not only enhances community awareness but also strengthens the overall response to climate-related health impacts.

Providing primary care, like all health care, can be carbon-intensive, contributing to the drivers of climate change while also being required to respond to its impact on health and well-being.^241^^,^^242^ Advocacy and efforts are growing across LAC to make health systems more environmentally sustainable by promoting sustainable practices in facilities, community engagement in climate action, and climate-informed healthcare delivery.^44^^,^^243^^,^^244^ For example, recent efforts in Ecuador promote climate-resilient health systems through national surgical plans that emphasize emergency preparedness and aim to reduce the impacts of events like El Niño, as part of a strategic decision to ensure health systems are resilient, while also boosting the capacity to immediate environmental crises.^243^ Yet, action is needed to embed environmental sustainability and carbon-neutral services at the local level through initiatives such as waste reduction; water, sanitation and hygiene; air quality improvement; energy efficiency and clean energy adoption. The Smart Health Facilities Initiative of PAHO is another positive example, having promoted the retrofitting of over 100 healthcare facilities across the Caribbean to make them more energy-efficient, environmentally sustainable and resilient to disasters, particularly in the context of more active hurricane seasons.^245^ This can be translated into building and refurbishing primary care facilities to be more environmentally sustainable is a vital step that can leverage local and Indigenous knowledge to design spaces and processes that responsibly use local resources and respond to local climate needs, while protecting local and global environments.

#### Financing

Resilient PHC requires secure and robust financing primarily through public funds. To ensure continuity of essential PHC services in LAC, efforts are needed to enhance governance, effectively pool resources, and ensure flexible mechanisms for rapid resource allocation and payment during a crisis. Table 5 offers a summary of ideal scenarios of financing for resilient PHC, with complementary high-level policy actions.

**Table 5:** Ideal scenario and summary of policy options for financing resilient PHC.

Domain	Ideal scenario	High-level summary of policy options^a^
1. Financial governance	Rapid, transparent, sustainable and effective resource (re)allocation between national and local governments that facilitate a resilient and responsive PHC-based system for health emergencies.	1. Emergency financial decrees drafted ahead of time for targeted urgent PHC funding.^246^, ^247^, ^248^, ^249^ 2. Centralized fund management with local and intersectoral execution and extra resources for equity.^250^^,^^251^ 3. Conduct regular financial risk assessments for potential shocks.^235^ 4. Allocate dedicated emergency funds for local governments.^146^^,^^250^ 5. Implement flexible legal mechanisms for fund mobilization.^246^ 6. Track and monitor PHC resilience funds for accountability.^69^ 7. Coordinate financial dialogues between sectoral ministries.^139^^,^^250^^,^^252^
2. Revenue raising and pooling resources	Robust, coordinated financial mechanisms to ensure sufficient, readily accessible financing for prevention, preparedness, response and recovery to minimize volatility during external shocks.	1. Assure adequate funding sources to safeguard emergency funds.^253^ 2. Diversify funding sources through various health-related taxes.^253^^,^^254^ 3. Reduce dependency on vulnerable populations for funding.^235^^,^^246^ 4. Create sustainable emergency funds for resilience with local contributions.^255^ 5. Establish insurance mechanisms for collective emergency buffers.^256^, ^257^ 6. Accept funds from humanitarian and development organizations. 7. Implement special taxes for dedicated health resilience funding.^253^ 8. Enable efficient national-to-local government fund transfers.^146^ 9. Include budget provisions for resilience and emergency preparedness.^246^ 10. Determine essential health services and their costs during shocks.
3. Management and allocation of funds	Mechanisms that guarantee primary care has timely and reliable access to resources, mobilizing them swiftly during and after a shock to ensure readiness and responsiveness in emergencies.	1. Develop adaptable service packages for health crisis management.^34^^,^^253^ 2. Define rules for rebudgeting and modifying benefit packages.^246^ 3. Use forecast analyses to identify vulnerable populations. 4. Implement data-supported accountability systems for transparency.^246^^,^^254^ 5. Create guidelines for re-budgeting to improve resource efficiency.^240^ 6. Finance innovative community-driven proposals with flexible funding.^258^ 7. Ensure essential health services for all, including migrants.^259^
4. Purchasing and payment mechanisms	A system capable of providing prompt, adaptable financial support to primary care providers during crises, ensuring continuous delivery of essential and emergency response services.	1. Provide hazard pay, overtime compensation, and adjust payroll for temporary health care workers.^146^^,^^260^ 2. Use centralized purchasing agreements and procurement mechanisms for favourable rates and better pricing.^260^, ^261^, ^262^ 3. Implement electronic payment systems for timely compensation and international transactions.^34^^,^^263^ 4. Ensure legal protections, anti-fraud measures, and financial disincentives to prevent price gouging.^254^ 5. Ensure cost-effectiveness and conduct risk assessments for emergency product procurement. 6. Sign ex-ante contracts and diversify suppliers to strengthen supply-chain resilience. 7. Allow service purchases in other jurisdictions for portability and reimbursement. 8. Financial disincentives to ensure providers do not unjustifiably increase their prices during and after shocks.

a Full policy options can be found in Supplementary Material 1.

##### Financial governance

Financial governance is needed to achieve resilient PHC, and resilient health systems more broadly. Resilient PHC financing should be achieved through predominantly public government funds, by strengthening governance, efficiently pooling resources, and building adaptable mechanisms for swift resource (re)allocation and payment. Rapid, transparent, sustainable and effective resource (re)allocation between national units and ministries, and between national and local governments are necessary to facilitate a resilient and responsive PHC-based system for health emergencies. Establishing flexible financing mechanisms and implementing them rapidly and transparently enables swift mobilization and reallocation of funds dedicated to PHC, in turn, facilitating a rapid and effective response to health emergencies. Building sustainable emergency funds, at regional, national, and global levels can provide reliable financial sources, reducing dependency on ad-hoc international aid, and ensuring immediate access to PHC resources locally.

This requires legal frameworks to ensure adequate funds and emergency financial protocols, pre-established financial flows and preparedness mechanisms, centralized fund management and local execution, with accountability and monitoring.

Drafting emergency financial decrees ahead of time with clear protocols is essential to enable targeted funding for urgent primary care activities, such as community health outreach, vaccinations, and essential services. Regular financial risk assessments help determine potential shocks and identify the populations most likely to be affected. Dedicated funds for health emergencies allow local governments, based on their risk assessments, to quickly access and utilize resources when needed. Flexible legal mechanisms are also necessary to allow for the swift mobilization and reallocation of funds dedicated to PHC needs during emergencies. Panel 15 summarizes the financial protection experience in Belize during the COVID-19 pandemic.

Panel 15 **Financial protection in Belize during the COVID-19 pandemic.**

A case study conducted in Belize examines financial protection in the country exploring the role that PHC has in protecting households from financial burden.^264^

During the pandemic, while Belize had a modest increase in health spending in the first year of the pandemic, a later surge of cases forced the government to react, amending the Finance and Audit Reform Act to establish a contingency fund for future emergency responses. PHC during the pandemic played a key role in infection control, as well as risk communication, although its role could have been strengthened to maintain non-urgent or pandemic-related services. While this was particularly sensitive during the pandemic, the root causes of this issue are connected to pre-existing health system weaknesses, particularly human resource shortages. These factors resulted in the closure and/or suspension of some non-emergency PHC services, which affected the service care, as well as the out-of-pocket spending (OOP).

The issues that created more agreement among stakeholders on the potential causes for catastrophic and impoverishing health expenditures was the overreliance ono private sector services and the frequency of patients seeking cross-border care. A better understanding of OOP, forgone care and service availability will be crucial to cope with future emergencies in Belize and LAC more broadly.

Centralized fund management, combined with empowered local execution and extra resources, where possible, ensures that primary care resources reach communities effectively and equitably. This approach allows for a coordinated response while maintaining the flexibility needed to address local needs. Financial coordination mechanisms facilitate structured dialogues between financial officers of various sectoral ministries and the Ministry of Finance, ensuring a cohesive and comprehensive approach to managing health emergencies.

Tracking and monitoring systems for funds assigned for PHC resilience can increase accountability. These systems ensure that resources are used efficiently and transparently, providing oversight and enabling adjustments as needed. By maintaining robust monitoring mechanisms, stakeholders can ensure that funds are directed towards the most critical areas and that the impact of financial interventions is maximized.

##### Revenue raising and pooling resources

Robust, coordinated financial mechanisms are needed to ensure sufficient, readily accessible financing for prevention, preparedness, response and recovery to minimize volatility during external shocks. Across LAC, countries must strengthen legal frameworks that can be used to disburse adequate resilience funds as well as diversified funding sources to provide universal primary care services during public health emergencies. At the same time, it is important to encourage financial investments and budget allocations to PHC resilience in preparing for external shocks. This can be done through financial strategies and protections, emergency funds and insurance mechanisms, as well as efficient fund management.

To ensure financial resilience during emergencies, adequate funding sources, such as taxes, must be assured. Diversifying funding sources through various types of taxes, including health-related sin taxes and production taxes, can provide a steady stream of resources for resilience activities. Implementing special taxes or levies dedicated to health initiatives aimed at resilience can reduce the reliance on emergency resource mobilization when crises occur. Additionally, reducing the dependency on funding from vulnerable or directly affected populations, such as user fees, ensures that primary health care services remain freely available during crises.

Establishing an emergency fund to invest in resilience preparedness, response and recovery is essential, with additional contributions that might come from local governments through multilateral or national/subnational arrangements. The OECD recommends investing 1.4% of GDP in resilience to ensure adequate preparedness in their member countries.^30^ Insurance and re-insurance mechanisms across countries, which can be public, private or mixed, can provide a collective buffer against public health emergencies and support recovery strategies after a shock. Specific budgetary provisions for resilience and emergency preparedness within health and other relevant sectors are also necessary to ensure a comprehensive approach to managing emergencies. See Panel 15 for an example of Belize during the COVID-19 pandemic.

Effective revenue collection is needed for an efficient emergency response. Mechanisms to accept funds from humanitarian and development organizations efficiently can streamline the process of resource allocation, both to governments and non-governmental organizations. Additionally, determining essential health services, their costs, and potential additional costs due to shocks, including infrastructure, medicines, personnel, and transportation needs, should inform the adequate amount of funds to be collected.

##### Management and allocation of funds

Allocation is about knowing who gets what, when, how, and for what, ahead of a shock. Mechanisms are needed that guarantee primary care has timely and reliable transfer of resources swiftly during and after a shock to ensure readiness and responsiveness in emergencies. Building a strong public financial management system is important. This requires flexible resource allocation and planning, accountability and efficiency, and community engagement.

A service package for health crisis management should incorporate planning and forecasting to enable flexible resource allocation, adaptable to evolving needs during emergencies. Defining rules and procedures that allow for rebudgeting and modification of the benefit package as circumstances change is essential. Forecast analyses can help identify vulnerable populations and funding recipients based on shock needs, ensuring effective resource deployment.

Accountability systems supported by data can increase the transparency of fund transfers related to shock costs. Systems can be managed by independent governmental agencies or a leading agency with sufficiently detailed prior agreement on processes and responsible actors in preparedness, response and recovery phases. Guidelines for re-budgeting within and across sectors and administrative levels can reduce investment duplication and improve efficiency in resource allocation. This includes considerations such as military involvement in delivery and how to account for financial contributions to avoid duplicate funding.

Financing innovative community-driven proposals with flexible funding is key to effectively engaging the community in health crisis management. Ensuring that essential health services are available and accessible for all populations across various jurisdictions, including disaster-affected migrants, is also critical. This approach helps maintain continuity of care and supports the resilience of health systems during emergencies.

##### Purchasing and payment mechanisms

A resilient financing system is one that is capable of providing prompt, adaptable financial support to primary care providers during crises, ensuring continuous delivery of essential and emergency response services. Strengthening streamlined, flexible, transparent, and autonomous purchasing and payment mechanisms is needed to guarantee the efficient procurement of goods and services to boost resilience. This relies on compensation and workforce management, procurement and supply chains considerations, as well as service portability and financial measures.

Implementing hazard pay and overtime compensation mechanisms for health care workers during emergencies is essential to ensure their well-being and motivation. Payroll adjustments should allow for the hiring of temporary health care workers or mobile personnel, as well as task shifting to meet the increased demand during emergencies. Electronic payment systems can facilitate timely compensation for health care workers and suppliers, supporting international transactions when necessary.

Centralized, collective purchasing agreements can guarantee the procurement of goods and services at favourable rates or prices. Legal protections and anti-fraud measures are necessary to ensure a certain degree of autonomy for purchasers during and after crises. See Panel 16 for the regional pool procurement mechanism that PAHO Strategic Fund represents.

Panel 16 **PAHO Strategic Fund as a pooled procurement initiative for PHC resilience.**

The PAHO Strategic Fund provides support for countries in the LAC region, including during the COVID-19 pandemic, showing how resource optimization can enhance resilience in PHC-based systems.^265^ The Fund is a regional mechanism to improve access to essential medicines and health technologies through pooled procurement. By consolidating demand across multiple countries, the fund negotiates lower prices, reduces procurement inefficiencies, and ensures a more stable supply of critical health products.

This mechanism was fundamental to strengthening PHC by enhancing affordability and availability of key supplies, ensuring continuity of services and reducing vulnerabilities during health emergencies such as the COVID-19 pandemic. By improving supply chain resilience, countries can allocate resources more effectively toward expanding and sustaining PHC services, particularly for underserved populations.

Beyond its role in procurement, the Strategic Fund also supports countries with technical assistance to build capacity on supply chain management (including forecasting, and financial planning). By providing standardized pricing and long-term agreements with suppliers, the Fund mitigates market volatility and ensures equitable access to essential medicines. Its impact extends beyond crisis response, fostering greater self-sufficiency among participating nations.

Ensuring cost-effectiveness in procuring products and services is essential for a timely response. Risk assessments can strengthen supply-chain management during emergencies, and signing ex-ante contracts with suppliers for emergency needs can ensure preparedness. Centralized procurement mechanisms with predefined payment mechanisms, such as open contracting in Guatemala, the National Health Fund in Jamaica, and CENABAST in Chile, can achieve better pricing, including through revolving funds for pooled purchasing of emergency supplies. Diversifying suppliers reduces dependency on single sources and increases resilience in supply chains.

Allowing the purchase of health services in other jurisdictions enables service portability and reimbursement rules across systems. Financial disincentives and legal measures (e.g., price-gouging laws) should be in place to ensure providers do not unjustifiably increase their prices during and after shocks. These measures help maintain fairness and affordability in the provision of essential services during emergencies. Panel 16 analyses the regulation of prices paid to public and private providers in some LAC countries during the COVID-19 pandemic.

### Country prioritization cases

Acknowledging that the policy options included as part of the Commission’s recommendations might vary in their level of relevance and feasibility across countries of the region, the Commission conducted a prioritization exercise in two countries: Chile and the Dominican Republic. These cases show how differences in local priorities might shift the importance of each of these policy options in specific settings.

A staggered method was used to prioritize these policy options. First, an online survey was conducted in Chile to assess the relevance and feasibility of each one of the policy options developed by the Commission. This survey was shared with participants in a validation workshop that involved multiple stakeholders including the Ministry of Health (MoH) and other actors. These participants provided their perspectives on the main policy recommendations of the Commission and their relevance to Chile. A critical judgement was made in terms of what policy options have some level of implementation in each country. Finally, the prioritized list of policy options was validated through a consultation with MoH officials. For the Dominican Republic, the initial prioritization was made by commissioners with knowledge and experience working in the country and then validated with MoH staff of the country.

Supplementary Material 4 presents the results of the prioritization exercise that was conducted in Chile and the Dominican Republic, respectively. Both Chile and the Dominican Republic prioritized the development of emergency response plans to ensure PHC facilities remain operational during shocks, reflecting a shared emphasis on service continuity. Meanwhile, Chile highlighted the strategic use of PHC units as sentinel sites for early outbreak detection, whereas the Dominican Republic focused on strengthening interprofessional PHC teams to deliver collaborative, person-centred care. Both cases show how the Commission’s recommendation can be used in very different contexts, which can be seen by comparing the differences in the policy options that are prioritized in each one of the countries.

## Part IV: towards implementation

The Commission has proposed policies and actions that can be taken in LAC to strengthen PHC resilience. However, it is important to consider the implementation of such policies. Although this wasn’t an explicit objective of the Commission’s work, this part of the report provides an initial discussion of the main issues that emerge as critical to putting the recommendations into practice.

Moving towards implementation, resilient PHC across LAC depends on an understanding of the political economy in the region, a range of key enablers, and the development of strategies and capacities for effective monitoring and evaluation.

### The role of political economy in shaping resilient PHC

The policy environment for resilient PHC in LAC is increasingly shaped by a dynamic and evolving interplay of health priorities, political agendas, and socio-economic transitions. While national health policy priorities differ across countries, they are generally informed by a combination of epidemiological trends, demographic shifts, political commitments, and international frameworks such as the Sustainable Development Goals (SDGs). External actors, including bilateral donors, multilateral agencies, and global health initiatives, continue to influence national agendas through technical assistance, funding, and normative guidance. However, as countries seek greater autonomy and sustainability, the role of domestic and transnational political economy will become more central in shaping health policy decisions.

Looking ahead, the prioritization of PHC will be influenced by emerging health challenges such as ageing populations, rising rates of non-communicable diseases, mental health needs, rapid technological change, and climate-related health risks. Many of these trends speak to the need to prioritize the type of low-cost, integrated, patient-centred and resilient services that PHC systems can offer. Ministries of Health will need to move beyond reactive responses to crises and adopt more strategic, forward-looking approaches to policy development. This includes institutionalizing long-term planning mechanisms, strengthening public health intelligence, and building capacity for anticipatory governance. Structured priority-setting tools such as Health Technology Assessment (HTA), burden of disease studies, and Multi-Criteria Decision Analysis (MCDA) offer valuable frameworks for evidence-informed decision-making to support evidence-based decision-making.^266^ For example, Mexico’s General Health Council uses HTA to inform coverage decisions under public insurance schemes, while Chile has leveraged burden of disease studies to prioritize NCDs in PHC planning.^267^ These tools can help policymakers navigate trade-offs between cost, equity, and feasibility, especially in resource-constrained environments. A central challenge in many LAC countries is not the absence of technical knowledge or viable policy solutions, but the difficulty of mobilizing the political and institutional conditions necessary to advance and sustain reform. The political economy of health will play a decisive role in shaping the future of PHC policy implementation. Fiscal constraints, debt burdens, and competing priorities across sectors may limit the space for health investment, particularly in PHC, which has historically been underfunded. At the same time, political cycles and leadership transitions can disrupt policy continuity and shift attention away from long-term health system strengthening. Effective implementation requires navigating entrenched interests, building institutional capacity at national and subnational levels, and fostering public support through inclusive and transparent processes. Given the diversity of governance structures and health system arrangements across the region, there is no universal blueprint.^40^ However, a growing body of evidence underscores the importance of political incentives in shaping reform trajectories. Social contracts between citizens and political leaders can serve as powerful levers for reform, especially in the context of political transitions and shifting public demands. These periods often reconfigure sources of political legitimacy and create opportunities for governments to prioritize health. As shown in comparative research across nine countries, reforms tend to gain traction when new administrations seek to establish legitimacy through policy action or are ideologically aligned with equity-driven agendas.^111^

While technical advisory bodies and academic institutions will remain important contributors, broader stakeholder engagement including civil society, local governments and private sector actors will be essential to ensure that PHC policies are inclusive, responsive and grounded in community realities. Importantly, stakeholders such as CSOs, unions, and community groups, can help shape political incentives by exposing gaps in access, demanding accountability, and mobilizing electorates in favour of change.^111^ The Mamas del Rio program, implemented in the Peruvian Amazon, illustrates the effectiveness of aligning local institutions and community structures to enhance PHC delivery. By training CHWs and traditional birth attendants (TBAs), the program successfully leveraged existing local human resources to improve essential newborn care, particularly for in–home births, in remote, geographically isolated, and socioeconomically marginalized settings, which was sustained after COVID-19.^268^ The inclusion and training of CHWs and TBAs generated local buy-in and ownership, which reduced potential resistance and facilitated the continuity and resilience of care even amid external disruptions such as the COVID-19 pandemic. In addition, the program framed maternal and newborn health care as a shared community responsibility and a critical component of broader social equity and justice goals, taking an ideological shift towards community ownership and culturally adaptive care. Such strategies significantly mitigate political resistance and incentivise active engagement from communities traditionally marginalized in broader health system structures.

Another essential actor that could play a significant role in shaping PHC resilience and health-system transformation are unions representing health workers and public sector employees.^269^ Organized unions can be powerful players in building resilient systems by promoting better working conditions, adequate staffing, and long-term public investment. Unions have the capacity to mobilize constituencies, influence policy debates, and champion reforms, while also needs to be considered as possible opposition to some reforms. In Brazil, for example, union action has been instrumental in producing changes in the wellness and safety at work regulations, as well as the creation of health services and programs dedicated to healthcare workers in the Unified Health System, which are also very relevant during public health emergencies. In addition, medical associations play a particularly strong role in influencing health policies. During Uruguay’s structural reform of the mid-2000s, there were two groups of doctors: those working in the public sector and those working in the private sector. After years of dialogue, an agreement was reached to integrate both sectors, which was the solution endorsed by these two associations. This was crucial for the progress and implementation of the reform.^270^

Special emphasis should be placed on the political economy of digital health transformation. Integrating digital health into PHC systems in LAC presents extensive political, economic, regulatory, financial and socio-cultural challenges. Many countries in the region lack robust legal frameworks that would enable digital health, particularly telemedicine and electronic health records (EHRs). Outdated legislation, fragmented policy environments and inconsistent data protection regulations reduce public trust in digital health systems and hinder implementation. Without permanent laws or unified definitions, private and public sector providers face obstacles when rolling out digital solutions, and weak monitoring and oversight mechanisms remain in place for digital services.^271^ The persistent digital divide also remains a critical barrier, exacerbated by poor infrastructure, low internet connectivity, and unequal access to electricity, particularly among rural, remote, and marginalised populations. Many health facilities still lack basic equipment and reliable internet services, making it difficult to equitably scale digital health initiatives, which requires the commitment of actors outside the health sector.^184^ A shortage of professionals skilled in digital health is a major problem, with most health workers concentrated in urban centres and rural areas consequently underserved.^272^ Workers and professional associations are key stakeholders in the development of such policies. Furthermore, integration across health data platforms and systems is a major challenge. Weaknesses in data storage, management and interoperability — often accompanied by an absence of standardised protocols — undermine care continuity and impede population health management, particularly for mobile and vulnerable populations. Data sharing among professionals is also limited by technology gaps and reluctance stemming from privacy concerns.^184^ Finally, deep-seated cultural norms, distrust of digital tools and low digital literacy levels in certain populations can hinder the adoption of digital health solutions. Programmes are often not designed with cultural relevance in mind, leading to resistance or limited engagement. Inequalities in digital access further exacerbate health disparities, perpetuating a two-tier system in which marginalised groups encounter obstacles in accessing care and information.^184^ Overcoming these obstacles necessitates comprehensive reforms, sustained investment, multi-level capacity building and trust-building initiatives.

Building resilience will therefore require not only technical solutions but also leadership and political commitment. Political champions, whether at the national, regional, or municipal level, can drive reform, mobilize resources, and sustain momentum. Governments, civil society, and international partners all have critical roles to play in creating the enabling environment needed to move from policy to practice and to build more equitable and resilient health systems across the region. Proactive stakeholder engagement will be needed to drive resilience in PHC, and governments must evolve to meet the demands of a more participatory and accountable policy environment. While some countries have established formal platforms for civil society and professional associations to contribute to health planning, many still face challenges in ensuring meaningful participation, especially from marginalized and underserved populations. Future efforts must focus on strengthening inclusive governance, enhancing transparency, and institutionalizing mechanisms for community input throughout the policy cycle, from design to implementation and evaluation.

In sum, PHC reforms are always political, and governments need to be prepared to manage the complex political economy of reform with sophisticated management strategies. Ensuring PHC resilience will also require renewed investment in effective institutions, inclusive policy processes, and a strengthened social contract that fosters mutual trust and civic engagement.

### Enablers for implementing resilient PHC

A range of enabling factors will be critical to the successful implementation and long-term sustainability of resilient PHC in LAC. As countries confront increasingly complex health and social challenges, these enablers must not only support current reforms but also anticipate future needs and opportunities.

One key strategy is the use of pilot programs to test and refine interventions before scaling them nationally. Piloting allows for context-specific adaptation, stakeholder engagement, and evidence generation. In the future, pilot initiatives can serve as innovation laboratories, helping countries experiment with new service delivery models, digital tools, and financing mechanisms in controlled environments. These pilots can also foster a culture of learning and iteration within health systems, which is essential for resilience.

Digital health innovations will play a transformative role in the coming years. Telemedicine, mobile health applications, electronic health records, and artificial intelligence can extend the reach of PHC, improve continuity of care, and enhance decision-making. However, realizing this potential will require investments in digital infrastructure, data governance, cybersecurity, and digital literacy for both providers and patients, as well as developing robust policies to guide the development of digital technologies in health. Future digital strategies should ensure that equity is prioritized so that rural, Indigenous, and low-income populations are not left behind in the digital transition.

Partnerships will remain central to implementation success. Cross-sector collaboration, linking health with education, housing, environment, and social protection, can address upstream determinants of health and create synergies across policy domains. Public-private partnerships can bring in technical expertise, innovation, and financing, while regional cooperation can facilitate knowledge exchange, joint procurement, and coordinated responses to shared challenges such as pandemics or climate events. In the future, these partnerships may evolve into more formalized networks or platforms that support regional resilience and solidarity.

Academic and research institutions will continue to play an essential role in generating evidence, evaluating interventions, and critically, training the next generation of PHC professionals. The policy reform options identified above call for substantial changes in the skillsets and orientation of PHC professionals that will in turn require major changes to training curricular.

### Research gaps and opportunities for regional learning and scale-up

Gaps persist in both our understanding of resilient PHC and our ability to monitor and measure PHC capacities and performance in LAC.^74^ These gaps challenge resilient PHC by undermining our ability to understand not only the impacts of efforts to strengthen resilience, but also what works (or not) and why (Table 6). We found that the literature on resilient PHC often discusses general interventions like improving patient referrals and reducing emergency room crowding, with specific acute shocks such as pandemics, conflict settings, and climate-related shocks being the most studied. Overall, studies of PHC resilience tend to focus on high-income countries like the US, Canada, Australia, and Germany, which may not fully reflect LAC contexts. While there is literature from Brazil, Mexico, Colombia, Chile, Guatemala, Peru, Argentina, Uruguay, Costa Rica, Jamaica, and El Salvador; geographic representation is uneven across the region and sub-regions. There is a gap in integrating PHC and health-system resilience into a combined framework, with resilience often viewed abstractly or reactively. Most literature focuses on the shock and alert, and response phases, with few studies on preparedness, prevention, and recovery, highlighting the need for research on the full resilience cycle and the importance of planning to reduce the impact of shocks on health systems. Supplementary Material 3 presents an exploration of these gaps by domain and sub-domain.

**Table 6:** Research gaps by domain.

Domain	Research gaps
Integrated health services and public health functions	• **Limited evidence on inclusive PHC models:** Scarce research on equity-focused, adaptable models in diverse LAC settings. • **Weak evidence on coordination and integration:** Limited assessments on how coordination impacts PHC performance, resilience, and patient outcomes. Documentation on referral pathways, feedback loops, and integration with specialized care remains scarce. • **Gaps in tracking and evaluation:** Key system outcomes like early detection, service continuity, and long-term intervention effects are rarely tracked or evaluated. Most studies rely on short-term observations with inconsistent findings. • **Fragmented research on public health functions:** Fragmented strategies for integrating surveillance, promotion, and protection into PHC. Existing studies focus more on crisis response than sustaining essential services. • **Context-specific and small-scale initiatives:** Many interventions remain isolated, lack comparative evaluations, and are often designed for high-income settings. • **Need for institutionalization and preparedness:** Research is needed on embedding service reorganization strategies into routine primary care to enhance system responsiveness during health shocks.
Community empowerment and participation	• **Lack of comparative and longitudinal evaluations:** Few studies compare CHW and peer-led models across countries or over time, limiting understanding long-term integration and effectiveness in primary care. • **Fragmented and localized research:** Most studies are descriptive, context-specific, and small in scale, with highly localized cultural adaptations that lack frameworks for evaluation, replication, or system-wide integration. • **Under-evaluated implementation and resilience impact:** While CHW programs are widely endorsed, their implementation mechanisms, role in PHC resilience, and connection to intercultural approaches during health shocks remain insufficiently explored. • **Gaps in communication strategies:** Context-specific, evaluated communication strategies for primary care during emergencies are lacking. Limited evaluation and under-theorization of messaging tailored to marginalized groups, strategies to mitigate misinformation, and trust-building. • **Limited evidence on transparency and accountability:** Their direct impact and integration into health governance remain underexplored.
Multi-sectoral action	• **Descriptive and short-term studies:** Research on private sector engagement and intersectoral collaboration in PHC is mostly descriptive, often in fragile settings, with little evaluation of long-term integration, financial transparency, or impact. • **Underexplored role of private actors:** The private sector’s influence on PHC resilience during shocks remains largely unevaluated, with limited assessment of its contributions to service continuity, system responsiveness, and equity, as well as the regulations required. • **Gaps in governance and implementation evaluation:** Studies often focus on governance processes rather than concrete impacts on service delivery, shock preparedness, or recovery. Implementation challenges—such as lack of trust, unclear mandates, and coordination gaps— are underexamined. • **Environmental considerations as an emerging but weakly assessed area:** Despite its conceptual link to PHC resilience, interventions for environmentally sustainable healthcare lack robust evaluation, operational frameworks, and follow-up data, particularly in LMICs.
Financing	• **Fragmented and reactive financial resilience research:** Studies on financial coordination, resource allocation, and funding mechanisms are highly heterogeneous, with limited comparative impact assessments and a predominant focus on reactive rather than proactive resilience strategies. • **Sparse literature on funding and PHC linkage:** Research on crisis funding mainly covers humanitarian aid, insurance expansion, and donor support, with little connection to PHC structures, long-term resilience, or sustainability beyond emergencies. • **Gaps in financial governance and regulatory mechanisms:** Legal and regulatory frameworks for crisis response are documented but rarely analysed in relation to PHC responsiveness, intersectoral coordination, or funding agility. • **Inconsistent evaluation of financial protection and equity:** While financial modifications improve service utilization and equity, studies lack standardized outcome measures and comparative analyses across different crisis contexts. • **Limited research on payment reforms and digital integration:** Payment reform studies focus narrowly on short-term efficiency, neglecting their role in PHC adaptability, workforce retention, and resilience under different types of shocks.

Despite persistent challenges, the diversity of PHC experiences in LAC offers a strong foundation for shared learning and innovation. Countries across the region are actively implementing models such as integrated service delivery, digital health solutions, and community-based care. These efforts, while context-specific, generate valuable insights that can inform broader strategies to enhance PHC resilience.

Regional collaboration is essential to accelerate progress. Networks and platforms can facilitate peer-to-peer learning, joint capacity building, and coordinated technical assistance. Mechanisms such as communities of practice, regional observatories, and virtual learning hubs enable countries to share implementation experiences, policy innovations, and operational tools. These platforms also support the contextual adaptation of global best practices, ensuring that scale-up efforts are relevant and sustainable.

Regional institutions and development partners have a critical role in supporting the scale-up of effective PHC models. Investments in shared digital infrastructure, harmonised monitoring frameworks, and pooled procurement mechanisms can generate efficiencies and improve system performance. The development and adoption of standardized indicators for PHC performance and resilience are particularly important to enable benchmarking, promote accountability, and guide targeted support. Collaborative research initiatives can also help generate evidence on emerging priorities, including climate-resilient infrastructure, digital equity, and workforce transformation. In parallel, there is growing momentum behind regional financing mechanisms that promote both efficiency and equity. Joint procurement arrangements, regional stockpiles, and pooled funding models can improve access to essential medicines, diagnostics, and technologies, while also helping buffer national systems against global supply chain disruptions.

Ultimately, regional learning must serve a broader goal: building collective resilience. Shared challenges such as migration, climate change, and emerging health threats demand coordinated responses that transcend national borders. Strengthening regional solidarity, aligning regulatory frameworks, and investing in institutional capacity will be essential to ensure that all countries in LAC can benefit from the region’s collective expertise, experience, and innovation.

### Diagnosing, monitoring and evaluation for resilient PHC

To fill these research gaps and enhance our understanding of resilient PHC, there is urgent need to develop approaches, strategies, and tools that better enable policymakers, implementers and communities to define and measure success.

**Fig. 13:**
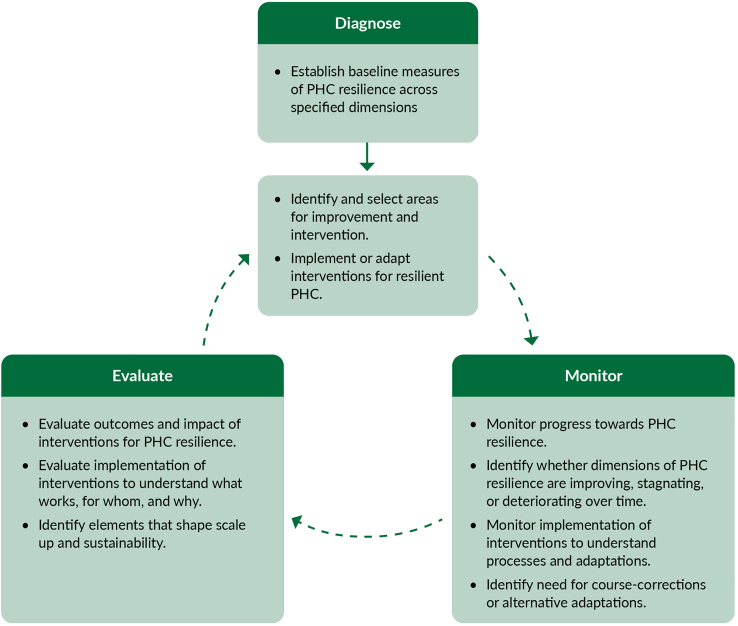
**Diagnosing, monitoring, and evaluating resilient PHC**.

Three key areas for action are strengthening how we diagnose, monitor, and evaluate PHC resilience in LAC (Fig. 13). Diagnosing PHC allows for a deeper understanding of resilience across specified dimensions (for example resources, capacities, policies or programs, to name a few) and goal or priority setting for implementation of interventions for resilient PHC. Monitoring allows for tracking progress towards PHC resilience and whether dimensions of PHC resilience are improving stagnating or deteriorating over time. Evaluation ensures that outcomes and impacts are articulated—including the processes, experiences, and adaptations that shape how interventions are implemented.

Importantly, data collection and use must be well regulated, with adequate privacy features and participatory governance, including before, during and after shocks. In these scenarios, striking a balance between public health need and individual privacy involves adhering to principles such as data minimization (limiting collection to what is necessary for the specific public health need), data retention (ensuring data is used and retained only as long as required), data protection (protect collected data from unauthorized access, use, or disclosure), data use and disclosure (use collected data only for the stated public health purpose), and transparency and accountability, especially to the ones providing the data.^273^^,^^274^

Currently, there is no comprehensive and contextualized approach for diagnosing, monitoring and evaluating resilient PHC across LAC. Efforts are needed to develop comprehensive data collection approaches to ensure data relevant to PHC resilience is available and interoperable with existing systems; propose and test key concepts and indicators relevant to PHC resilience in LAC; and develop, apply, and refine common monitoring tools to track progress, identify gaps, and make informed decisions towards resilient PHC.

## Conclusion

Our report highlights the urgency of developing resilient PHC-based systems throughout LAC. It is not a question of if, but when, as shocks are already hitting countries and health systems in the region and will continue in the future. We know that these shocks exacerbate existing crises and inequities, requiring far-reaching action to ensure that people have access to needed services where and when they need it, even during emergencies. In a context of rising geopolitical hostilities, increasing scepticism about science, escalating threats to public health gains, and the growing likelihood of serious and far-reaching emergencies, the stakes have never been higher, and the potential costs of inaction threaten millions of lives and economies across the region.

Given this high cost of inaction, the Commission emphasizes that resilience must be considered as a cornerstone of PHC and makes recommendations that will strengthen integrated services, essential public health functions, community empowerment and trust, multisectoral action, and financing, linked by the need for continued commitment and progress toward universal health and PHC-based systems in LAC. These recommendations mutually reinforce health coverage and resilience by ensuring that everyone can access and receive the health services they need, in their community, when they need it, without financial hardship, along the resilience cycle: before, during, and after shocks. There is no trade-off between the development of PHC-based systems and health system resilience; in fact, they are synergistic and mutually reinforcing.

While the challenges and threats of the 21st century are immense, there is indeed much to be optimistic about: building on the substantial gains in health and well-being in LAC over the past century shows that the region is capable of profound change and innovation. Countries must continue in this spirit and invest in resilient PHC, an effective investment that will pay dividends to protect people and economies from future shocks, whether local, national or global. The time to act is now, because as we learn time and again, emergencies will not wait for us.

## Contributors

Conceptualisation: CAH, EB, MVU, NH, SB, MCC, AM. Data curation: CM, AA, NA, EA, MG, CZ. Formal analysis: all authors. Funding acquisition: CAH, EB, MVU, NH. Project administration: CAH, EB, MVU, NH. Validation: CM, AA, MG, CAH, EB, MVU, NH, SB, MCC. Writing: VH, CAH, MVU, NH, SB, MCC, EB. Review & editing: all authors.

## Editorial note

The Lancet Group takes a neutral position with respect to territorial claims in published maps and institutional affiliations.

## Declaration of interests

The World Bank and PAHO funded travel and accommodation for all commissioners who participated in the two in-person meetings held by the Commission in Washington, DC (United States) and Panama City (Panama). AA, CM, MG, PG and SB had a short-term individual consultancy arrangement with the World Bank and EA and CZ were hired as PAHO consultants to conduct research work related to the Commission. FG received reimbursement of travel costs with speaking engagements in official capacity in conferences by the World Bank, Inter-American Development Bank, Mediano Sanita (Italy), European Patients Forum, European Association of Value Based Healthcare, Bertelsmann Foundation Germany, European Centre for Social Welfare Policy Austria, Portuguese Society for Internal Medicine, General Directorate for Healthcare—Portugal, Cascais International Health Forum, Global Health Exhibition Saudi Arabia, and the Quest Network/Lancet Global Health People’s Voice Survey. JH endowed research chair and support for the Bolivia case study from the St Mary’s Hospital Foundation in Canada. All other authors declared no conflict of interest.

## References

[1] Pan American Health Organization (2024).

[2] OECD/The World Bank (2023).

[3] Atun R., de Andrade L.O., Almeida G. (2015). Health-system reform and universal health coverage in Latin America. Lancet.

[4] Frenk J., Gonzalez-Block M.A. (1992). Primary care and reform of health systems: a framework for the analysis of Latin American experiences. Health Serv Manage Res.

[5] Gilardino R.E., Valanzasca P., Rifkin S.B. (2022). Has Latin America achieved universal health coverage yet? Lessons from four countries. Arch Public Health.

[6] World Health Organization, UNICEF (2018).

[7] Pan American Health Organization (2022). https://www.paho.org/en/stories/advancing-towards-universal-health-latin-america-and-caribbean-lessons-covid-19-pandemic.

[8] World Health Organization (2010).

[9] World Health Organization (2020). Pulse survey on continuity of essential health services during the COVID-19 pandemic.

[10] Pan American Health Organization (2020). The Essential Public Health Functions in the Americas: A Renewal for the 21st Century: Conceptual Framework and Description.

[11] World Health Organization (2024). Implementing the primary health care approach.

[12] World Bank (2022).

[13] Pan American Health Organization (2014). CD53.R14 - Strategy for Universal Access to Health and Universal Health Coverage.

[14] World Health Organization (1978).

[15] Maceira D., Quintero R.E.P., Suarez P., Pena Pena L.V. (2024). Primary health care as a tool to promote equity and sustainability; a review of Latin American and Caribbean literature. Int J Equity Health.

[16] de Andrade L.O., Pellegrini Filho A., Solar O. (2015). Social determinants of health, universal health coverage, and sustainable development: case studies from Latin American countries. Lancet.

[17] Abramo L., Cecchini S., Ullmann H. (2020). Addressing health inequalities in Latin America: the role of social protection. Cien Saude Colet.

[18] World Health Organization, World Bank (2023).

[19] Roberti J., Leslie H.H., Doubova S.V. (2024). Inequalities in health system coverage and quality: a cross-sectional survey of four Latin American countries. Lancet Glob Health.

[20] Houghton N., Bascolo E., Del Riego A. (2020). Socioeconomic inequalities in access barriers to seeking health services in four Latin American countries. Rev Panam Salud Publica.

[21] United Nations Office for Disaster Risk Reduction (2023).

[22] Pan American Health Organization (2025).

[23] Haldane V., Ong S.E., Chuah F.L., Legido-Quigley H. (2017). Health systems resilience: meaningful construct or catchphrase?. Lancet.

[24] COVID-19 Excess Mortality Collaborators (2022). Estimating excess mortality due to the COVID-19 pandemic: a systematic analysis of COVID-19-related mortality, 2020-21. Lancet.

[25] Herrera C.A., Veillard J.H.M., Feune de Colombi N., Neelsen S., Anderson G., Ward K. (2022).

[26] UNDP (2023).

[27] Thomas S., Sagan A., Larkin J., Cylus J., Figueras J., Karanikolos M. (2020).

[28] Turenne C.P., Gautier L., Degroote S., Guillard E., Chabrol F., Ridde V. (2019). Conceptual analysis of health systems resilience: a scoping review. Soc Sci Med.

[29] World Health Organization (2022).

[30] OECD (2023).

[31] Jatobá A., de Castro Nunes P., de Carvalho P. (2023). A framework to assess potential health system resilience using fuzzy logic. Rev Panam Salud Publica.

[32] Massuda A., Hone T., Leles F.A.G., de Castro M.C., Atun R. (2018). The Brazilian health system at crossroads: progress, crisis and resilience. BMJ Glob Health.

[33] Herrera C.A., Bascolo E., Villar-Uribe M., Houghton N., Massuda A., Bennett S. (2023). The world Bank - PAHO lancet regional health Americas commission on primary health care and resilience in Latin America and the Caribbean. Lancet Reg Health Am.

[34] Hanson K., Brikci N., Erlangga D. (2022). The Lancet Global Health Commission on financing primary health care: putting people at the centre. Lancet Glob Health.

[35] Palmeiro Y., Plaza Reneses T., Velenyi E., Herrera C.A., OECD, World Bank (2023). Health at a glance: latin America and the Caribbean 2023.

[36] LAC-INFORM (2020).

[37] Wickramaarachchi T., Scott N., Villalobos Dintran P., Gonzalez-Samano M., Villar Uribe M. (2025). The cost of inaction to strengthen the resilience of primary health care in Latin America and the Caribbean: a modelling study. Lancet Reg Health Am.

[38] Savedoff W.D., Bernal P., Distrutti M., Goyeneche L., Bernal C. (2022).

[39] Climate Investment Funds, World Bank (2024).

[40] OECD (2022).

[41] (2025). World Health Organization. Global Health Observatory. https://www.who.int/data/gho/data/indicators/indicator-details/GHO/life-expectancy-at-birth-(years).

[42] International Monetary Fund (2025). World Economic Outlook Database. https://www.imf.org/en/Publications/WEO/weo-database/2019/October/download-entire-database.

[43] OECD (2020).

[44] Vargas C, Gomez-Valencia M, Gonzalez-Perez MA (2022). Climate-resilient and regenerative futures for Latin America and the Caribbean. Futures.

[45] Romanello M., Walawender M., Hsu S.C. (2024). The 2024 report of the lancet Countdown on health and climate change: facing record-breaking threats from delayed action. Lancet.

[46] Harris-Glenville F., Cloos P. (2024). “I think they should give primary health care a little more priority”. The primary health care in Caribbean SIDS: what can be said about adaptation to the changing climate? The case of Dominica- a qualitative study. BMC Prim Care.

[47] Barcante J.M.P., Cherem J. (2025). The growing challenge of arboviruses in Latin America: dengue and oropouche in focus. PLoS Negl Trop Dis.

[48] Laserna A., Barahona-Correa J., Baquero L., Castaneda-Cardona C., Rosselli D. (2018). Economic impact of dengue fever in Latin America and the Caribbean: a systematic review. Rev Panam Salud Publica.

[49] Pan American Health Organization (2024). https://www.paho.org/en/news/20-6-2024-despite-record-dengue-cases-latin-america-and-caribbean-maintain-low-fatality-rate.

[50] Oliveira Roster K., Martinelli T., Connaughton C., Santillana M., Rodrigues F.A. (2024). Impact of the COVID-19 pandemic on dengue in Brazil: interrupted time series analysis of changes in surveillance and transmission. PLoS Negl Trop Dis.

[51] Dantes H.G., Manrique-Saide P., Vazquez-Prokopec G. (2020). Prevention and control of aedes transmitted infections in the post-pandemic scenario of COVID-19: challenges and opportunities for the region of the americas. Mem Inst Oswaldo Cruz.

[52] OCHA, UNDRR (2023). https://www.undrr.org/media/89900/download?startDownload=20250302.

[53] (2024). What happens when climate change and the mental-health crisis collide?. Nature.

[54] Castro M., Ponmattam J., FitzGerald E.A. (2025). Shocks and health care in Latin America and the Caribbean. Front Public Health.

[55] Seneviratne S.I., Zhang X., Adnan M., Masson-Delmotte V., Zhai P., Pirani A. (2021). Climate change 2021: the physical science basis contribution of working group I to the sixth assessment report of the intergovernmental panel on climate change.

[56] Vitriol V., Minoletti A., Alvarado R., Sierralta P., Cancino A. (2014). Respuesta de los centros de atención primaria en salud mental después del terremoto y tsunami del 2010 en la Región del Maule. Rev Med Chile.

[57] Novick G. (2017). Health care organization and delivery in Argentina: a case of fragmentation, inefficiency and inequality. Global Policy.

[58] Internal Displacement Monitoring Centre, Global Internal Displacement Database (2023). https://www.internal-displacement.org/database/displacement-data/.

[59] Pan American Health Organization (2011). Lessons to be learned for the next massive sudden-onset disaster.

[60] Sripad P., Casseus A., Kennedy S. (2021). “Eternally restarting” or “a branch line of continuity”? Exploring consequences of external shocks on community health systems in Haiti. J Glob Health.

[61] Genoni M.E., Muller M., Ishizawa Escudero O.A., Casabonne U. (2017). http://documents.worldbank.org/curated/en/873561513576965980.

[62] (2020). Index for Risk Management for Latin America and the Caribbean: LAC-INFORM 2020 Update.

[63] Cardona O.D., Aalst M.K.V., Birkmann J., Field C.B., Barros V., Stocker T.F. (2012). Managing the risks of extreme events and disasters to advance climate change adaptation A special report of working groups I and II of the intergovernmental panel on climate change (IPCC).

[64] UNDP (2019). LAC-INFORM 2020 update.

[65] Baez J., Caruso G., Mueller V., Niu C. (2017). Droughts augment youth migration in northern Latin America and the Caribbean. Clim Change.

[66] Santos de Lima L., Silva F.E.O.E., Dorio Anastácio P.R. (2024). Severe droughts reduce river navigability and isolate communities in the Brazilian Amazon. Commun Earth Environ.

[67] Msemburi W., Karlinsky A., Knutson V., Aleshin-Guendel S., Chatterji S., Wakefield J. (2023). The WHO estimates of excess mortality associated with the COVID-19 pandemic. Nature.

[68] PAHO, La Soufrière Volcano (2021). https://www.paho.org/sites/default/files/2021-08/ECC%20VCT%20Soufriere%20volcano%20SitRep%2034%20-%2029%20July%202021%20updated.pdf.

[69] Mora-Garcia C.A., Pearson A.A., Prado A.M. (2024). Maintaining essential health services during a pandemic: lessons from Costa Rica’s COVID-19 response. BMJ Glob Health.

[70] How did Costa Rica respond to the COVID-19 pandemic?: exemplars in Global Health. https://www.exemplars.health/emerging-topics/ecr/costa-rica/how-did-costa-rica-respond-to-the-covid-19-pandemic.

[71] Pan American Health Organization (2025). https://www.paho.org/en/tablero-indicadores-aps/dashboard-indicators-health-systems-based-primary-health-care#guideindicators.

[72] World Health Organization (2025). https://apps.who.int/nha/database/ViewData/Indicators/en.

[73] International Labor Organization (2023).

[74] Bernal P., Garcia M, Castro Vargas S., Perez-Cuevas R., Bauhoff S. (2025).

[75] World Health Organization (2021). United Nations Children’s Fund (UNICEF). Operational framework for primary health care: transforming vision into action.

[76] World Health Organization (2018). Declaration of Astana: Global Conference on Primary Health Care.

[77] Houghton N, Báscolo E, Jarboe R, Fitzgerald J (2025). Essential public health functions for primary health care resilience in Latin America and the Caribbean: a regional assessment.. Lancet Reg Health Am.

[78] Pan American Health Organization (2024).

[79] (IDB) I-ADB (2025). https://www.iadb.org/en/news/complexities-inequality-latin-america-and-caribbean.

[80] McNellan C.R., Dansereau E., Wallace M.C.G. (2019). Antenatal care as a means to increase participation in the continuum of maternal and child healthcare: an analysis of the poorest regions of four Mesoamerican countries. BMC Pregnancy Childbirth.

[81] Kamath A.M., Johanns C.K., Thom M.G. (2020). Assessing multidimensional care coverage for pre-eclampsia in the era of universal health coverage: a pre-post evaluation of the Salud Mesoamerica Initiative. Int J Gynaecol Obstet.

[82] Pan American Health Organization (2021).

[83] Costa J.C., Mujica O.J., Gatica-Dominguez G. (2022). Inequalities in the health, nutrition, and wellbeing of Afrodescendant women and children: a cross-sectional analysis of ten Latin American and Caribbean countries. Lancet Reg Health Am.

[84] Samuel J., Batzin B., Medina R. (2024). Indigenous-led struggles for health justice in the context of the climate emergency: insights from Guatemala. BMJ Glob Health.

[85] Herrera C.A., Juarez-Ramirez C., Reyes-Morales H. (2023). COVID-19 disruption to routine health care services: how 8 Latin American and Caribbean countries responded. Health Aff (Millwood).

[86] Colombara D.V., Hernandez B., Schaefer A. (2016). Institutional delivery and satisfaction among Indigenous and poor women in Guatemala, Mexico, and Panama. PLoS One.

[87] Fuene de Colombi N, Blumenfeld A, Saez Molina N, et al. Building resilient and inclusive primary health systems: addressing the needs of LGBTI+ people in Latin America [Forthcoming].

[88] Torres J.L., Gonçalves G.P., Pinho A.A., MHDN S. (2021). The Brazilian LGBT+ health survey: methodology and descriptive results. Cad Saude Publica.

[89] Socias M.E., Marshall B.D., Aristegui I. (2014). Factors associated with healthcare avoidance among transgender women in Argentina. Int J Equity Health.

[90] World Bank (2020). https://www.worldbank.org/en/news/feature/2020/05/15/estigma-cuarentena-covid-lgbti.

[91] Montenegro R.A., Stephens C. (2006). Indigenous health in Latin America and the Caribbean. Lancet.

[92] Ferdinand A., Lambert M., Trad L., Pedrana L., Paradies Y., Kelaher M. (2020). Indigenous engagement in health: lessons from Brazil, Chile, Australia and New Zealand. Int J Equity Health.

[93] United Nations Office for Disaster Risk Reduction (2024).

[94] Gomez-Dantes O., Flamand L., Cerecero-Garcia D., Morales-Vazquez M., Servan-Mori E. (2023). Origin, impacts, and potential solutions to the fragmentation of the Mexican health system: a consultation with key actors. Health Res Policy Syst.

[95] Bossert T., Blanchet N., Sheetz S., Pinto D., Cali J., Rp C. (2014).

[96] Palacios A., Espinola N., Rojas-Roque C. (2020). Need and inequality in the use of health care services in a fragmented and decentralized health system: evidence for Argentina. Int J Equity Health.

[97] Fitzgerald J., Bascolo E., de Almeida G.R., Houghton N., Jarboe R., Issa J. (2024). Addressing migrant-specific barriers to accessing health services through primary health care in host countries in Latin American and the Caribbean. Lancet Reg Health Am.

[98] Pinto H.A., Cortes S.M.V. (2022). What made the mais medicos (more doctors) program possible?. Cien Saude Colet.

[99] Pena S., Ramirez J., Becerra C., Carabantes J., Arteaga O. (2010). The Chilean rural practitioner programme: a multidimensional strategy to attract and retain doctors in rural areas. Bull World Health Organ.

[100] Pan American Health Organization (2022). https://www.paho.org/en/news/27-5-2022-americas-has-shortfall-600000-health-professionals-affecting-access-health-rural-and.

[101] Rolle Sands S., Ingraham K., Salami B.O. (2020). Caribbean nurse migration-a scoping review. Hum Resour Health.

[102] Bitton A., Ratcliffe H.L., Veillard J.H. (2017). Primary health care as a foundation for strengthening health systems in Low- and middle-income countries. J Gen Intern Med.

[103] Rajan D., Jakab M., Schmets G. (2024). Political economy dichotomy in primary health care: bridging the gap between reality and necessity. Lancet Reg Health Eur.

[104] World Health Organization (2025).

[105] Báscolo E, Houghton N, Bennett S (2023). Political economy of primary health care resilience in Latin America and the Caribbean: insights from the WB/PAHO Commission recommendations.. Lancet Reg Health Am.

[106] Acemoglu D., Robinson J.A. (2013).

[107] Campos P.A., Reich M.R. (2019). Political analysis for health policy implementation. Health Syst Reform.

[108] Fox A.M., Reich M.R. (2015). The politics of universal health coverage in Low- and middle-income countries: a framework for evaluation and action. J Health Polit Policy Law.

[109] Sparkes S.P., Bump J.B., Ozcelik E.A., Kutzin J., Reich M.R. (2019). Political economy analysis for health financing reform. Health Syst Reform.

[110] Ewig C. (2016). Reform and electoral competition: convergence toward equity in Latin American health sectors. Comp Polit Stud.

[111] Venkateswaran S., Slaria S., Mukherjee S. (2022). Political motivation as a key driver for universal health coverage. Front Public Health.

[112] Litewka S.G., Heitman E. (2020). Latin American healthcare systems in times of pandemic. Dev World Bioeth.

[113] Hallin D.C., Mihelj S., Ferracioli P. (2024). Pandemic communication in times of populism: politicization and the COVID communication process in Brazil, Poland, Serbia and the United States. Soc Sci Med.

[114] Baum F., Musolino C., Freeman T. (2024). Thinking politically about intersectoral action: ideas, interests and institutions shaping political dimensions of governing during COVID-19. Health Policy Plan.

[115] Savedoff W., Bernal P., Distrutti M., Goyeneche L., Bernal C. (2022).

[116] Rubinstein A., Filippini F., Santoro A. (2023). Lives versus livelihoods: the epidemiological, social, and economic impact of COVID-19 in Latin America and the Caribbean. Health Aff (Millwood).

[117] Flores W., Sullivan A., Jerez F., Rodriguez D.C. (2024). The politics of health systems policies during COVID-19: reflections on experiences from Latin America and the Caribbean. Int J Equity Health.

[118] Oliveira S.C., Machado C.V., Hein A.A., Almeida P.F. (2020). Health policies in Chile (2000-2018): trajectory and conditioning factors. Cad Saude Publica.

[119] Mayrides M., Ruiz de Castilla E.M., Szelepski S. (2020). A civil society view of rare disease public policy in six Latin American countries. Orphanet J Rare Dis.

[120] Turino F., Filippon J., Sodre F., Siqueira C.E. (2021). Reinventing privatization: a political economic analysis of the social health organizations in Brazil. Int J Health Serv.

[121] Duryea S., Pereira M.A. (2021).

[122] Rizvi S.S., Douglas R., Williams O.D., Hill P.S. (2020). The political economy of universal health coverage: a systematic narrative review. Health Policy Plan.

[123] Guizzo-Altube M., Scartascini C., Tommasi M. (2025). The political economy of redistribution and (in) efficiency in Latin America and the Caribbean. Oxford Open Econ.

[124] Bancalari A., Berlinski S., Buitrago G., García M.F., Mata D.D.L., Vera-Hernández M. (2023).

[125] Transparency International (2024). https://images.transparencycdn.org/images/CPI2024_Report_Eng1.pdf.

[126] Gedan B., Ferreira D., Alonso D., Sabet D. (2020). https://www.wilsoncenter.org/event/covid-19-and-latin-americas-epidemic-corruption.

[127] The Lancet Regional Health-Americas (2024). Corruption: possibly the biggest threat to health care. Lancet Reg Health Am.

[128] Vianna A.C., Mollick A.V. (2018). Institutions: key variable for economic development in Latin America. J Econ Bus.

[129] de Almeida S.J., Esperidião F., de Moura F.R. (2024). The impact of institutions on economic growth: evidence for advanced economies and Latin America and the Caribbean using a panel VAR approach. Int Econ.

[130] Bascolo E., Houghton N., Del Riego A. (2018). Types of health systems reforms in Latin America and results in health access and coverage. Rev Panam Salud Publica.

[131] Paschoalotto M.A.C., Lazzari E.A., Rocha R., Massuda A., Castro M.C. (2023). Health systems resilience: is it time to revisit resilience after COVID-19?. Soc Sci Med.

[132] Thu K.M., Bernays S., Abimbola S. (2024). Learning analysis of health system resilience. Health Policy Plan.

[133] Erku D., Khatri R., Endalamaw A. (2023). Community engagement initiatives in primary health care to achieve universal health coverage: a realist synthesis of scoping review. PLoS One.

[134] Haldane V., Chuah F.L.H., Srivastava A. (2019). Community participation in health services development, implementation, and evaluation: a systematic review of empowerment, health, community, and process outcomes. PLoS One.

[135] Khatri R.B., Erku D., Endalamaw A. (2023). Multisectoral actions in primary health care: a realist synthesis of scoping review. PLoS One.

[136] Bitton A., Veillard J.H., Basu L., Ratcliffe H.L., Schwarz D., Hirschhorn L.R. (2018). The 5S-5M-5C schematic: transforming primary care inputs to outcomes in low-income and middle-income countries. BMJ Glob Health.

[137] Abbas K.M., Dorratoltaj N., O’Dell M.L., Bordwine P., Kerkering T.M., Redican K.J. (2016). Clinical response, outbreak investigation, and epidemiology of the fungal meningitis epidemic in the United States: systematic review. Disaster Med Public Health Prep.

[138] Galvez-Hernandez P., Gonzalez-de Paz L., Muntaner C. (2022). Primary care-based interventions addressing social isolation and loneliness in older people: a scoping review. BMJ Open.

[139] Manfrini G., Treich R., Rumor P., Magagnin A. (2020).

[140] Fan L., Lukin W., Zhao J., Sun J., Hou X.Y. (2015). Interventions targeting the elderly population to reduce emergency department utilisation: a literature review. Emerg Med J.

[141] Goncalves-Bradley D.C., Iliffe S., Doll H.A. (2017). Early discharge hospital at home. Cochrane Database Syst Rev.

[142] Hernandez Rincon E.H., Pimentel Gonzalez J.P., Aramendiz Narvaez M.F., Araujo Tabares R.A., Roa Gonzalez J.M. (2021). Description and analysis of primary care-based COVID-19 interventions in Colombia. Medwave.

[143] Briand S., Hess S., Nguyen T., Purnat T.D., Purnat T.D., Nguyen T., Briand S. (2023). Managing infodemics in the 21st century: addressing new public health challenges in the information ecosystem.

[144] Prado N., Biscarde D., Pinto Junior E.P. (2021). Primary care-based health surveillance actions in response to the COVID-19 pandemic: contributions to the debate. Cien Saude Colet.

[145] Royal A., Mali M.A., Kumar V. (2021). Harnessing the potential of the primary healthcare facilities in India to respond COVID-19 pandemic: a scoping evidence-based research synthesis. J Family Med Prim Care.

[146] Haldane V., Zhang Z., Abbas R.F. (2020). National primary care responses to COVID-19: a rapid review of the literature. BMJ Open.

[147] Lurie T., Adibhatla S., Betz G. (2023). Mobile integrated health-community paramedicine programs’ effect on emergency department visits: an exploratory meta-analysis. Am J Emerg Med.

[148] McGowan C.R., Baxter L., Deola C. (2020). Mobile clinics in humanitarian emergencies: a systematic review. Confl Health.

[149] Rivas J. (2020). Advanced access scheduling in primary care: a synthesis of evidence. J Healthc Manag.

[150] Khanassov V, Pluye P, Descoteaux S (2016). Organizational interventions improving access to community-based primary health care for vulnerable populations: a scoping review. Int J Equity Health.

[151] da Silva B, de Vechi Corrêa A, da Silva André Uehara S (2022). Organização da atenção primária à saúde na pandemia de covid-19: revisão de escopo. Revista De Saúde Pública.

[152] Flores-Mateo G, Violan-Fors C, Carrillo-Santisteve P, Peiro S, Argimon JM (2012). Effectiveness of organizational interventions to reduce emergency department utilization: a systematic review. PLoS One.

[153] Parsons J, Salman B, Leach H, Watson E, Atherton H (2023). Training primary care staff in delivering the primary care consultation remotely: a systematic review. BJGP Open.

[154] Baral P. (2021). Health systems and services during COVID-19: lessons and evidence from previous crises: a rapid scoping review to inform the United Nations research roadmap for the COVID-19 recovery. Int J Health Serv.

[155] Lindenfeld Z, Berry C, Albert S (2023). Synchronous home-based telemedicine for primary care: a review. Med Care Res Rev.

[156] De Vera K, Challa P, Liu RH (2022). Virtual primary care implementation during COVID-19 in high-income countries: a scoping review. Telemed J E Health.

[157] Hernández Rincón E., Pimentel González J., Aramendiz Narváez M., Araujo Tabares R.A., Roa González J. (2021). Descripción y análisis de las intervenciones fundamentadas en la atención primaria para responder al COVID-19 en Colombia | Description and analysis of primary care-based COVID-19 interventions in Colombia. Medwave.

[158] Franca C, Nunes C, Aquino R (2023). Escopo de ações dos agentes comunitários de saúde na pandemia de Covid-19: Revisão da literatura [Scope of actions of community health workers in the COVID-19 pandemic: Literature review]. Trabalho, Educação e Saúde.

[159] van Ginneken N, Chin WY, Lim YC (2021). Primary-level worker interventions for the care of people living with mental disorders and distress in low- and middle-income countries. Cochrane Database Syst Rev.

[160] Robertson HD, Elliott AM, Burton C (2016). Resilience of primary healthcare professionals: a systematic review. Br J Gen Pract.

[161] Mude W., Mwenyango H., Preston R., O’Mullan C., Vaughan G., Jones G. (2024). HIV testing disruptions and service adaptations during the COVID-19 pandemic: a systematic literature review. AIDS Behav.

[162] Kamermayer A.K., Leasure A.R., Anderson L. (2017). The effectiveness of transitions-of-care interventions in reducing hospital readmissions and mortality: a systematic review. Dimens Crit Care Nurs.

[163] Wimsett J., Harper A., Jones P. (2014). Review article: components of a good quality discharge summary: a systematic review. Emerg Med Australas.

[164] Trindade L.F., Boell J.E.W., Lorenzini E. (2022). Effectiveness of care transition strategies for colorectal cancer patients: a systematic review and meta-analysis. Support Care Cancer.

[165] Weeda E., Gilbert R.E., Kolo S.J. (2023). Impact of pharmacist-driven transitions of care interventions on post-hospital outcomes among patients with coronary artery disease: a systematic review. J Pharm Pract.

[166] van den Broek S., Westert G.P., Hesselink G., Schoon Y. (2023). Effect of ED-based transitional care interventions by healthcare professionals providing transitional care in the emergency department on clinical, process and service use outcomes: a systematic review. BMJ Open.

[167] Greenwood-Lee J, Jewett L, Woodhouse L, Marshall DA (2018). A categorisation of problems and solutions to improve patient referrals from primary to specialty care. BMC Health Serv Res.

[168] Bearden T., Ratcliffe H.L., Sugarman J.R. (2019). Empanelment: a foundational component of primary health care. Gates Open Res.

[169] Pesec M., Ratcliffe H.L., Karlage A., Hirschhorn L.R., Gawande A., Bitton A. (2017). Primary health care that works: the Costa Rican experience. Health Aff (Millwood).

[170] Spigel L., Pesec M., Villegas Del Carpio O. (2020). Implementing sustainable primary healthcare reforms: strategies from Costa Rica. BMJ Glob Health.

[171] Barreto M.L., Aquino R. (2009). Recent positive developments in the Brazilian health system. Am J Public Health.

[172] Wensley C., Botti M., McKillop A., Merry A.F. (2017). A framework of comfort for practice: an integrative review identifying the multiple influences on patients’ experience of comfort in healthcare settings. Int J Qual Health Care.

[173] Rios-Zertuche D., Benitez Collante A.E., Aguilar Rivera A.M. (2023). Qualitative study of in-kind incentives to improve healthcare quality in Belize: is quality work better than wealth?. PLoS One.

[174] Rios-Zertuche D., Carter K.H., Harris K.P. (2021). Performance of passive case detection for malaria surveillance: results from nine countries in Mesoamerica and the Dominican Republic. Malar J.

[175] Zonta R., Zaros Galana M., Zepeda J. (2024). Supporting a rapid primary care response to emergent communicable disease threats with PACK (Practical Approach to Care Kit) in Florianopolis, Brazil. BMJ Glob Health.

[176] Araujo F.W.C., Rodrigues S., Carvalho T.A., de Sousa D.S., Tenorio M.D.L., Martins-Filho P.R. (2024). Misinformation, disinformation, and fake news amid the new global Mpox emergency. Rev Panam Salud Publica.

[177] Moucheraud C., Guo H., Macinko J. (2021). Trust in governments and health workers low globally, influencing attitudes toward health information, vaccines. Health Aff (Millwood).

[178] Yaddanapudi L., Hahn K., Ladikas M. (2024). A multifaceted analysis of decreasing trust in health institutions in the EU during the COVID-19 pandemic. Discover Public Health.

[179] Soranz D, Pinto LF, Penna GO (2016). Themes and Reform of Primary Health Care (RCAPS) in the city of Rio de Janeiro, Brazil. Cien Saude Colet.

[180] Aguilar G.M.O.C.L., Ribeiro C.L.P. (2025). Preparação, Vigilância e Resposta às Emergências de Saúde Pública na Cidade do Rio de Janeiro no período de 2021 a 2024. Ciência & Saúde Coletiva.

[181] Savedoff W.D., Goyeneche L., Soler L.A. (2023).

[182] Orrego Villegas S., Adjei Boakye E., Arrieta J., Posada Espana K., Vanegas M.N., Prado Pinto G. (2025). Perspectives of healthcare providers on telemedicine implementation during the COVID-19 pandemic in Colombia: a mixed-method study. Front Public Health.

[183] La telesalud como estrategia para fortalecer la atención primaria: la experiencia de Panamá. *WB/PAHO Lancet Americas Commission on Primary Health Care and Resilience in Latin America and the Caribbean*. Forthcoming.

[184] Nelson J., Savoca Truzzo F., Mendez F. (2025).

[185] da Fonseca C.B., Shibata L., Rocha M., Nelson J. AVANÇA SAÚDE SP: Driving the Digital Transformation of Primary Health Care in São Paulo. https://osf.io/preprints/osf/3f5rt_v1.

[186] R S, L S, W L, W S. Uso de la Inteligencia Artificial (IA) para fortalecer la resiliencia de la Atención Primaria en Costa Rica. WB/PAHO Lancet Americas Commission on primary health care and resilience in Latin America and the Caribbean [Forthcoming].

[187] World Bank Group (2023).

[188] Fitzpatrick K.M., Ody M., Goveas D. (2023). Understanding virtual primary healthcare with Indigenous populations: a rapid evidence review. BMC Health Serv Res.

[189] Group WB (2023). https://www.worldbank.org/en/results/2024/11/01/-transforming-health-the-world-bank-s-shift-from-crisis-response-to-building-health-system-resilience-in-latin-america-a.

[190] Chan I.L., Mowson R., Alonso J.P., Roberti J., Contreras M., Velandia-Gonzalez M. (2022). Promoting immunization equity in Latin America and the Caribbean: case studies, lessons learned, and their implication for COVID-19 vaccine equity. Vaccine.

[191] Bernal Lara P., Savedoff W.D., Garcia Agudelo M.F. (2023). Disruption of Non-COVID-19 health care in Latin America during the pandemic: effects on health, lessons for policy. Health Aff (Millwood).

[192] Doubova S.V., Leslie H.H., Kruk M.E., Perez-Cuevas R., Arsenault C. (2021). Disruption in essential health services in Mexico during COVID-19: an interrupted time series analysis of health information system data. BMJ Glob Health.

[193] Teper M.H., Vedel I., Yang X.Q., Margo-Dermer E., Hudon C. (2020). Understanding barriers to and facilitators of case management in primary care: a systematic review and thematic synthesis. Ann Fam Med.

[194] Joo J.Y., Huber D.L. (2018). Barriers in case managers’ roles: a qualitative systematic review. West J Nurs Res.

[195] Corbin J.H., Oyene U.E., Manoncourt E. (2021). A health promotion approach to emergency management: effective community engagement strategies from five cases. Health Promot Int.

[196] Mensah G.A., Johnson L.E. (2024). Community engagement alliance (CEAL): leveraging the power of communities during public health emergencies. Am J Public Health.

[197] Houghton N, Bascolo E, Zavaleta C (2025). Community-driven strategies for primary health care resilience in response to shocks in Latin America and the Caribbean: a scoping review and expert consultation. Lancet Reg Health Am.

[198] Fitzpatrick K., Sehgal A., Montesanti S. (2023). Examining the role of Indigenous primary healthcare across the globe in supporting populations during public health crises. Glob Public Health.

[199] Coombes J, Holland AJA, Ryder C (2023). Discharge interventions for First Nations people with a chronic condition or injury: a systematic review. BMC Health Serv Res.

[200] da Silva Fernandes M., Boufleuer E., Rodrigues P., de Lima Trindade L., Petri Tavares J., Pai D. (2022). Implicações da pandemia da COVID-19 sobre a Atenção Primária à Saúde: revisão integrativa. Rev Enfermagem Saúde.

[201] Das J.K., Lassi Z.S., Salam R.A., Bhutta Z.A. (2013). Effect of community based interventions on childhood diarrhea and pneumonia: uptake of treatment modalities and impact on mortality. BMC Public Health.

[202] Sacks E., Freeman P.A., Sakyi K. (2017). Comprehensive review of the evidence regarding the effectiveness of community-based primary health care in improving maternal, neonatal and child health: 3. neonatal health findings. J Glob Health.

[203] Edelman A., Marten R., Montenegro H. (2021). Modified scoping review of the enablers and barriers to implementing primary health care in the COVID-19 context. Health Policy Plan.

[204] Shafiq Y., Rubini E., Fazal Z.Z. (2024). Impact of Ebola and COVID-19 on maternal, neonatal, and child health care among populations affected by conflicts: a scoping review exploring demand and supply-side barriers and solutions. Confl Health.

[205] Bhaumik S, Moola S, Tyagi J, Nambiar D, Kakoti M (2020). Community health workers for pandemic response: a rapid evidence synthesis. BMJ Glob Health.

[206] Jha A., Lin L., Short S.M., Argentini G., Gamhewage G., Savoia E. (2018). Integrating emergency risk communication (ERC) into the public health system response: systematic review of literature to aid formulation of the 2017 WHO guideline for ERC policy and practice. PLoS One.

[207] Pan American Health Organization (2021).

[208] Cabieses B., Esnouf S., Blukacz A., Espinoza M.A., Mezones-Holguin E., Leyva R. (2022). Health in Chile’s recent constitutional process: a qualitative thematic analysis of civil proposals. Int J Environ Res Public Health.

[209] Santos J.S., Teixeira C.F. (2023). Political action analysis of the Brazilian Health care reform movement in the COVID-19 pandemic: 2020- 2021. Cien Saude Colet.

[210] Ayme S, Kole A, Groft S (2008). Empowerment of patients: lessons from the rare diseases community. Lancet.

[211] Souliotis K, Agapidaki E, Peppou LE (2018). Assessing patient organization participation in health policy: a comparative study in France and Italy. Int J Health Policy Manag.

[212] Ciapponi A, Cattivera C, Esperato Martinez A, Nieto Pereda B (2008). Database of patients’ organizations in Latin. America & the Caribbean.

[213] IPSOR (2023). Patient organizations in Latin America. https://www.ispor.org/docs/default-source/intl2024/position-paperpags-in-latam134821-pdf.pdf?sfvrsn=5c55fab2_0.

[214] Bolivar M, C R. Health systems resilience: the role of patient and user organizations in Latin America and the Caribbean. Forthcoming.

[215] Alonso V., Fuertes S., Sánchez L.P., Hoffmann M.M., Romero P.M., P P.C. (2023). Trabajar en salud durante la pandemia: experiencias de vinculación con la comunidad relativas a la producción social del cuidado en Mar del Plata, Argentina. Saude soc.

[216] Keim M.E., Runnels L.A., Lovallo A.P. (2021). Measuring the efficacy of a pilot public health intervention for engaging communities of Puerto Rico to rapidly write hurricane protection plans. Prehosp Disaster Med.

[217] Duan K.I., Rodriguez Garza F., Flores H. (2021). Economic evaluation of a novel community-based diabetes care model in rural Mexico: a cost and cost-effectiveness study. BMJ Open.

[218] Duffy S., Norton D., Kelly M. (2020). Using community health workers and a smartphone application to improve diabetes control in rural Guatemala. Glob Health Sci Pract.

[219] Hernandez S., Oliveira J.B., Mendoza Sosof C., Lawrence E., Shirazian T. (2020). Adapting antenatal care in a rural LMIC during COVID-19: a low literacy checklist to mitigate risk for community health workers. Int J Gynaecol Obstet.

[220] Parker C., Garcia F., Menocal O., Jeer D., Alto B. (2019). A mosquito workshop and community intervention: a pilot education campaign to identify risk factors associated with container mosquitoes in san Pedro Sula, Honduras. Int J Environ Res Public Health.

[221] Ramirez A.A., Pena M.K.S., Cardona J.A.D., Marin S.M.G., Londono G.C., Vargas C.E. (2022). Social participation in health: a community-based participatory research approach to capacity building in two Colombian communities. Prog Commun Health Partnersh.

[222] Tschampl C.A., Undurraga E.A., Ledogar R.J. (2020). Cost-effectiveness of community mobilization (Camino Verde) for dengue prevention in Nicaragua and Mexico: a cluster randomized controlled trial. Int J Infect Dis.

[223] Andersson N., Arostegui J., Nava-Aguilera E., Harris E., Ledogar R.J. (2017). Camino Verde (the Green Way): evidence-based community mobilisation for dengue control in Nicaragua and Mexico: feasibility study and study protocol for a randomised controlled trial. BMC Public Health.

[224] Losco L.N., SFB G. (2019). Sujeitos da saúde, agentes do território: o agente comunitário de saúde na Atenção Básica ao imigrante. Interface (Botucatu).

[225] Case study of Bolivia: Traditional Medicine and Community Empowerment for Primary Healthcare Resilience in Latin America and the Caribbean. WB/PAHO Lancet Americas Commission on Primary Health Care and Resilience in Latin America and the Caribbean*.* Forthcoming.

[226] Logroño S. (2019). Salud en movimiento: movimientos sociales y salud popular en La Plata, Argentina. Ciência Saúde Coletiva.

[227] Alonzo D., Popescu M. (2021). Utilizing social media platforms to promote mental health awareness and help seeking in underserved communities during the COVID-19 pandemic. J Educ Health Promot.

[228] World Health Organization (2018).

[229] Bernal P., Bancalari A., Muñoz M., Zúñiga Brenes P., Jara Maleš P. (2024).

[230] Bishai D., Saleh B.M., Huda M. (2024). Practical strategies to achieve resilient health systems: results from a scoping review. BMC Health Serv Res.

[231] Khatri R.B., Endalamaw A., Erku D. (2023). Preparedness, impacts, and responses of public health emergencies towards health security: qualitative synthesis of evidence. Arch Public Health.

[232] Chaudhury S., Ravicz M.M., McPherson H. (2020). Delivering primary healthcare in conflict-affected settings: a review of the literature. Am J Disaster Med.

[233] Neilson S., Chittle A., Coleman T., Kurdyak P., Zaheer J. (2020). Policies and procedures for patient transfers from community clinics to emergency departments under the mental health act: review and policy scan. Int J Law Psychiatry.

[234] Barbalho R.E., Schenkman S., Sousa A., Bousquat A. (2023). Innovative shortcuts and initiatives in primary health care for rural/remote localities: a scoping review on how to overcome the COVID-19 pandemic. Rural Remote Health.

[235] Witter S. (2012). Health financing in fragile and post-conflict states: what do we know and what are the gaps?. Soc Sci Med.

[236] Wicklum S.C., Nuique K., Kelly M.A., Nesbitt C.C., Zhang J.J., Svrcek C.P. (2023). Greening family medicine clinic operations and clinical care, where do we start? A scoping review of toolkits and aids. Fam Pract.

[237] Tello Ponce B, I D-E. Innovaciones para fortalecer la resiliencia de la Atención Primaria de Salud a futuras pandemias: lecciones aprendidas desde la respuesta de Quito a la pandemia de COVID-19. Forthcoming.

[238] Gaitán-Rossi P., Yañez-Santaolalla J., Jiménez-Ortiz A., Tapia-Hernández B.Z., Reyes-Morales H. (2025). Mexico city monitoring system during the COVID-19 pandemic: a case-study. Health Policy Technol.

[239] Palmeiro-Silva Y.K., Yglesias-Gonzalez M., Blanco-Villafuerte L. (2023). The lancet countdown South America: increasing health opportunities by identifying the gaps in health and climate change research. Lancet Reg Health Am.

[240] United Nations Development Programme, International Institute of Sustainable Development (2012). https://unfccc.int/files/adaptation/cancun_adaptation_framework/loss_and_damage/application/pdf/rajeev_isaar_undp-bcpr,_marius_keller_iisd.pdf.

[241] Lenzen M., Malik A., Li M. (2020). The environmental footprint of health care: a global assessment. Lancet Planet Health.

[242] Walsh S.J., O’Leary A., Bergin C., Lee S., Varley A., Lynch M. (2024). Primary healthcare’s carbon footprint and sustainable strategies to mitigate its contribution: a scoping review. BMC Health Serv Res.

[243] Gerk A., Forbes C., Wurdeman T. (2024). Promoting climate-resilient health systems through national surgical plans. Lancet Reg Health Am.

[244] Hartinger S.M., Palmeiro-Silva Y.K., Llerena-Cayo C. (2024). The 2023 Latin America report of the lancet countdown on health and climate change: the imperative for health-centred climate-resilient development. Lancet Reg Health Am.

[245] Pan American Health Organization (2025). https://www.paho.org/en/smarthospitals.

[246] Gutierrez C, Shapira G, Gabani J, Couffinhal A, L. DG. Resilient and effective primary health care systems for financial risk protection: a case study from Belize. Forthcoming.

[247] Lal A, Lim C, Almeida G, Fitzgerald J (2022). Minimizing COVID-19 disruption: ensuring the supply of essential health products for health emergencies and routine health services. Lancet Reg Health Am.

[248] Vammalle C, Reyes L (2022). Health budgeting and governance responses to COVID-19 in Latin America and the Caribbean: Lessons for improving health systems’ resilience. OECD J Budgeting.

[249] (2020). President of the Republic of Colombia. Decreto 417 de 2020. https://www.funcionpublica.gov.co/eva/gestornormativo/norma.php?i=110334.

[250] Congreso de La Republica Guatemala (2020). Iniciativa De Ley De Rescate Económico a Las Familias Por Los Efectos Causados Por El Covid-19 (Economic Rescue Law Initiative for Families Affected by COVID-19). https://participacion.congreso.gob.gt/wp-content/uploads/2020/04/5757.pdf.

[251] Salud M.D., Pública S.D.S. (2020). Decreto 4 Decreta Alerta Sanitaria Por El Período Que Se Señala Y Otorga Facultades Extraordinarias Que Indica Por Emergencia De Salud Pública De Importancia Internacional (ESPII) Por Brote Del Nuevo Coronavirus (2019-NCOV). https://www.bcn.cl/leychile/navegar?idNorma=1142163.

[252] Vasques JDR, Peres AM, Straub M, de Souza TL (2023). [Organization of healthcare systems to confront COVID-19: a scoping reviewOrganizacion de los sistemas de salud para hacer frente a la COVID-19: revision del alcance]. Rev Panam Salud Publica.

[253] López-Arellano O., Delgado-Campos V. (2024). La transformación del sistema público de salud en la Ciudad de México. Salud Pública de México.

[254] Bigoni A, Malik AM, Tasca R (2022). Brazil’s health system functionality amidst of the COVID-19 pandemic: an analysis of resilience. Lancet Reg Health Am.

[255] OECD. Fiscal Sustainability of Health Systems (2015). Bridging Health and Finance Perspectives.

[256] De Foo C, Verma M, Tan SY (2023). Health financing policies during the COVID-19 pandemic and implications for universal health care: a case study of 15 countries. Lancet Glob Health.

[257] World Health Organization (2019). Primary health care on the road to universal health coverage: 2019 global monitoring report.

[258] Wolfrom L. (2022). Could insurance provide an alternative to fiscal support in crisis response?.

[259] Glass LT, Schlachta CM, Hawel JD, Elnahas AI, Alkhamesi NA (2022). Cross-border healthcare: A review and applicability to North America during COVID-19. Health Policy Open.

[260] OECD (2022). Dispelling Myths about Participatory Budgeting across Levels of Government.

[261] Wanwong Y, Sirinard N, Nopparattayaporn P, Poungkanta W, Putthasri W, Suphanchaimat R (2017). Health insurance for undocumented migrants: a literature review in developed countries. JMAT.

[262] Bilazarian A., Hovsepian V., Kueakomoldej S., Poghosyan L. (2021). A systematic review of primary care and payment models on emergency department use in patients classified as high need, high cost. J Emerg Nurs.

[263] Diaconu K, Falconer J, Verbel A, Fretheim A, Witter S (2021). Paying for performance to improve the delivery of health interventions in low- and middle-income countries. Cochrane Database Syst Rev.

[264] Morgan SR, Chang AM, Alqatari M, Pines JM (2013). Non-emergency department interventions to reduce ED utilization: a systematic review. Acad Emerg Med.

[265] Lindner L, Lorenzoni L (2023). Innovative providers’ payment models for promoting value-based health systems: Start small, prove value. and scale up.

[266] Cruz Santiago C., Luna Ruiz G., Ramirez Gamez J. (2015).

[267] Ahumada A., Herrera C.A., E T., Ratcliffe H.L. (2022). https://www.improvingphc.org/measuring-primary-health-care-system-performance-using-shared-monitoring-system-chile.

[268] Blas M.M., Reinders S., Alva A. (2023). Effect of the Mamas del Rio programme on essential newborn care: a three-year before-and-after outcome evaluation of a community-based, maternal and neonatal health intervention in the Peruvian Amazon. Lancet Reg Health Am.

[269] Sriram V., Brophy S.A., Sharma K., Elias M.A., Mishra A. (2023). Associations, unions and everything in between: contextualising the role of representative health worker organisations in policy. BMJ Glob Health.

[270] World Health Organization (2024).

[271] Americas Business Dialogue (2023).

[272] Curioso W.H. (2019). Building capacity and training for digital health: challenges and opportunities in Latin America. J Med Internet Res.

[273] Conduah A.K., Ofoe S., Siaw-Marfo D. (2025). Data privacy in healthcare: global challenges and solutions. Digit Health.

[274] Rothstein M.A. (2020). Public health and privacy in the pandemic. Am J Public Health.

