# Supplementary material for: No time to wait: resilience as a cornerstone for primary health care across Latin America and the Caribbean, a World Bank-PAHO Lancet Regional Health Americas Commission
Source: Lancet Reg Health Am. 2025 Sep 29;50:101240. doi: 10.1016/j.lana.2025.101240 (PMC12541825; doi:10.1016/j.lana.2025.101240)
Supplement: Supplementary Materials [file mmc1.docx]

No Time to Wait: Resilience as a Cornerstone for Primary Health Care Across Latin America and the Caribbean

The World Bank-PAHO Lancet regional health Americas Commission

Supplementary Materials

Table of Contents

[Supplementary Material 1. Domains, ideal scenarios, objectives, and policy options 2](#_Toc207541649)

[Supplementary Material 2. Commission methodology 15](#_Toc207541650)

[Supplementary Material 3. Research gaps organized by PHC resilience domain and sub-domain 24](#_Toc207541651)

[Supplementary Material 4. Prioritized policy options in Chile and the Dominican Republic 30](#_Toc207541652)

## **Supplementary Material 1. Domains, ideal scenarios, objectives, and policy options**

Table 1. Integrated service delivery and essential public health functions: Full description of domains, ideal scenarios, objectives and policy options.

| **Integrated Service Delivery and Essential Public Health Functions** |
| --- |
| 1. **Models of PHC that provide services for all** |
| **Ideal scenario:**  A clearly established comprehensive, equitable and evidence-informed model of primary care in which the population living in each primary care facility’s catchment area is registered, and the service provision can be universal, comprehensive and oriented to meet people’s needs in a culturally competent way addressing inequalities. |
| **Objectives:**   - Empanelling the population enabling the rapid identification of people who may be vulnerable to different types of shocks and emergencies. - Providing person-centered, comprehensive primary care services, including community outreach, so that people who need it more can receive quality care in an adequate and convenient way in responding to crises. |
| **Policy options**   - Registries/enrollment/mapping to identify populations within catchment areas, especially vulnerable/priority populations (e.g., migrants, elderly including those in nursing homes, people with disabilities) and screening of population (e.g. by gender, age, existing conditions etc.) for potential and existent vulnerabilities.^1,2^ - Interprofessional teams (whose composition may vary by local conditions, but may be composed of medical doctors, nurses, community health workers, and social workers) with complementary skillsets who can provide collaborative person-centered care.^3^ - Outreach services into the community – including home-based care (e.g., for elderly, people with disabilities), schools and other community facilities/associations, seeking their participation in crisis preparedness and response activities for the less advantaged.^1,4,5^ - Robust quality assurance systems, monitoring tools and improvement mechanisms to provide person-centered and quality PHC services. - Mechanisms to understand user experience to improve person-centeredness during care.^6^ |
| 1. **Integrating essential public health functions in PHC** |
| **Ideal scenario:**  Agencies responsible for providing essential public health functions such as surveillance, disease prevention and health promotion work seamlessly with primary care, and many of these essential public health functions (including effective surveillance and health promotion) are embedded within PHC-based systems to effectively address the social determinants of health. |
| **Objectives**   - Embedding health surveillance at the primary care level by empowering primary care workers to detect and respond to vulnerabilities within their catchment community. - Having effective local-level collaboration across different government sectors to develop emergency preparedness plans and detect and respond to outbreaks and other risks. - Providing effective and clear communication about risks and prevention measures through multiple means of dissemination including media to tackle misinformation and fake news. |
| **Policy options**   - Primary care workers training and technical support in data analysis and adapting information systems. - Specialist epidemiological teams (e.g., from the Health Department, or National Public Health Institutes) provide support to primary care facilities for health surveillance.^7^ - Regular health situation assessments conducted by primary care to detect risk factors and vulnerabilities and work with communities to address priority health problems and strengthen participation (e.g., community resources maps).^2,8^ - Having specific programs to promote health, including health education and opportunities to promote healthy practices, local environment and work conditions to address the social determinant of heath and the most prevalent conditions and vulnerabilities. - Using primary care units as sentinel sites for the early identification of outbreaks and other risks.^8,9^ - Implement strategies to provide PHC staff with multiple different communication modalities that reflect local people’s preferences to inform the communities about health risks and preventive measures and to face misinformation and fake news. |
| 1. **Maintaining essential health care services in PHC** |
| **Ideal scenario:**  Primary care facilities are seen as an integral part of crisis response and are equipped with necessary resources and skills. PHC-based systems prioritize the provision of equitable care and the protection of vulnerable populations, ensuring the continuity of essential health services, as well as an effective response to the crisis. |
| **Objectives:**   - Reinforcing and sustaining primary care services during emergencies, so they can maintain access to essential care (including the care of shocks’ consequences) during crises, and reopen as soon as possible if they are forced to close, analyzing their impacts with an equity, clarity and sustainability criterion. |
| **Policy options**   - Well established and tested emergency response plans to keep primary care facilities open and maintaining access to essential services during an emergency so as to ensure that the population have access to key services (e.g., maternal health care, vaccination services, ongoing services for NCDs & mental health).^10^ - Effective, safe and equitable access to primary care services using: Digital and remote services (including remote monitoring)/telemedicine & teleassistance & telehealth; Adapted schedules and strategies for community communication and participation; Out-of-hours services; Providing services in open air or other solutions to prevent infections; Increased options for providing care at home and in the community (including CHWs, mobile clinics, outreach); Ensuring that primary care facilities have scheduling systems to reduce waiting times (where possible) and prevent inequalities or keeping the waiting environments safe.^7,11-13^ - When responding to a crisis caused by a pathogen, ensuring that PHC-based systems are capable of responding to mild and moderate cases using: Training programs for the health workforce; Modifying patient flows through facilities to separate infectious and non-infectious populations; Communicating to the population clarifying when they should seek primary care and when it is appropriate to go to higher-level facilities; Have referral policies to upper-care levels for high priority cases.^14-18^ - Ensuring that the right infrastructure and equipment is available in primary care to provide essential health services, including: Internet connection at all primary care facilities; Safety materials for health professionals during a crisis (e.g., personal protective equipment); Vaccines and medicines (ensuring an effective cold chain).; Information backup services; Guidelines and protocols to efficiently provide health care services.^19,20^ - Ensuring that primary care workforce is adequate to provide essential health services, including: An adequate health professionals planning, that includes plans during a shock and putting plans in place in advance, for hiring them to ensure proper staffing during crisis; Training health workers to respond to the consequences of crises, such as mental health & Gender-Based Violence; Providing extra allowances or incentives (e.g., compensatory time off); Mental health care services for health professionals to cope with occupational stress and burnout; primary care workers are kept up-to-date on the latest treatment protocols through established systems for communications.^18,21-24^ - Ensuring that vaccines and medicines are available in primary care to provide essential health services, including: Effective distribution of medicines to patients, especially those with chronic conditions (e.g., in their homes, or within convenient pick-up spots in the community); Effective supply chain and logistics of medicines and vaccines to maintain essential health services.^22,25^ |
| 1. **Ensuring that the specialized health care network engages appropriately with PHC** |
| **Ideal situation:** Primary care services are the cornerstone of the health system and are effectively integrated with the specialized care networks. Referral networks, strong information systems and communication channels exist between primary care facilities and specialized health services, in order to ensure continuity of care and the most effective and efficient use of resources. |
| **Objectives:**   - Building or strengthening regulation and referral systems to manage the flow of patients between primary care facilities and specialized & hospital care, and after system discharge, so as to ensure continuity and coordination of care. - Strengthening the clinical capacity and availability of services at the primary care level to avoid unnecessary referrals. |
| **Policy options:**   - Systems to provide appropriate support after hospital discharge including: Multiple -ways communication channels (e.g., reference and counter reference) between different-level facilities (e.g., hospitals, specialty services, etc.); Dedicated staff to manage discharge planning; Clinical protocols for post—discharge care.^26-30^ - Systems to provide appropriate support referral systems (especially to rehabilitation services which are critical during shocks) to higher-level facilities including: Patient transport systems between primary care and higher-level facilities; Information flows and direct communication between levels (counter-referral); Digital technology to support referral systems; Waiting-list management; Aligning medicine supplies and diagnostic capacities with referral rules and conditions to be treated at the primary care level; Effective communication channels between higher level facilities and primary care to promote dissemination of up-to-date  treatment guidelines etc^13,31^ - Supplying primary care facilities with inputs, equipment, technologies, medicines to provide effective and quality clinical services in primary care avoiding unnecessary referrals. - Well-trained health professionals that provide effective and quality clinical services in primary care avoiding unnecessary referrals. |

Table 2. Community participation/empowerment: Full description of domains, ideal scenarios, objectives and policy options.

| **Community participation/empowerment** |
| --- |
| **1. Community engagement** |
| **Ideal scenario:**  Institutionalized opportunities for meaningful community engagement in primary care that include dedicated spaces for decision making, knowledge exchange and innovation between communities and health care providers, including community health workers, which facilitates the co-ownership and co-responsibility in facing external shocks, as well as transparency and accountability in decision-making processes. |
| **Objectives:**   - Building and strengthening legal frameworks to guarantee social participation in primary care, ensuring their sustainable funding, transparency and accountability measures. - Incorporating social and community organizations to co-design and implement local health actions, including decision-making in issues such as service delivery and budget allocation, and plans to prepare, respond and recover from external shocks. |
| **Policy options**   - Legal and regulatory frameworks that institutionalise opportunities for community participation (e.g., recurring community input into local plans to prepare and respond to public health emergencies). - Permanent, regular and institutionalized spaces of dialogue, knowledge exchange (*Dialogo de Saberes*) and decision-making between communities and primary care providers/authorities at national and sub-national level.^32-34^ - Inclusive participatory oversight that ensures application of legal and regulatory frameworks as part of transparency and accountability mechanisms at all levels of decision-making. - Regular meetings between communities and primary care providers/authorities (at least quarterly) to plan and implement programs. - Ongoing emergency preparedness and response, that includes continuous and exhaustive mapping of actors, of knowledge systems and of capacities.^2^ - Development of community-based innovations to build resilience.^35-37^ - Dissemination of community engagement resources (including community-based innovations) to build resilience across communities, including capacity building and technical cooperation.^18,33,37^ - Training primary care managers and workers in community engagement and risk assessment priorities.^38^ - Transparent, participatory and institutionalized involvement of local community health workers (CHWs) and ancestral providers in decision making.^5,39^ - Using CHWs in supporting the delivery of primary care services under normal conditions and during emergencies (including task shifting and the uses of technologies), ensuring that they are formal paid staff.^7,40,41^ - Support community members, leaders, and community-based organizations to provide health promotion, prevention, and monitoring activities during emergencies (e.g., health risk communication, distribution of essentials and other social needs). - Leverage networks of community leaders and community-based organizations that collaborate with each other and other sectors to enhance the reach of interventions (e.g., health workers, churches, academia, social services, civil society organizations). - Partner with CSOs to support the response to public health emergencies (e.g., health risk communication, distribution of essentials and other social needs). |
| **2. Interculturality** |
| **Ideal scenario:**  Culturally representative primary health services integrating diverse cultural and ancestral health practices in building resilience. |
| **Objectives**   - Promoting an intercultural approach in the organization of primary care recognizing the knowledge, practices and providers of diverse peoples and communities. - Establishing an intercultural delivery of primary care services with meaningful participation of communities and adequate/diverse financial/human resources. |
| **Policy options**   - Develop health policies and programs with an intercultural approach, that promote traditional and complementary medicine and educate the public on intercultural health issues. - Establishing and financing intercultural delivery of primary care services with full and effective participation of communities. - Continuous education and health care workers training programs that formally integrate interculturality. - Recruiting and retaining health personnel that reflects the ethnic and cultural diversity of local communities.^160^ - Create intercultural spaces for health care delivery that are integrated with health center activities. |
| **3. Communication and trust** |
| **Ideal scenario:**  Consistent, transparent and respectful communication between primary care providers and community members, fostering deep trust and engagement, in order to engage community assets and capacities to respond to external shocks and tackling miscommunication and fake news. |
| **Objectives:**   - Using respectful and dignified interactions and communications between primary care providers and communities, ensuring that health messages (including health risks and crisis communications) are culturally relevant and linguistically accessible. - Facilitating communications from health authorities to the community by empowering communities to improve their health literacy and using trusted community leaders to sensitize populations to a variety of health risks. |
| **Policy options**   - Co-creation of the design and delivery of health and risk messages with trusted community representatives to deliver health messages to reach diverse populations. - Creating communication materials that will be culturally appropriate and available in local languages to deliver health and risk messages.^219^ - Using multiple channels to communicate health and risk messages to reach diverse populations.^214^ |

Table 3. Multi-sectoral action: Full description of domains, ideal scenarios, objectives and policy options.

| **Multi-sectoral action** |
| --- |
| **Intersectoral engagement** |
| **Ideal scenario:**  The intersectoral collaboration is embedded at both national and local levels, including a whole-of-society and social determinants of health approach, to support the effective delivery of essential primary care services and non-health interventions along the resilience cycle. |
| **Objectives**   - Complementing social protection systems to ensure basic human needs are met with health and social services at the local level during and after the occurrence of shock. - Coordinating non-health sectors (e.g., education, public security, etc.), including a whole-of-society and social determinants of health approach before and during the emergencies, to facilitate the provision of primary care essential services and the delivery of non-health care actions. |
| **Policy options**   - Intersectoral entity or group at the national and local level that integrates health and non-health sectors to prepare and respond to crises.^42,43,45,47^ - National and local plans to prepare for emergencies that explicitly include a primary care and social determinants of health perspective and addresses different types of risks (multi-hazard) to service provision (e.g., hurricanes to damage infrastructure, ensuring primary care worker’s security during mass casualty events).^32,45^ - National and local plans to prepare for emergencies that identifies actions at primary care level and network to provide non-health services (e.g. financial support, food aids, subsidies, guaranteed minimum income, etc.).^38,42,44^ - Mapping and utilizing relevant public and private non-health actors and capacity that could contribute to the PHC service provision during shocks, including infrastructure, transport, among others (e.g., hotels, sport facilities, shelters, schools, warehouses, etc.).^42,45^ - Non-health sector support for health workers to continue providing primary care services (e.g. childcare, financial relief, occupational health). - Leveraging the role of non-health workers (e.g. police, army) and community members.^10,48^ - Basic services (drinkable water, energy, security) assuring mechanisms (e.g. back up).^43^ - Harmonise the local primary care response to comply with the International Health Regulations and future related international agreements. |
| **Private for and not-for-profit health sector engagement** |
| **Ideal scenario:**  Institutionalized public-private collaborations across the resilience cycle to ensure primary care service provision following public health goals. |
| **Objectives:**   - Effectively engaging and strengthening collaborations with the private (for and non-for-profit) health sector to sustain primary care essential services during emergencies. |
| **Policy options**   - Regulatory instruments (legislation, plans, bylaws, etc.) for public-private collaboration with roles, contracting-out rules, funding and oversight to articulate service provision.^38,39,42-45^ - Convening private and public actors to plan, respond and learn to sustain primary care essential services during shocks, including contracting-out and other types of private-public partnerships.^42,44^ - Capacity assessment (e.g. infrastructure, equipment) of the private sector and its complementarity with the public sector to increase surge capacity.^10,39,46^ - Accountability mechanisms through periodic public reports that would allow a quick understanding of surge capacities and financial transparency in the public-private collaboration.^38,47^ - Having an independent public entity that oversees and enforces the rules for the public and private sector’s provision of primary care services. |
| **Governance, production, and use of data** |
| **Ideal scenario:**  Efficient and permanent intersectoral communication systems that include data collection and analysis at all levels, as well as knowledge management for local decision making in the context of shocks. |
| **Objectives:**   - Strengthening the governance, production and uses of data to facilitate whole-of-government system resilience data sharing across sectors. |
| **Policy options**   - Standardizing data (e.g. nominal ID, including electronic health record) to facilitate sharing between sectors (linked to empanelment).^38,43^ - Regulating data sharing transparency and privacy between sectors. - Data collection from non-administrative sources (e.g. surveys, civil society, social media).^42^ - Building intersectoral dashboards with key indicators across the resilience cycle at the national and local levels.^42,47^ - Creating risk and vulnerability maps using multisectoral data to inform action plans at the local level (geographical, disadvantaged groups).^43,47^ - Building capacity of teams at the national and local level to collect, analyze, and communicate data insights with an intersectoral approach. |
| **Environmental considerations** |
| **Ideal scenario:**  PHC-based systems comprehensively plan for health adaptation and mitigation to climate change, taking advantage of enabling conditions, considering key system vulnerabilities and promoting climate-resilient actions within the community. |
| **Objectives:**   - Adapting and planning actions to build a climate and environmental resilient PHC. |
| **Policy options**   - Supporting the generation of national and sub-national plans that integrate a multi-hazard approach by developing territorial risk assessment impact studies that include the impact on primary care network and provisions.^7,42^ - Providing climate information for health to citizens through the primary care facilities.^49^ - Implement PHC-led community programs that promote locally relevant climate-friendly actions, such as waste reduction and management, air quality improvement, clean energy adoption and sustainable transport.^49^ - Develop and implement training programs for healthcare providers to integrate climate change and environmental health considerations into all aspects of primary care service delivery (and retaining them once trained).^49^ - Building and refurbishing primary care facilities with a green perspective (e.g. site and context, day lighting, natural ventilation, solar shading, insulation, roof and wall reflectivity, exterior planting, solar panels, etc.). - Securing sustainable funding for climate-related health initiatives. |

Table 4. Financing: Full description of domains, ideal scenarios, objectives and policy options.

| **Financing** |
| --- |
| 1. **Financial governance** |
| **Ideal scenario:**  Rapid, transparent, sustainable and effective resource (re)allocation between national and local governments that facilitate a resilient and responsive PHC-based systems for health emergencies. |
| **Objectives:**   - Implementing flexible financing mechanisms enabling swift mobilization and relocation of funds dedicated to primary care allowing to facilitate a rapid and effective response to health emergencies. - Building sustainable emergency funds, at regional, national, and global levels to provide reliable financial sources, reducing dependency on ad-hoc international aid, and ensuring immediate access to primary care resources locally. |
| **Policy options**   - Emergency financial decrees drafted ahead of time with clear protocols that enable targeted funding for urgent PHC activities (e.g., community health outreach, vaccinations, and essential services).^44-47^ - Centralized fund management with empowered local execution to ensure that primary care resources reach communities effectively and equitably.^48,49^ - Regular financial risk assessments to determine what potential shocks and what populations will be most affected.^50^ - Dedicated funds for health emergencies to enable local governments (based on their risk assessment) to quickly access and utilise resources when needed.^10,48^ - Flexible legal mechanisms allowing for swift mobilization and reallocation of funds dedicated to primary care needs during emergencies.^44^ - Tracking and monitoring systems for funds assigned for PHC resilience to increase accountability.^51^ - Financial coordination mechanisms to have structured dialogues between financial officers of various sectoral ministries and the Ministry of Finance.^2,48,52^ |
| 1. **Revenue raising and pooling resources** |
| **Ideal scenario:**  Robust, coordinated financial mechanisms to ensure sufficient, readily accessible financing for prevention, preparedness, response and recovery to minimise volatility during external shocks. |
| **Objectives**   - Strengthening legal frameworks to ensure adequate funds in resilience as well as diversified funding sources to provide universal primary care services during public health emergencies. - Encouraging financial investments and budget allocations to PHC resilience in preparing for external shocks. |
| **Policy options**   - Regulating financial protections (e.g., taxes) to safeguard funds during emergencies.^53^ - Diversify funding sources through different types of taxes (health-related, sin, and production taxes) to fund resilience activities.^53,54^ - Reducing the dependency of funding from vulnerable or directly affected populations, such as user fees, which makes also PHC service freely available during crises.^44,50^ - Emergency fund to invest in resilience, encouraging contributions from different local governments, potentially through multilateral or national/subnational arrangements (the OECD recommends investing 1.4% of GDP in resilience).^55^ - Insurance mechanisms or re-insurance mechanisms across countries to provide a collective buffer against public health emergencies and support recovery strategies after a shock.^56,57^ - Mechanisms to accept funds from humanitarian and development organizations efficiently. - Implementing special taxes or levies to establish a dedicated funding stream for health initiatives aimed at resilience to reduce the reliance on emergency resource mobilization when a crisis occurs.^53^ - Enabling efficient transfers from national-level governments to support local governments in managing emergencies.^10^ - Specific budgetary provisions for resilience and emergency preparedness within health and other relevant sectors.^44^ - Determining essential health services, their costs, and potential additional costs due to shocks, including infrastructure, medicines, personnel, and transportation needs (e.g., cash transfers to maintain and rebuild). |
| 1. **Management and allocation of funds** |
| **Ideal scenario:**  Mechanisms that guarantee primary care has timely and reliable access to resources, mobilizing them swiftly during and after a shock to ensure readiness and responsiveness in emergencies. |
| **Objectives:**   - Building a strong public financial management system and organization that plan and allocate targeted resources by population needs with flexibility and using an approach that involves the community. |
| **Policy options**   - Benefit package for health crisis management that incorporates planning and forecasting to enable flexible resource allocation, adaptable to evolving needs during emergencies.^53,58^ - Defining rules and procedures that allow for rebudgeting and modification of the benefit package as circumstances change.^44^ - Forecast analyses to identify vulnerable populations and funding recipients based on shock needs for effective resource deployment. - Accountability systems supported by data to increase the transparency of fund transfers related to shock costs.^44,54^ - Guidelines for re-budgeting within and across sectors and administrative levels to reduce investment duplication and improve efficiency in the allocation of resources (e.g., military involvement in delivery, how to account for financial contribution so there is a use of duplicate funds/budget for something else).^44^ - Financing innovative community-driven proposals with flexible funding to effectively engage the community.^59^ - Ensuring essential health services are available and accessible for all populations across various jurisdictions, including disaster-affected migrants.^60^ |
| 1. **Purchasing and payment mechanisms** |
| **Ideal scenario:**  A system capable of providing prompt, adaptable financial support to primary care providers during crises, ensuring continuous delivery of essential and emergency response services. |
| **Objectives:**   - Strengthening streamlined, flexible, transparent, and autonomous purchasing and payment mechanisms to guarantee the efficient procurement of goods and services to boost resilience. |
| **Policy options**   - Hazard pay and overtime compensation mechanisms for health care workers during emergencies.^10,61^ - Payroll adjustments to allow the hiring of temporary health care workers or mobile personnel, as well as task shifting to meet demand during emergencies.^61-63^ - Centralized, collective purchasing agreements to guarantee the procurement of goods and services at favorable rates or prices.^10,44^ - Electronic payment systems to facilitate timely compensation of health care workers and suppliers and support international transactions when applicable.^58,64^ - Legal protections and anti-fraud measures to ensure a certain degree of autonomy by purchasers during and after crises.^54^ - Ensuring cost-effectiveness in procuring products and services for timely response. - Risk assessments to strengthen supply-chain management during emergencies. - Signing ex-ante contracts with suppliers for emergency needs (e.g., those required during hurricanes). - Centralized procurement mechanisms (e.g., open contracting in Guatemala, National Health Fund in Jamaica, CENABAST in Chile) to achieve better pricing, including through revolving funds for pooled purchasing of emergency supplies. - Diversify suppliers to reduce dependency on single sources and increase resilience in supply chains. - Allowing the purchase of services in other jurisdictions, enabling service portability and reimbursement rules across systems. - Financial disincentives to ensure providers do not unjustifiably increase their prices during and after shocks. |

**References:**

1. Galvez-Hernandez P, Gonzalez-de Paz L, Muntaner C. Primary care-based interventions addressing social isolation and loneliness in older people: a scoping review. *BMJ Open* 2022; **12**(2): e057729.

2. Manfrini G, Treich R, Rumor P, Magagnin A. Primary health care actions in natural disasters. Florianópolis, Santa Catarina, Brasil. , 2020.

3. Fan L, Lukin W, Zhao J, Sun J, Hou XY. Interventions targeting the elderly population to reduce emergency department utilisation: a literature review. *Emerg Med J* 2015; **32**(9): 738-43.

4. Goncalves-Bradley DC, Iliffe S, Doll HA, et al. Early discharge hospital at home. *Cochrane Database Syst Rev* 2017; **6**(6): CD000356.

5. Hernandez Rincon EH, Pimentel Gonzalez JP, Aramendiz Narvaez MF, Araujo Tabares RA, Roa Gonzalez JM. Description and analysis of primary care-based COVID-19 interventions in Colombia. *Medwave* 2021; **21**(3): e8147.

6. Wensley C, Botti M, McKillop A, Merry AF. A framework of comfort for practice: An integrative review identifying the multiple influences on patients' experience of comfort in healthcare settings. *Int J Qual Health Care* 2017; **29**(2): 151-62.

7. Abbas KM, Dorratoltaj N, O'Dell ML, Bordwine P, Kerkering TM, Redican KJ. Clinical Response, Outbreak Investigation, and Epidemiology of the Fungal Meningitis Epidemic in the United States: Systematic Review. *Disaster Med Public Health Prep* 2016; **10**(1): 145-51.

8. Prado N, Biscarde D, Pinto Junior EP, et al. Primary care-based health surveillance actions in response to the COVID-19 pandemic: contributions to the debate. *Cien Saude Colet* 2021; **26**(7): 2843-57.

9. Royal A, Mali MA, Kumar V, et al. Harnessing the potential of the primary healthcare facilities in India to respond COVID-19 pandemic: A scoping evidence-based research synthesis. *J Family Med Prim Care* 2021; **10**(1): 116-21.

10. Haldane V, Zhang Z, Abbas RF, et al. National primary care responses to COVID-19: a rapid review of the literature. *BMJ Open* 2020; **10**(12): e041622.

11. Lurie T, Adibhatla S, Betz G, et al. Mobile integrated health-community paramedicine programs' effect on emergency department visits: An exploratory meta-analysis. *Am J Emerg Med* 2023; **66**: 1-10.

12. McGowan CR, Baxter L, Deola C, et al. Mobile clinics in humanitarian emergencies: a systematic review. *Confl Health* 2020; **14**: 4.

13. Rivas J. Advanced Access Scheduling in Primary Care: A Synthesis of Evidence. *J Healthc Manag* 2020; **65**(3): 171-84.

14. Khanassov V, Pluye P, Descoteaux S, et al. Organizational interventions improving access to community-based primary health care for vulnerable populations: a scoping review. *Int J Equity Health* 2016; **15**(1): 168.

15. da Silva B, de Vechi Corrêa A, da Silva André Uehara S. Organização da atenção primária à saúde na pandemia de covid-19: revisão de escopo. *Revista De Saúde Pública* 2022; **56**: 94.

16. Flores-Mateo G, Violan-Fors C, Carrillo-Santisteve P, Peiro S, Argimon JM. Effectiveness of organizational interventions to reduce emergency department utilization: a systematic review. *PLoS One* 2012; **7**(5): e35903.

17. Parsons J, Salman B, Leach H, Watson E, Atherton H. Training primary care staff in delivering the primary care consultation remotely: a systematic review. *BJGP Open* 2023; **7**(4).

18. Baral P. Health Systems and Services During COVID-19: Lessons and Evidence From Previous Crises: A Rapid Scoping Review to Inform the United Nations Research Roadmap for the COVID-19 Recovery. *Int J Health Serv* 2021; **51**(4): 474-93.

19. Lindenfeld Z, Berry C, Albert S, et al. Synchronous Home-Based Telemedicine for Primary Care: A Review. *Med Care Res Rev* 2023; **80**(1): 3-15.

20. De Vera K, Challa P, Liu RH, et al. Virtual Primary Care Implementation During COVID-19 in High-Income Countries: A Scoping Review. *Telemed J E Health* 2022; **28**(7): 920-31.

21. van Ginneken N, Chin WY, Lim YC, et al. Primary-level worker interventions for the care of people living with mental disorders and distress in low- and middle-income countries. *Cochrane Database Syst Rev* 2021; **8**(8): CD009149.

22. Hernández Rincón E, Pimentel González J, Aramendiz Narváez M, Araujo Tabares RA, Roa González J. Descripción y análisis de las intervenciones fundamentadas en la atención primaria para responder al COVID-19 en Colombia | Description and analysis of primary care-based COVID-19 interventions in Colombia. *Medwave* 2021.

23. Robertson HD, Elliott AM, Burton C, et al. Resilience of primary healthcare professionals: a systematic review. *Br J Gen Pract* 2016; **66**(647): e423-33.

24. de Jesus França C, Abdon Nunes C, Aquino R, de Brito Lima Prado N. Escopo de ações dos agentes comunitários de saúde na pandemia de Covid-19: revisão da literatura. *Trabalho, Educação e Saúde* 2023; **21**.

25. Mude W, Mwenyango H, Preston R, O'Mullan C, Vaughan G, Jones G. HIV Testing Disruptions and Service Adaptations During the COVID-19 Pandemic: A Systematic Literature Review. *AIDS Behav* 2024; **28**(1): 186-200.

26. Kamermayer AK, Leasure AR, Anderson L. The Effectiveness of Transitions-of-Care Interventions in Reducing Hospital Readmissions and Mortality: A Systematic Review. *Dimens Crit Care Nurs* 2017; **36**(6): 311-6.

27. Wimsett J, Harper A, Jones P. Review article: Components of a good quality discharge summary: a systematic review. *Emerg Med Australas* 2014; **26**(5): 430-8.

28. Trindade LF, Boell JEW, Lorenzini E, et al. Effectiveness of care transition strategies for colorectal cancer patients: a systematic review and meta-analysis. *Support Care Cancer* 2022; **30**(7): 6251-61.

29. Weeda E, Gilbert RE, Kolo SJ, et al. Impact of Pharmacist-Driven Transitions of Care Interventions on Post-hospital Outcomes Among Patients With Coronary Artery Disease: A Systematic Review. *J Pharm Pract* 2023; **36**(3): 668-78.

30. van den Broek S, Westert GP, Hesselink G, Schoon Y. Effect of ED-based transitional care interventions by healthcare professionals providing transitional care in the emergency department on clinical, process and service use outcomes: a systematic review. *BMJ Open* 2023; **13**(3): e066030.

31. Greenwood-Lee J, Jewett L, Woodhouse L, Marshall DA. A categorisation of problems and solutions to improve patient referrals from primary to specialty care. *BMC Health Serv Res* 2018; **18**(1): 986.

32. Fitzpatrick K, Sehgal A, Montesanti S, et al. Examining the role of Indigenous primary healthcare across the globe in supporting populations during public health crises. *Glob Public Health* 2023; **18**(1): 2049845.

33. Coombes J, Holland AJA, Ryder C, et al. Discharge interventions for First Nations people with a chronic condition or injury: a systematic review. *BMC Health Serv Res* 2023; **23**(1): 604.

34. Fitzpatrick KM, Ody M, Goveas D, et al. Understanding virtual primary healthcare with Indigenous populations: a rapid evidence review. *BMC Health Serv Res* 2023; **23**(1): 303.

35. da Silva Fernandes M, Boufleuer E, Rodrigues P, de Lima Trindade L, Petri Tavares J, Pai D. Implicações da pandemia da COVID-19 sobre a Atenção Primária à Saúde: revisão integrativa. *Revista de Enfermagem e Saúde* 2022; **24**(70085).

36. Das JK, Lassi ZS, Salam RA, Bhutta ZA. Effect of community based interventions on childhood diarrhea and pneumonia: uptake of treatment modalities and impact on mortality. *BMC Public Health* 2013; **13 Suppl 3**(Suppl 3): S29.

37. Sacks E, Freeman PA, Sakyi K, et al. Comprehensive review of the evidence regarding the effectiveness of community-based primary health care in improving maternal, neonatal and child health: 3. neonatal health findings. *J Glob Health* 2017; **7**(1): 010903.

38. Edelman A, Marten R, Montenegro H, et al. Modified scoping review of the enablers and barriers to implementing primary health care in the COVID-19 context. *Health Policy Plan* 2021; **36**(7): 1163-86.

39. Shafiq Y, Rubini E, Fazal ZZ, et al. Impact of Ebola and COVID-19 on maternal, neonatal, and child health care among populations affected by conflicts: a scoping review exploring demand and supply-side barriers and solutions. *Confl Health* 2024; **18**(1): 12.

40. Sripad P, Casseus A, Kennedy S, et al. "Eternally restarting" or "a branch line of continuity"? Exploring consequences of external shocks on community health systems in Haiti. *J Glob Health* 2021; **11**: 07004.

41. Franca C, Nunes C, Aquino R, NMBL P. Escopo de ações dos agentes comunitários de saúde na pandemia de Covid-19: Revisão da literatura [Scope of actions of community health workers in the COVID-19 pandemic: Literature review]. . *Trabalho, Educação e Saúde* 2023; **21**.

42. Logroño S. Salud en movimiento: movimientos sociales y salud popular en La Plata, Argentina. . *Ciência & Saúde Coletiva* 2019; **24**(12).

43. Jha A, Lin L, Short SM, Argentini G, Gamhewage G, Savoia E. Integrating emergency risk communication (ERC) into the public health system response: Systematic review of literature to aid formulation of the 2017 WHO Guideline for ERC policy and practice. *PLoS One* 2018; **13**(10): e0205555.

44. Vammalle C, Reyes L. Health budgeting and governance responses to COVID-19 in Latin America and the Caribbean: Lessons for improving health systems’ resilience. . *OECD Journal on Budgeting* 2022; **22**(2).

45. President of the Republic of Colombia. Decreto 417 de 2020. 2020. https://www.funcionpublica.gov.co/eva/gestornormativo/norma.php?i=110334 (accessed 16 Feb 2025).

46. Congreso de La Republica Guatemala. INICIATIVA DE LEY DE RESCATE ECONÓMICO A LAS FAMILIAS POR LOS EFECTOS CAUSADOS POR EL COVID-19" (Economic Rescue Law Initiative for Families Affected by COVID-19). 2020. <https://participacion.congreso.gob.gt/wp-content/uploads/2020/04/5757.pdf> (accessed 16 February 2025).

47. SALUD; MD, PÚBLICA SDS. DECRETO 4 DECRETA ALERTA SANITARIA POR EL PERÍODO QUE SE SEÑALA Y OTORGA FACULTADES EXTRAORDINARIAS QUE INDICA POR EMERGENCIA DE SALUD PÚBLICA DE IMPORTANCIA INTERNACIONAL (ESPII) POR BROTE DEL NUEVO CORONAVIRUS (2019-NCOV). 2020. <https://www.bcn.cl/leychile/navegar?idNorma=1142163> (accessed 16 February 2025).

48. Vasques JDR, Peres AM, Straub M, de Souza TL. [Organization of healthcare systems to confront COVID-19: a scoping reviewOrganizacion de los sistemas de salud para hacer frente a la COVID-19: revision del alcance]. *Rev Panam Salud Publica* 2023; **47**: e38.

49. López-Arellano O, Delgado-Campos V. La transformación del sistema público de salud en la Ciudad de México. *Salud Pública de México* 2024; **66**(5 SEP-OCT).

50. Witter S. Health financing in fragile and post-conflict states: what do we know and what are the gaps? *Soc Sci Med* 2012; **75**(12): 2370-7.

51. Mora-Garcia CA, Pearson AA, Prado AM. Maintaining essential health services during a pandemic: lessons from Costa Rica's COVID-19 response. *BMJ Glob Health* 2024; **8**(Suppl 6).

52. Bigoni A, Malik AM, Tasca R, et al. Brazil's health system functionality amidst of the COVID-19 pandemic: An analysis of resilience. *Lancet Reg Health Am* 2022; **10**: 100222.

53. OECD. Fiscal Sustainability of Health Systems: Bridging Health and Finance Perspectives. Paris: OECD Publishing, 2015.

54. De Foo C, Verma M, Tan SY, et al. Health financing policies during the COVID-19 pandemic and implications for universal health care: a case study of 15 countries. *Lancet Glob Health* 2023; **11**(12): e1964-e77.

55. World Health Organization. Primary health care on the road to universal health coverage: 2019 global monitoring report., 2019.

56. Wolfrom L. Could insurance provide an alternative to fiscal support in crisis response? Paris: OECD, 2022.

57. Glass LT, Schlachta CM, Hawel JD, Elnahas AI, Alkhamesi NA. Cross-border healthcare: A review and applicability to North America during COVID-19. *Health Policy Open* 2022; **3**: 100064.

58. Hanson K, Brikci N, Erlangga D, et al. The Lancet Global Health Commission on financing primary health care: putting people at the centre. *Lancet Glob Health* 2022; **10**(5): e715-e72.

59. OECD. Dispelling Myths about Participatory Budgeting across Levels of Government. . Paris: OECD Publishing, 2022.

60. Wanwong Y, Sirinard N, Nopparattayaporn P, Poungkanta W, Putthasri W, Suphanchaimat R. Health Insurance for Undocumented Migrants: A Literature Review in Developed Countries. *JMAT* 2017.

61. Bilazarian A, Hovsepian V, Kueakomoldej S, Poghosyan L. A Systematic Review of Primary Care and Payment Models on Emergency Department Use in Patients Classified as High Need, High Cost. *J Emerg Nurs* 2021; **47**(5): 761-77 e3.

62. Diaconu K, Falconer J, Verbel A, Fretheim A, Witter S. Paying for performance to improve the delivery of health interventions in low- and middle-income countries. *Cochrane Database Syst Rev* 2021; **5**(5): CD007899.

63. Morgan SR, Chang AM, Alqatari M, Pines JM. Non-emergency department interventions to reduce ED utilization: a systematic review. *Acad Emerg Med* 2013; **20**(10): 969-85.

64. Lindner L, Lorenzoni L. Innovative providers’ payment models for promoting value-based health systems: Start small, prove value, and scale up, 2023.

65. Smela B, Toumi M, Świerk K, et al. Rapid literature review: definition and methodology. *Journal of market access & health policy* 2023; **11**(1): 2241234.

Supplementary Material 2. Commission methodology

This report used a mixed-methods approach to build three critical technical insights:

1. Organizing scheme for PHC resilience
2. Modelling of the cost of inaction
3. Recommendations on how to strengthen PHC resilience
4. **Organizing scheme for PHC resilience**

To build this organizing scheme, a two-step methodology was used. First, a literature review was conducted to identify existing frameworks addressing PHC and/or resilience. Secondly, a stakeholder consultation was also conducted to gather feedback on how to better improve the framework.

A.1. Literature review

*A.1.1. Data sources*

We searched the literature using following databases: MEDLINE and EMBASE using OVID; PubMed; HealthSystemsEvidence; LILACS; and Epistemonikos. We used a search strategy that includes terms related to PHC, resilience and specific disasters (see below). Additionally, we employed a complementary search strategy to capture relevant articles and frameworks by conducting searches in Google Scholar, and by asking experts to provide different frameworks. Through this process, articles that address a more general health system (without explicitly addressing PHC as part of it) approach were also included.

*A.1.2. Study selection*

The screening of titles and abstracts was carried out by two independent reviewers in 20% of the abstracts. The rest was conducted by a single reviewer. This process helps ensure that bias is minimized and that criteria are applied appropriately ^256^. Full text screening was conducted by a single reviewer. For screening articles, the online software Covidence was utilized. See below for PRISMA diagram of included studies.

Inclusion criteria, articles that:

- Address a PHC or essential public health functions topic.
- Create a conceptual framework to further understand the intersection between PHC and resilience.

Exclusion criteria, articles that:

- Do not review the existing literature (i.e., does not include primary studies), or do not state their eligibility criteria.
- Do not address a system-level topic (e.g., individual-level management of a health condition) that is relevant for PHC.
- Do not address a topic that is relevant for resiliency or a specific shock.

*A.1.3. Data extraction*Once identified eligible articles, a data extraction form was piloted using 10 articles to extract relevant data from the included studies. This form collected at least the following information:

- Citation
- Month and year of publication
- Whether the evidence synthesis produces a conceptual framework to understand the role of PHC in health system resilience
- Geographical scope in case it is applicable
- Study aim
- Main findings

*A.1.4. Data synthesis*

An iterative and critical qualitative synthesis was conducted. First, a policy framework was created based on existing resilience and PHC frameworks provided by experts. This framework was further complemented with additional articles that could add important insights into the existing framework. After defining critical domains of the organizing scheme, each one of those was disaggregated into smaller units that we called sub-domains.

A.2. Consultation with commissioners

Commissioners were consulted throughout the process. A first version of the scheme was presented in the kick-off meeting of the Commission in Washington, D.C., in March 2024. Critical feedback was gathered and incorporated into the framework at that stage.Later, multiple rounds of feedback were provided by working groups in each one of the domains, which was followed by critical feedback received during the second in-person meeting of the Commission in Panama in October 2024.

1. **Cost of inaction**

Please refer to the full paper to see the methods used for this section: <https://www.medrxiv.org/content/10.1101/2025.03.03.25323291v1.full.pdf>

1. **Recommendations on how to strengthen PHC resilience**

A multi-stage process was used to identify critical recommendations to strengthen PHC resilience in the region. First, an umbrella review of reviews was conducted to identify and assess the evidence on specific policy options. Secondly, a survey among commissioners was conducted to gather further experiences and innovations to strengthen PHC resilience. Thirdly, a qualitative study to deep diving on specific countries PHC resilience was also used to inform commissioners’ discussions. Fourthly, a series of structured meetings and consultations by the Commission allowed to craft the final set of recommendations. Finally, the recommendations formulated by commissioners were validated in three different countries

C.1. Umbrella review of the literature

Building on the methods outlined in the literature review above, we conducted a rapid umbrella review of reviews to identify and evaluate the evidence about interventions to strengthen PHC resilience.

*C.1.1. Data sources*

Same as A.1.1.

*C.1.2. Study selection*

The screening of titles and abstracts was carried out by two independent reviewers in 20% of the abstracts. The rest was conducted by a single reviewer. This process helped ensure that bias is minimized and that criteria are applied appropriately ^65^. Full text screening was conducted by a single reviewer. For screening articles, the online software Covidence was utilized.

Inclusion criteria, articles that:

- Address a PHC or essential public health functions topic.
- Incorporate interventions that is relevant for understanding the role of PHC in health systems resilience (i.e., preventing and preparing, alerting, managing and/or recovering from a shock).
- Are evidence syntheses of the literature

Exclusion criteria, articles that:

- Do not review the existing literature (i.e., does not include primary studies), or do not state their eligibility criteria.
- Do not address a system-level topic (e.g., individual-level management of a health condition) that is relevant for PHC.
- Do not address a topic that is relevant for resiliency or a specific shock.
- Do not include interventions as part of their scope.

*C.1.3. Data extraction*Once identified eligible articles, a data extraction form was piloted using 10 articles to extract relevant data from the included studies. This form collected at least the following information:

- Citation
- Month and year of publication
- Geographical scope in case it is applicable
- Study aim
- Interventions described
- Relevance for the domains of the conceptual framework
- Countries that were included in the included studies
- Main findings

*C.1.4. Quality appraisal*

Evidence syntheses included were appraised by a single reviewer, using the AMSTAR tool and were classified in low (score less than 4), medium (score between 4 and 7) and high (score 8 or higher).

*C.1.5. Data synthesis*

Using the six domains of the conceptual framework described above, the evidence syntheses included were split into these domains. Later, using a qualitative inductive and iterative coding of the interventions described in each synthesis, a classification of interventions was produced in each sub-domain of the framework. Additionally, a classification of types of question that each synthesis was used was also added in the presentation of the results. This classification was modified from an existing taxonomy of demand-driven questions and was concentrated in four main blocks: scoping a list of potential options; understanding how potential options work (mechanism of action); evaluating the expected impact of options and efforts to maximize it; and implementation and how to improve. Finally, a summary of findings was presented for each intervention-type of question. The evidence syntheses were also prioritized by their quality, in case multiple evidence syntheses were available for one single intervention-type of question.

C.2. Consultation survey

Using the findings of the umbrella review, a survey was circulated among commissioners in preparation for the second in-person meeting in October 2024.

*C.2.1. Participants*

All commissioners were invited to answer this survey. They were assigned to four working groups (integrated health services and essential public health functions, empower communities and participation, multisectoral policy and action, and financing), but they could also voluntarily answer the questions from other working groups.

*C.2.2. Data collection methods*

The survey was created to build on the findings of the umbrella review. Hence, commissioners were presented with the umbrella review findings and were asked multiple questions, including the country that they feel most comfortable with answering the questions. In each sub-domain, commissioners were asked to provide three actions that could support the implementation of each one of the interventions that were identified in the umbrella review. At the end, they were also invited to provide open comments to justify the rationale used to select these actions.

*C.2.3. Data analysis*

The actions collected in the survey were critically and inductively analyzed and were included in the materials that the commissioners used during the second in-person meeting in October 2024.

C.3. Deep dives

This is a qualitative study that followed a multiple case study design that was used to collect different innovations and the resilience level of a sample of countries in the region.

*C.3.1. Selection of countries*

The selection of countries was purposively sampled to achieve balanced geographic representation of different sub-regions of LAC (North and Central America, Caribbean, and South America). Additionally, we purposively sampled by the type of shock that the country has experienced in the last 10 years (epidemiological, conflict-related, natural disasters, demographic and economic crises), and the impact that they have had (classified as a devastating impact, and moderate or contained impact).As part of this sampling process, the selected countries include Brazil, Chile, Guatemala, Jamaica, and Mexico. For federal systems, sub-national governments were chosen based on the potential to facilitate access to interviewees—such as Bahia State in Brazil and Mexico City in Mexico—to enhance the feasibility and depth of the research.

*C.3.2. Data collection methods*

Two different data-collection methods were used. First, a documentary review of policy documents in each one of the countries was conducted. Later, this was complemented by a round of key-informant virtual interviews.

*C.3.2.1. Selection of participants*

Key informants were selected to ensure to ensure comprehensive representation across all domains of the framework (integrated health services and public health functions, empower communities and participation, multisectoral policy and action, and financing), as well as different roles (government, private sector, civil society and academia). The selection of key informants was conducted in conjunction with the national offices of PAHO and the World Bank. Semi-structured interviews were conducted using two different types of interview guides. One interview guide was used for stakeholders that do not have a specific thematic expertise, while the second interview guide was used for thematic expert interview (See Table 1 for a summary of these). During the interview, the idea was to collect information about the current state of each one of the domains of the framework, as well as critical innovations or actions that could strengthen PHC resilience in that country.

Table 1. Summarized interview guide

| 1. **Governance**   We understand governance as actions around political strategy and vision, policy implementation and regulation, stewardship and institutional structures, and system adaptation and learning.   - 1. From your experience in your country, what are the key governance actions at PHC level to prepare, prevent, alert, respond and recover from a shock?  1. **Integrated health services & public health functions**    1. We understand the domain of integrated health services and public health functions as actions around five key areas: empanelment; undertaking of essential public health functions; provision of essential healthcare services; care coordination, facility management, and community outreach; and quality and people-centeredness of care. 2. **Empowering communities**    1. We understand the domain of empower communities as actions around four main areas: community engagement; interculturality; communication and trust; and transparency and accountability. 3. **Multisectoral policy and action**   We understand multisectoral policy and action as actions involving three main areas: private health sector engagement (i.e., not-for-profit, and for-profit); non-health sector engagement; and environmental considerations.   - 1. From your experience in your country, what are the key multisectoral actions at PHC level to prepare, prevent, alert, respond and recover from a shock  1. **Finance**   We understand finance as actions around 4 main areas: financial governance; revenue raising and pooling of resources; management and allocation of funds; and purchasing and payment mechanisms.   - 1. From your experience in your country, what are the key financial actions at PHC level to prepare, prevent, alert, respond and recover from a shock?  1. **Generation & distribution of inputs/ resources to deliver primary care and public health services**   We understand this domain as a group of multiple actions around 7 areas: governance specific to the generation & distribution of inputs/ resources to deliver primary care and public health services; health workforce; infrastructure and medical equipment; pharmaceuticals, vaccines and supplies; digital technologies for health (i.e., primary care and EPHF); health information systems & surveillance; and monitoring and evaluation.   - 1. From your experience in your country, what are the key actions related to inputs/resources at PHC level to prepare, prevent, alert, respond and recover from a shock?  1. **Closing questions:**    1. Is there anything else that you would like to add regarding PHC resilience or to any of the topics that we have covered during the interview?    2. Would you like to comment on how equitably the actions that we have discussed today have been implemented across the country? |
| --- |

*C.3.3. Data analysis*

A qualitative, critical analysis was conducted by identifying common themes across the documentary review and the insights provided by the key informant interviews. Each theme was assigned to the specific sub-domains of the framework, and innovations or critical actions were also reported and included as materials for discussion during the second in-person meeting of the Commission.

C.4. Structured meetings

A series of structured meetings and discussions were held among commissioners to formulate the recommendations. Each one of these discussions were informed by previous technical products that were described above. Three main types of events can be outlined.

*C.4.1. In-person meetings*

Two in-person meetings were held by commissioners: one in March and another in October 2024. In each one of these meetings, general and working group discussions were held among commissioners. The meetings were also enriched by critical insights. During the first meeting in March 2024 in Washington, D.C., the original conceptual framework was presented to receive feedback from commissioners, and working groups were created to outline the scope of their work. During the second meeting in October 2024 in Panama, the findings of the umbrella review, survey and deep dives were presented to commissioners, and the first draft of the recommendations was produced through multiple general and working group discussions.

*C.4.2. Virtual meetings by working group*

As the commissioners split into four different working groups, each one of them met regularly over 2024 to continue working over the recommendations, as well as finding policy options that would be appropriate to achieve the objectives of the Commission.

*C.4.3. Writing retreat*

During February 2025, the co-chairs of the Commission met in person to finalize and complete the report. During this meeting, a virtual meeting with each working group was also held to receive critical feedback from commissioners.

C.5. Validation of the recommendations

The recommendations that were formulated by commissioners were also validated in three different countries: Brazil, Chile and Jamaica.

*C.5.1. Data collection methods*

Two different data collection methods were used to validate the recommendations. First, a survey outlining the recommendations structured by domain, and the specific policy options that were suggested by commissioners was circulated among participants. Here, they were asked to rank their opinions about both the relevance and the feasibility of implementing each one of the policy options. Likert-5 scales were used to rank the relevance and feasibility. Furthermore, participants could choose one or more domains when answering the survey, and they could also provide open comments to justify their ratings. Secondly, a virtual workshop was conducted with multiple national stakeholders. Here, participants were asked to answer the survey questions, and an structured discussion was followed to get any feedback related to the relevance and feasibility of the recommendations. The participants were split in break-out rooms, so they could concentrate in one domain.

*C.5.2. Participants*

Participants were invited to answer the survey and join the workshop following a purposive sampling strategy that would allow geographic representation across the country, as well as diversity in the role type (government, program managers, citizens, academia).

*C.5.3. Data analysis*

The feedback received on the survey was used to inform the discussion during the workshop. Hence, the discussions were focused on the questions that were classified as having some level of irrelevance or infeasibility by some participants. Later, the insights collected from the open-ended questions of the survey, and the workshops were descriptively analyzed, and introduced any specific modification needed to the Commission recommendations.

**Search Strategy (run in PubMed on 3 April 2024):**

| # | Search strategy | Number of results |
| --- | --- | --- |
| **PRIMARY CARE TERMS:** | | |
| 1 | "primary healthcare"[Title/Abstract:~1] OR "primary health care"[Title/Abstract:~1] OR "primary care"[Title/Abstract:~2] OR "primary health"[Title/Abstract:~1] OR "primary practice"[Title/Abstract:~2] OR "primary practicing"[Title/Abstract:~2] OR "primary practice"[Title/Abstract:~2] | 202,129 |
| 2 | "public health function*"[Title/Abstract] OR "functions of public health"[Title/Abstract] OR "essential public health function*"[Title/Abstract] | 395 |
| 3 | "Primary Health Care"[Mesh] | 196,172 |
| 4 | #1 OR #2 OR #3 | 322,600 |
| **RESILIENCE TERMS** | | |
| 5 | emergenc*[Title/Abstract] OR disaster*[Title/Abstract] OR shock*[Title/Abstract] OR crisis[Title/Abstract] OR crises[Title/Abstract] OR catastroph*[Title/Abstract] OR "mass casualty"[Title/Abstract:~1] OR "mass casualties"[Title/Abstract:~1] OR hazard*[Title/Abstract] OR calamit*[Title/Abstract] OR resilien*[Title/Abstract] | 1,299,587 |
| 6 | "Disasters"[Mesh] OR "Emergencies"[Mesh] | 112,850 |
| 7 | #5 OR #6 | 1,355,149 |
| **SPECIFIC DISASTERS** | | |
| 8 | shooting[Title/Abstract] OR war[Title/Abstract] OR wars[Title/Abstract] OR avalanche*[Title/Abstract] OR storm*[Title/Abstract] OR cyclone*[Title/Abstract] OR drought*[Title/Abstract] OR earthquake*[Title/Abstract] OR flood*[Title/Abstract] OR landslide*[Title/Abstract] OR tornado*[Title/Abstract] OR hurricane*[Title/Abstract] OR wildfire*[Title/Abstract] OR typhoon*[Title/Abstract] OR tsunami*[Title/Abstract] OR rockslid*[Title/Abstract] OR mudslid*[Title/Abstract] OR (tidal[Title/Abstract] AND wave*[Title/Abstract]) OR tidalwave*[Title/Abstract] OR conflict*[Title/Abstract] OR outbreak*[Title/Abstract] OR pandemic*[Title/Abstract] OR genocide[Title/Abstract] OR cataclysm*[Title/Abstract] OR volcan*[Title/Abstract] OR terroris*[Title/Abstract] OR accident*[Title/Abstract] OR heatwav*[Title/Abstract] OR bioterroris*[Title/Abstract] | 821,974 |
| 9 | Coronavir*[Title/Abstract] OR 2019-nCoV[Title/Abstract] OR 2019nCoV[Title/Abstract] OR nCoV[Title/Abstract] OR SARS-CoV-2[Title/Abstract] OR COVID19[Title/Abstract] OR COVID-19 OR "severe acute respiratory syndrome"[Title/Abstract] OR SARS[Title/Abstract] | 438,263 |
| 10 | #8 OR #9 | 1,038,310 |
| **COMBINATION TERMS** | | |
| 11 | #7 OR #10 | 2,218,505 |
| 12 | #11 AND #4 | 45,104 |
| **FILTER FOR EVIDENCE SYNTHESES** | | |
| 13 | #12 AND (systematicreview[Filter]) | 949 |
| 14 | #12 AND (review[Filter]) | 3,726 |
| 15 | #12 AND (meta-analysis[Filter]) | 324 |
| 16 | #13 OR #14 OR #15 | 4,212 |

**Search Strategy (run in OVID on 3 April 2024):**

| # | Search strategy | Number of results in Medline | Number of results in EMBASE |
| --- | --- | --- | --- |
| **PRIMARY CARE TERMS:** | | |  |
| 1 | (primary adj2 (healthcare or health or care or practice or practicing)).ti,ab. | 185,914 | 251,027 |
| 2 | ("public health function*" or "functions of public health" or "essential public health function*").ti,ab. | 509 | 510 |
| 3 | exp primary health care/ | 196,233 | 216,094 |
| 4 | 1 or 2 or 3 | 309,028 | 311,514 |
| **RESILIENCE TERMS** | | |  |
| 5 | (emergenc* or disaster* OR shock* OR crisis OR crises OR catastroph* OR "mass casualty" OR "mass casualties" OR hazard* OR calamit* OR resilien*).ti,ab | 1,279,431 | 1,711,750 |
| 6 | exp disasters/ or exp emergencies/ | 112,875 | 91,930 |
| 7 | 5 or 6 | 1,335,790 | 1,731,732 |
| **SPECIFIC DISASTERS** | | |  |
| 8 | (shooting or war or wars or avalanche* or storm* or cyclone* or drought* or earthquake* or flood* or landslide* or tornado* or hurricane* or wildfire* or typhoon* or tsunami* or rockslid* or mudslid* or (tidal adj3 wave*) or tidalwave* or conflict* or outbreak* or pandemic* or genocide or cataclysm* or volcan* or terroris* or accident* or heatwav* or bioterroris*).ti,ab. | 808,703 | 957,095 |
| 9 | (coronavir* or 2019-nCoV or 2019nCoV or nCoV or SARS-CoV-2 or COVID19 or COVID-19 or "severe acute respiratory syndrome" or SARS).ti,ab. | 419,104 | 475,358 |
| 10 | 8 or 9 | 1,012,925 | 1,197,215 |
| **COMBINATION TERMS** | | |  |
| 11 | 7 or 10 | 2,179,884 | 2,752,252 |
| 12 | 4 and 11 | 42,113 | 40,432 |
| **FILTER FOR EVIDENCE SYNTHESES** | | |  |
| 13 | (meta-analysis or "meta analysis" or metanalysis).ti,ab. | 257,463 | 325,703 |
| 14 | ((systematic or scoping or qualitative or quantitative or evidence or critical or mapping or integrative or state-of-the-art or literature or umbrella) adj3 (review or reviews or synthesis or overview)).ti,ab. | 748,318 | 883,266 |
| 15 | 13 or 14 | 857,681 | 1,029,996 |
| 16 | (meta-synthesis or meta-summary or meta-review).ti,ab. | 2,132 | 2,336 |
| 17 | 15 or 16 | 857,992 | 1,030,340 |
| 18 | 12 and 17 | 1,916 | 1,856 |

PRISMA Diagram for Umbrella Review

**Identification of studies via other methods**

**Identification of studies via databases and registers**

Records identified from:

Google Scholar searches

**(n = 1600**)

Records removed *before screening*:

Duplicate records removed (**n = 2527**)

Records identified from Databases (**n = 8296**)

Medline (OVID) (n= 1916)

EMBASE (OVID) (n= 1856)

PubMed (n= 4212)

LILACS (n= 312)

**Identification**

Records screened **(n = 5769)**

In duplicate (n = 1039)

Single reviewer (n = 4730)

Records excluded

(**n =4870**)

Reports not retrieved

**(n = 0)**

Reports sought for retrieval

**(n = 34)**

Reports sought for retrieval

(**n = 899**)

Reports not retrieved

(**n = 0)**

**Screening**

Reports excluded (n= 11)

Not evidence synthesis

(n = 8)

Not relevant for PHC systems (n = 2)

Not relevant for resilience

(n = 1)

No interventions

(n = 0)

Reports assessed for eligibility

**(n = 34)**

Reports excluded **(n= 646)**

Not evidence synthesis

(n = 240)

Not relevant for PHC systems (n = 148)

Not relevant for resilience

(n = 165)

No interventions

(n = 93)

Reports assessed for eligibility

**(n = 899)**

Studies included in review

**(n = 276)**

**Included**

Supplementary Material 3. Research gaps organized by PHC resilience domain and sub-domain

| **Integrated Service Delivery and Essential Public Health Functions** |
| --- |
| **Models of primary care that provide services for all** |
| **RESEARCH GAPS:**   - Limited evidence on inclusive PHC models that promote equity, continuity, and adaptability across diverse LAC settings. Evidence on the integration of public health functions, such as surveillance, health promotion, or intersectoral coordination, into PHC remains scattered and varies widely in approach. Many studies describe efforts at coordination, but few assess their effectiveness in terms of PHC performance, resilience, or health equity. Important system-level outcomes (e.g., early detection, service continuity during shocks) are often not tracked, and many findings come from high-income contexts, limiting their relevance to LAC PHC structures. - Across settings like Chile, Brazil, and Argentina, PHC models targeting adults and older adults have advanced through empanelment systems, screening protocols, and multicomponent approaches designed to extend coverage, improve accountability, and organize service delivery around populations. These models were especially relevant in rural or underserved areas, offering structured follow-up and continuity. Compared to more fragmented or facility-centered approaches, they demonstrated improved population tracking and a shift toward proactive service delivery. Despite promising outcomes, many initiatives remain context-specific, and comparative, longitudinal evaluations are still limited, making it difficult to fully assess their potential contribution to resilience during health shocks. |
| **Integrating essential public health functions in primary care** |
| **RESEARCH GAPS:**   - Fragmented evidence on how surveillance, promotion, and protection are operationalized through PHC; integration is often implied but not assessed. Evidence on the integration of public health functions, such as surveillance, health promotion, or intersectoral coordination, into PHC remains scattered and varies widely in approach. Many studies describe efforts at coordination, but few assess their effectiveness in terms of PHC performance, resilience, or health equity. Important system-level outcomes (e.g., early detection, service continuity during shocks) are often not tracked, and many findings come from high-income contexts, limiting their relevance to LAC PHC structures. - In the LAC region, as well as in other LMICs, several initiatives have attempted to integrate essential public health functions, such as surveillance, health promotion, and intersectoral collaboration, directly into PHC systems. These interventions aim to strengthen health systems’ capacity to detect and respond to health threats while promoting preventive care at the community level. Compared to fragmented models where public health and PHC operate in isolation, integration has been associated with improved alignment of services and greater efficiency in outbreak detection and prevention. Nevertheless, many of these strategies remain unevenly applied and inconsistently evaluated, making it difficult to assess their long-term contribution to PHC resilience and health security. |
| **Maintaining essential health care services in primary care** |
| **RESEARCH GAPS:**   - Focus of existing literature is primarily on crisis response; continuity of essential PHC services in non-crisis periods is less explored. Designs are often descriptive, and interventions vary across countries, leading to inconsistent findings. Moreover, interventions are rarely tracked over sufficient time periods to evaluate sustainability, and core outcomes like chronic disease management, trust, or patient retention are often missing. - Countries like Brazil, Colombia, and Costa Rica have implemented service reorganization strategies, such as telehealth, mobile outreach, and facility-level adaptation, to maintain essential PHC services during crises like COVID-19 and natural disasters. These adaptations were critical for sustaining care in the face of mobility restrictions and resource constraints. Compared to PHC systems that lacked planning for service continuity, these interventions helped reduce service disruption and maintain patient satisfaction. However, most findings stem from short-term observations, and long-term effects on equity, chronic disease management, or system resilience remain underexplored. More research is needed to assess how these adjustments can be institutionalized as part of routine PHC preparedness. |
| **Ensuring that the specialized health care network engages appropriately with primary care** |
| **RESEARCH GAPS:**   - Scarce documentation of referral pathways, feedback loops, and coordination mechanisms between PHC and specialized services. There is limited evidence on how coordination affects patient outcomes, efficiency, or system resilience during shocks. Moreover, many interventions are designed for high-income settings or tertiary care systems, reducing their applicability to PHC-focused contexts in the region. - Efforts to improve coordination between PHC and the specialized health care network have been documented in several countries, notably Colombia, as well as in Mexico, Uruguay, Jamaica, and to a lesser extent, Peru and Argentina. These initiatives have included digital referral platforms, shared chronic care pathways, co-management protocols, and post-discharge follow-up mechanisms aimed at ensuring smoother patient transitions between levels of care. In Colombia, particularly within post-conflict and rural areas, structured contracting and inter-level coordination have been central to efforts to strengthen PHC resilience. Compared to systems where referrals are informal or delayed, these approaches have shown promise in reducing fragmentation, improving continuity, and ensuring timelier specialist care. However, the evidence base remains limited, with most examples drawn from small-scale pilots or single-country case studies. There is a lack of rigorous evaluations on outcomes such as patient retention, long-term care quality, and system responsiveness during health shocks, making this a key area for further research and regional learning. |

| **Community participation/empowerment** |
| --- |
| **Community engagement** |
| **RESEARCH GAPS:**   - Although a range of community health worker (CHW) and peer-led interventions are described, particularly in relation to Indigenous populations, digital health literacy, and discharge planning, the literature lacks robust comparative evaluation of how these models integrate into PHC systems over time. Most studies are descriptive and context-specific (e.g., in Haiti or among Indigenous populations), with few head-to-head comparisons or multi-country analyses. This reflects gaps such as limited number of studies, consistency unknown and results not applicable to broader settings. There is a clear need for longitudinal studies assessing CHW effectiveness within resilient PHC across diverse LAC and LMIC contexts. - In settings such as Chile, Haiti, Colombia, and El Salvador, CHW programs and culturally tailored outreach efforts have been deployed to support refugees, Indigenous populations, marginalized urban and rural communities, vulnerable groups, and children under five—particularly in disaster-affected contexts. These interventions are often compared to more traditional, facility-based or top-down response models, with community-integrated approaches showing advantages in building trust, improving continuity of care, enhancing outreach, and contributing to emergency preparedness. Reported outcomes include improved self-care and reductions in emergency department use. While CHW programs are widely endorsed, especially in high-income countries, their implementation mechanisms and long-term integration into health systems remain under-evaluated in LMICs. |
| **Interculturality** |
| **RESEARCH GAPS:**   - Cultural adaptations are highly localized; frameworks for evaluation and replication are scarce. Intercultural approaches are integral to making PHC more inclusive and contextually appropriate, especially for Indigenous or minority populations. However, the connection to resilience and shocks is rarely made explicit. Most studies describe cultural adaptations as standalone interventions rather than components of system-wide strategies for maintaining service delivery under stress. As such, the role of interculturality in building trust, reducing vulnerabilities, or enhancing PHC system responsiveness during emergencies remains insufficiently explored. There is a clear opportunity to better integrate intercultural strategies into resilience frameworks that account for diverse sociocultural realities across LAC. - In some countries, for example Colombia, interventions targeting migrants, Indigenous peoples, and refugees have focused on discharge planning and care models that integrate cultural knowledge and community-based supports. Compared to standard care models without cultural adaptation, these approaches are associated with improved patient satisfaction, reduced hospital readmissions, and better alignment of care with patients' cultural beliefs. Although positive outcomes have been reported, particularly in terms of patient experience, most of the evidence is qualitative, with limited data on cost-effectiveness, scalability, or long-term sustainability. |
| **Communication and trust** |
| **RESEARCH GAPS:**   - Communication is essential to both PHC quality and system responsiveness, particularly during health shocks. Despite this, the reviewed evidence points to a gap in evaluated, context-specific communication strategies that are designed for PHC platforms during emergencies. Messaging tailored to marginalized groups or designed to build long-term trust is often mentioned, but not rigorously tested. The role of communication in building health system resilience, e.g., by reducing misinformation, supporting care continuity, or strengthening community relationships, remains under-theorized and under-evaluated, particularly within PHC delivery contexts. - In countries such as Brazil, efforts to strengthen community trust and communication among the general population, Indigenous groups, and refugees have included risk communication campaigns, capacity building, and trust-building initiatives involving community influencers and local leaders. These approaches are often contrasted with top-down official messaging, with co-created, community-driven strategies demonstrating potential to improve risk awareness, institutional trust, adherence to public health guidance, and proactive health-seeking behavior. Although such interventions were highly relevant during the COVID-19 pandemic, few studies have isolated the specific impact of communication modalities, and there is a notable absence of longitudinal data or robust impact evaluations, particularly across diverse and underserved population groups. |
| **Transparency and accountability** |
| **RESEARCH GAPS:**   - Transparency and accountability are widely recognized as essential components of effective health governance, yet there is limited evidence on how these mechanisms directly contribute to PHC resilience or responsiveness, particularly during health emergencies. There is limited evidence on how these mechanisms directly enhance PHC resilience or responsiveness. |

| **Multi-sectoral action** |
| --- |
| **Private health sector engagement (i.e., non-for-profit & for-profit.).** |
| **RESEARCH GAPS:**   - While some reviews highlight coordination with non-profit and for-profit health actors, particularly during COVID-19 and Ebola, most studies are descriptive and situated in fragile or humanitarian settings (e.g., post-conflict or rural zones). The role of private actors in supporting or undermining PHC resilience during shocks is rarely evaluated systematically. Furthermore, outcomes related to service continuity, system responsiveness, and community-level integration remain under-explored, especially in LMIC contexts. Few studies measure whether private sector engagement strengthens PHC systems' ability to absorb, adapt, or transform during crises. - In rural, remote, and conflict-affected populations across countries like Brazil, Cuba, Ecuador, Peru, and Argentina, private sector engagement during public health emergencies has included contracting NGOs, developing surge capacity coordination, and integrating private actors into preparedness planning. These approaches, compared to public-only delivery, showed potential to expand coverage, sustain service continuity, and improve innovation in delivery. Evidence from moderate- and low-quality syntheses suggests that coordination and accountability mechanisms were key, yet most studies lacked evaluation of long-term integration, financial transparency, or sustained PHC resilience. The connection to PHC functions and shock preparedness is recognized but under-assessed, with most findings descriptive and concentrated in fragile or humanitarian settings. |
| **Private for and not-for-profit health sector engagement** |
| **RESEARCH GAPS:**   - Intersectoral collaboration, across education, transport, Indigenous governance, and other areas, is frequently mentioned as a principle of Health in All Policies or emergency response planning. However, these linkages are rarely assessed for their specific contribution to PHC functionality or resilience-building. Evaluations often focus on governance processes (e.g., formation of task forces or communication networks) without examining downstream impacts on PHC service delivery, shock preparedness, or recovery. Differences in institutional arrangements across countries also lead to variation in implementation quality and unclear pathways of impact. - Populations benefiting from non-health sector engagement during health shocks include Indigenous communities and underserved rural groups. Interventions have focused on multisectoral governance, emergency risk communication, and intergovernmental coordination, particularly in countries like Brazil, Cuba, Ecuador, and Australia. Compared to siloed sector responses, whole-of-system models were associated with improved interagency collaboration and better continuity of PHC services during crises. However, implementation challenges, such as lack of trust, unclear mandates, and coordination gaps, were frequently reported. While linkages to PHC system resilience are frequently noted, the evidence base lacks consistent evaluation of outcomes like equity, efficiency, or long-term integration, and few studies rigorously assess impacts on shock preparedness. |
| **Environmental considerations** |
| **RESEARCH GAPS:**   - Environmental considerations are conceptually linked to resilience but are rarely evaluated in relation to PHC or shock scenarios. Most interventions such as greening clinics or provider training originate from high-income settings and lack follow-up data, limiting their relevance to PHC systems in low- and middle-income countries. The connection to resilience is implied, but the impact on PHC functionality during environmental or climate-related shocks remains underexplored, resulting in limited and non-generalizable evidence. - Across Brazil, Argentina, Ecuador, and Peru, environmental interventions related to PHC resilience have included One Health initiatives, provider training in sustainability, and climate-conscious clinic operations. Compared to PHC models lacking environmental preparedness, these interventions aim to improve risk awareness, environmental adaptation, and systemic integration. Although moderate-quality reviews suggest conceptual alignment with resilience and health security goals, most studies are exploratory and lack follow-up data. Tools and training programs remain under-implemented and their impact on PHC continuity or adaptability during climate-related shocks is not yet evidenced. As such, environmental preparedness remains an emerging but weakly operationalized domain within PHC resilience strategies. |

| **Financing** |
| --- |
| **Financial governance** |
| **RESEARCH GAPS:**   - High heterogeneity and lack of systematic evaluations of financial coordination mechanisms before, during, and after crises. Very few provide comparative impact assessments or mechanisms to improve long-term financial resilience. The literature highlights regulatory and legal mechanisms used during crises, but these are rarely connected explicitly to strengthening PHC. The concept of resilience is typically framed as a reactive capacity during emergencies rather than as a proactive function embedded in PHC systems. - In the context of health systems responding to emergencies such as natural disasters and pandemics, several countries, including Chile, Brazil, India, Australia, and China, have implemented legal frameworks, emergency decrees, budgetary reallocations, and preparedness funds as key interventions. Comparing systems with versus without such legal preparedness protocols reveals differences in PHC responsiveness, intersectoral coordination, and the agility of funding mechanisms during crises. These interventions are aimed at enhancing the ability of PHC systems to respond rapidly and effectively to shocks by ensuring timely resource mobilization and coordinated governance structures. |
| **Revenue raising and pooling resources** |
| **RESEARCH GAPS:**   - Literature is sparse and mostly relates to humanitarian or donor funding in fragile/post-conflict contexts. Studies on revenue raising and pooling largely focus on the role of international aid or insurance expansion during emergencies, particularly in fragile or post-conflict settings. However, these mechanisms are not clearly linked to PHC structures or outcomes, and their implications for resilience are not systematically analyzed. - Among migrant, conflict-affected, and undocumented populations, countries such as Germany, France, Italy, Colombia, and Argentina have employed interventions including humanitarian aid, state-led insurance mechanisms, and pooled funding schemes to enhance health coverage during emergencies. Comparing contexts without versus with such support highlights improvements in access equity and financial protection for vulnerable groups. |
| **Management and allocation of funds** |
| **RESEARCH GAPS:**   - Findings related to benefit package adjustments, co-payment waivers, or insurance expansion during COVID-19 are reported, but outcome measures are inconsistent and context-sensitive. There is limited comparative research on how resource allocation shifts impact PHC continuity and equity during crises. Discussions often focus on financial protection or system-level resource flows, without specifying how allocations enable PHC to respond to or recover from shocks. There is also limited evidence on whether these changes are sustained beyond the crisis period or institutionalized as part of broader resilience strategies. - Countries such as the USA, Canada, Colombia, and Uruguay have implemented waivers, equity funds, co-payment adjustments, and tiered reimbursement systems as emergency financial modifications. Compared to standard cost-sharing models, these interventions have shown to improve service utilization, reduce emergency department visits, and enhance affordability. |
| **Purchasing and payment mechanisms** |
| **RESEARCH GAPS:**   - Evidence is diverse in design, few long-term outcome studies on payment reforms. Some high-quality syntheses are available; integration with digital payment infrastructure needs study. Studies remain narrowly focused on utilization metrics or immediate cost efficiency, without examining how payment mechanisms shape PHC's adaptability, workforce retention, or capacity to absorb shocks. Moreover, few analyses compare how payment reforms perform under different types of shocks, such as pandemics versus climate-related events, leaving a gap in understanding their role in supporting resilient PHC. - In the area of purchasing and payment mechanisms, countries such as Canada, Argentina, Brazil, and Costa Rica have introduced performance-based financing, capitation models, and virtual care payment schemes targeting PHC providers and patients in high-need categories. Compared to traditional fee-for-service models, these alternative payment approaches are associated with improved service continuity, greater cost efficiency, and reductions in emergency department utilization. |

Supplementary Material 4. Prioritized policy options in Chile and the Dominican Republic

Table 1. Prioritized policy options in Chile and their rationale.

| **INTEGRATED HEALTH SERVICES AND ESSENTIAL PUBLIC HEALTH FUNCTIONS** | | |
| --- | --- | --- |
| **Domain** | **Prioritized policy options** | **Rationale for their prioritization** |
| Models of PHC that provide services for all | Mechanisms to understand user experience to improve person-centeredness during care. | Despite user experience is yearly measured in PHC facilities across the country, it could be strengthened by conducting them specifically during or after shocks. |
| Integrating EPHFs into PHC | Using PHC units as sentinel sites for the early identification of outbreaks and other risks. Regular health situation assessments conducted by PHC to detect risk factors and vulnerabilities | Sentinel sites are present throughout the country, but they are not strategically determined to take advantage of the widespread PHC country network. |
| Maintaining essential healthcare services at the PHC level | Well established and tested emergency response plans to keep PHC facilities open and maintaining access to essential services during a shock. | Although emergency response plans must exist at the municipality level, these are not regularly tested. In addition, there is a gap for the PHC of the Healthcare Districts. |
| Ensuring that the specialized healthcare network engages appropriately with PHC | Systems to provide appropriate support referral systems to other level facilities including patient transport systems, information flows, digital technology to support referral systems, waiting-list management, among others. | The systems to maintain adequate referral channels during and after emergencies can be improved, particularly with the use of digital tools (e.g. interoperability of clinical records between levels of care). |
| **EMPOWERING COMMUNITIES** | | |
| **Domain** | **Prioritized policy options** | **Rationale for their prioritization** |
| Community engagement | Mapping of actors, knowledge systems and capacities to prepare for emergencies. | This is currently only done in the municipalities that joined the Universal PHC program and not with a resilience focus, so it could be further developed and scaled up nationally. |
| Interculturality | Continuous education and healthcare workers training programs that formally integrate interculturality. | Although interculturality training programs are developed there are still gaps in knowledge about how to prepare and respond to shocks with an interculturality perspective. |
| Communication and trust | Co-creation of the design and delivery of health and risk messages with trusted community representatives. | Messaging design and delivery does not regularly or formally include the participation of trusted community representatives. |
| Transparency and accountability | Having regular meetings between communities and PHC providers/authorities (at least quarterly) to plan and implement emergency programs | Emergency plans at local level are not developed and regularly revised with communities. |
| **MULTISECTORAL ACTION** | | |
| **Domain** | **Prioritized policy options** | **Rationale for their prioritization** |
| Private health sector engagement | Capacity assessment (e.g. infrastructure, equipment) of the private sector and its complementarity with the public sector to increase surge capacity | While the capacity at both public and private sector has been identified to respond to external shocks, convening private actors to participate in the response to external shocks has worked in a more case-by-case basis, which could be more institutionalized in preparing for future emergencies. |
|  | Convening private and public actors to plan, respond and learn to sustain PHC essential services during shocks, including contracting-out and other types of PPPs |  |
| Intersectoral engagement | National and local plans to prepare for emergencies that identifies actions at PHC level and network to provide non-health services (e.g. financial support, food aids, subsidies, guaranteed minimum income, etc.). | While there are existing intersectoral plans to prepare for external shocks, these plans do not always consider the role of PHC in providing non-health services, and the potential role that non-health workers could have in providing healthcare services. |
|  | Leveraging the role of non-health workers (e.g. police, army) and community members. |  |
| Governance, production and use of data | Creating vulnerability maps and action plans at the local level (geographical, disadvantaged groups) | Important developments have been conducted in this area to use multiple sources of data to create interactive maps and dashboards. However, there is still an important number of data sources that could be linked to produce intersectoral dashboards and maps to inform decision-making to prepare and respond to external shocks. |
|  | Building intersectoral dashboards with key indicators at the national and local levels |  |
| Environmental considerations | Building and refurbishing PHC facilities with a green perspective (e.g. site and context, day lighting, natural ventilation, solar shading, insulation, roof and wall reflectivity, exterior planting, solar panels, etc.) | While some specific cases have been conducted in some municipalities, there is not a full institutionalized plan to overarchingly embrace environmental considerations in PHC. |
| **FINANCING** | | |
| **Domain** | **Prioritized policy options** | **Rationale for their prioritization** |
| Financial governance | Emergency previously drafted financial decrees with clear protocols that enable targeted funding for urgent PHC activities (e.g., community health outreach, vaccinations, and essential services) | A staggered system to use resources during emergencies is available in Chile, but the availability of protocols and financial decrees is limited in preparing for external shocks. |
| Revenue raising and pooling of resources (where the money come from) | Enabling efficient transfers from national-level governments to support local governments in managing emergencies | Efficient transfers across government levels and free-of-charge PHC services are available during emergencies, but financial protections are discussed once the emergency has started and not in the preparation stage. |
| Management and allocation of funds | Benefit package for health crisis management that incorporates planning and forecasting to enable flexible resource allocation, adaptable to evolving needs during emergencies | No a-priori benefit package and accountability systems are in place to support a more standardized and organized financial response during external shocks. |
| Purchasing and payment mechanisms | Risk assessments to strengthen supply-chain management during emergencies. | Purchasing mechanisms are not adjusted by external shock risks, while assessments are needed to strengthen and making supply chains more resilient. |

Table 2. Prioritized policy options in the Dominican Republic and their rationale.

| **INTEGRATED HEALTH SERVICES AND ESSENTIAL PUBLIC HEALTH FUNCTIONS** | | |
| --- | --- | --- |
| **Domain** | **Prioritized policy options** | **Rationale for their prioritization** |
| Models of PHC that provide services for all | Interprofessional teams (whose composition may vary by local conditions, but may be composed of medical doctors, nurses, community health workers, and social workers) with complementary skillsets who can provide collaborative person-centred care. | PHC teams normally only have a doctor, nurse and community health workers. While outreach efforts are currently present, they are not necessarily connected with broader efforts in preparing and responding to external shocks. |
|  | Registries /enrolment/mapping to identify populations within catchment areas, especially vulnerable/priority populations (e.g., migrants, elderly including those in nursing homes, people with disabilities) and screening of population (e.g. by gender, age, existing conditions etc.) for potential and existent vulnerabilities. | No form of systematic empanelment is currently in place. |
| Integrating EPHFs into PHC | Specialist epidemiological teams (e.g., from the Health Department, or National Public Health Institutes) provide support to primary care facilities for health surveillance | While the existing PHC facilities cannot contribute to epidemiological tasks, having specialized teams supporting PHC would enable to strengthen connections between levels of care. |
|  | Regular health situation assessments conducted by PHC to detect risk factors and vulnerabilities | Regular assessments to identify PHC risks and vulnerabilities in preparing to external shocks are not currently conducted. |
| Maintaining essential healthcare services at the PHC level | Well established and tested emergency response plans to keep PHC facilities open and maintaining access to essential services during a shock. | No existing emergency plans for PHC are available in the country. |
|  | Ensuring that primary care workforce is adequate to provide essential health services, including adequate health professionals planning, training health workers, providing incentives, mental health care services for health professionals; and established systems for communications. | While the existing health workforce is one of the key aspects of prevention, preparing and responding to emergencies, the country needs to strengthen its strategy to have critical capacity to respond to external shocks. |
| Ensuring that the specialized healthcare network engages appropriately with PHC | Well-trained health professionals that provide effective and quality clinical services in primary care avoiding unnecessary referrals | The connection of PHC with specialized services is probably an area that the country is strengthening. This is especially critical as during emergencies, it is even more relevant avoiding unnecessary referrals from PHC. |
|  | Supplying PHC facilities with inputs, equipment, technologies, medicines to provide effective and quality clinical services in PHC avoiding unnecessary referrals |  |
| **EMPOWERING COMMUNITIES** | | |
| **Domain** | **Prioritized policy options** | **Rationale for their prioritization** |
| Community engagement | Legal and regulatory frameworks that institutionalize opportunities for community participation (e.g., recurring community input into local plans to prepare and respond to public health emergencies). | The formal institutionalization of social participation to prevent, prepare and respond to emergencies needs to be strengthened in the country, particularly in how the current legal frameworks are implemented. |
|  | Inclusive participatory oversight that ensures application of legal and regulatory frameworks as part of transparency and accountability mechanisms at all levels of decision-making. | The regular and institutional gathering of PHC authorities and communities is not an institutionalized strategy as part of the efforts conducted to prevent, prepare and respond to emergencies. |
| Interculturality | Establishing and financing intercultural delivery of primary care services with full and effective participation of communities | Encouraging community participation and the training of healthcare workers in intercultural issues (including migration) are key priorities to better prepare and respond to emergencies. |
|  | Continuous education and healthcare workers training programs that formally integrate interculturality |  |
| Communication and trust | Creating communication materials that will be culturally appropriate and available in local languages to deliver health and risk messages | To improve the current strategy to prepare and respond to emergencies, communication materials and multiple channels to communicate to reach diverse populations (including migrants) are key priorities to strengthening in the country. |
|  | Using multiple channels to communicate health and risk messages to reach diverse populations |  |
| **MULTISECTORAL ACTION** | | |
| **Domain** | **Prioritized policy options** | **Rationale for their prioritization** |
| Private health sector engagement | Regulatory instruments (legislation, plans, bylaws, etc.) for public-private collaboration with roles, contracting-out rules, funding and oversight to articulate service provision. | Both the existence of formal instruments as well as the periodic instances to gather public and private actors to prevent, prepare and respond to emergencies (including PHC) needs to be strengthened in the country. |
|  | Convening private and public actors to plan, respond and learn to sustain PHC essential services during shocks, including contracting-out and other types of PPPs |  |
| Intersectoral engagement | National and local plans to prepare for emergencies that identifies actions at PHC level and network to provide non-health services (e.g. financial support, food aids, subsidies, guaranteed minimum income, etc.). | While there are existing intersectoral plans to prepare for external shocks, these plans do not always consider the role of PHC. |
|  | Basic services (drinkable water, energy, security) assuring mechanisms (e.g. back up). | Basic services are not assured in PHC across the territory, in particular in underserved areas. |
| Governance, production and use of data | Standardizing data (e.g. nominal ID, including electronic health record) to facilitate sharing between sectors (linked to empanelment). | A unique ID across health information systems is not widely available. |
|  | Creating risk and vulnerability maps using multisectoral data to inform action plans at the local level (geographical, disadvantaged groups | The country does not count with multisectoral maps of local risks and vulnerabilities to prepare and respond to emergencies. |
| Environmental considerations | Supporting the generation of national and sub-national plans that integrate a multi-hazard approach by developing territorial risk assessment impact studies that include the impact on primary care network and provisions | Plans that integrate a multi-hazard approach are a critical input to strengthen PHC climate resilience. |
| **FINANCING** | | |
| **Domain** | **Prioritized policy options** | **Rationale for their prioritization** |
| Financial governance | Emergency previously drafted financial decrees with clear protocols that enable targeted funding for urgent PHC activities (e.g., community health outreach, vaccinations, and essential services) | No emergency drafted decrees exist that consider the health sector, so the response to emergencies happens on an ad hoc basis. This affects at the same time the funds that are accessible for the response to shocks at local government and PHC levels. |
|  | Dedicated funds for health emergencies to enable local governments (based on their risk assessment) to quickly access and utilize resources when needed |  |
| Revenue raising and pooling of resources (where the money come from) | Diversify funding sources through different types of taxes (health-related, sin, and production taxes) to fund resilience activities | Considering that the country has a low public expenditure in health expenditure, it is a priority to boost increase the number of funding sources to ensure the creation of a resilience fund that sufficient funds exist to strengthen the baseline of PHC resilience and also for emergency funds that consider PHC |
|  | Emergency funds to invest in resilience, encouraging contributions from different local governments, potentially through multilateral or national/subnational arrangements (the OECD recommends investing 1.4% of GDP in resilience). |  |
| Management and allocation of funds | Benefit package for health crisis management that incorporates planning and forecasting to enable flexible resource allocation, adaptable to evolving needs during emergencies | No a-priori benefit package and accountability systems are in place to support a more standardized and organized financial response during external shocks. |
| Purchasing and payment mechanisms | Payroll adjustments to allow the hiring of temporary health care workers or mobile personnel, as well as task shifting to meet demand during emergencies | In general, flexible mechanisms for hiring health personnel during emergencies are not available (with the exception of the monetary incentives given during the COVID-19 pandemic).  At the same time, the supply-chains of medicines and vaccines need to be strengthened to have a more resilience health system. |
|  | Risk assessments to strengthen supply-chain management during emergencies |  |
